# Intravenous acetaminophen with morphine versus intravenous morphine alone for acute pain in the emergency room: protocol for a multicenter, randomized, placebo-controlled, double-blinded study (ADAMOPA)

**DOI:** 10.1186/s13063-022-06943-0

**Published:** 2022-12-15

**Authors:** Guillaume Cattin, Joel Jenvrin, Jean Benoit Hardouin, Céline Longo, Emmanuel Montassier

**Affiliations:** 1 Department of Emergency Medicine, CHU Nantes, F-44000 Nantes, France; 2 Nantes Université, Université de Tours, INSERM, MethodS in Patients-Centered Outcomes and HEalth Research, SPHERE, F-44000 Nantes, France; 3 Nantes Université, CHU Nantes, Methodology and Biostatistics Unit, F-44000 Nantes, France; 4 Nantes Université, CHU Nantes, Public Health Department, F-44000 Nantes, France; 5 Center for Research in Transplantation and Translational Immunology, UMR 1064, Nantes Université, CHU Nantes, INSERM, F-44000 Nantes, France

**Keywords:** Analgesia, Emergency room, Morphine, Pain, Paracetamol

## Abstract

**Background:**

In emergency medicine, pain is a frequent reason for consultation. However, there is a great variation in its management which is often insufficient. The use intravenous morphine alone or multimodal analgesia with paracetamol is recommended for severe pain. But robust data are lacking to justify the association of paracetamol with morphine versus morphine alone for pain management in the emergency room (ER). The aim of our study is therefore to assess if in patients with acute pain of moderate to severe intensity with a numerical verbal scale (NVS) ≥5 in the ER, the intravenous administration of morphine alone is not inferior to the administration of intravenous morphine combined with paracetamol at 30 min from the first administration of the study drug.

**Methods:**

ADAMOPA is a prospective, non-inferiority, multicenter, placebo-controlled, parallel-group, randomized (1:1), double-blind trial. Subjects will be enrolled in the ER if they experience moderate to severe, acute, non-traumatic, and traumatic pain, defined as an NVS ≥5. The primary endpoint will be the between-group difference in mean change in NVS pain scores among patients receiving the combination of intravenous morphine plus paracetamol or intravenous morphine given alone, measured from the time before administration of the study medication to 30 min later.

**Discussion:**

This trial will determine the clinical utility of the association of paracetamol with morphine for pain management in the emergency room. The ADAMOPA trial will be conducted in accordance with the International Council on Harmonization Good Clinical Practices.

**Trial registration:**

EudraCT number: 2019-002149-39. ClinicalTrials.gov identifier: NCT04148495. Date of trial registration: November 1, 2019.

**Supplementary Information:**

The online version contains supplementary material available at 10.1186/s13063-022-06943-0.

## Background

Pain is a frequent reason to go to an emergency room (ER) [1]. Overall, 78% of patients admitted to the emergency room suffer from pain, with a traumatic origin in 40% of cases for painful patients [2, 3]. Moreover, the percentage of patients reporting severe pain admitted to an ER rose from 25% in 2003 to 40% in 2008 [4].

There is a great variation in the management of acute pain in the ER with expert committees recommending the early use of intravenous morphine alone or as multimodal analgesia for severe pain [5]. In this context, paracetamol is commonly prescribed in the ER for pain management and this prescription of paracetamol will be continued during the hospitalization. However, the impact of paracetamol toxicity on public health is significant. In the United States, it is thought to be responsible for 40% of acute hepatic failure owing to liver toxicity [6]. There is also a significant degree of hepatic toxicity from paracetamol, particularly at the upper limit of standard analgesic doses [7]. Moreover, several studies have evaluated the association of paracetamol with opioids for analgesia in a postoperative context [8–11], in cancer patients [12–16], or in chronic pain [17]. However, robust data on the combination of opioids with other pain medications in the ER are lacking. Two studies compared morphine with paracetamol and morphine alone pain management in the ER [18, 19]. Unfortunately, these studies have significant methodological limitations and no robust conclusions can be made on the interest of the systematic combination of morphine and paracetamol for pain management in the ER.

## Methods and analysis

### Aims

The aim of our study will be to assess if, for patients admitted to the ER with acute moderate to severe pain with NVS ≥5, intravenous morphine given alone with an initial dose of 0.1 mg/kg is not inferior to the intravenous administration of morphine with an initial dose of 0.1 mg/kg combined with one gram of intravenous paracetamol evaluated 30 min after the first administration of the study drug.

### Study design

ADAMOPA is a prospective, multicenter, phase IV, placebo-controlled, double-blind, randomized, parallel-group trial, that will compare intravenous morphine given alone with the combination of intravenous morphine and paracetamol in patients admitted to the ER with acute pain of moderate to severe intensity with an NVS ≥5. The trial will be conducted in 10 hospitals in France.

Subjects meeting all eligibility criteria will be randomly assigned to 1 of the 2 treatment groups in a 1:1 ratio based on a computer-generated randomization list prepared before the beginning of the study and centralized in a web interface. Thus, the order of treatment allocation will be randomly assigned using a computer-generated randomization table, and a biostatistician who does not participate in the recruitment of patients will oversee the randomization. Randomization will be balanced using random permutation blocks and will be stratified by type of pain (i.e., traumatic or non-traumatic). Patients will then receive 1 of the 2 following treatments: intravenous morphine combined with intravenous paracetamol or intravenous morphine combined with placebo. Randomization kits will include either paracetamol or placebo. Paracetamol, administered at a dose of 1 gram through ready-to-use 100 mL intravenous bags or placebo (100 mL of Sodium Chloride 0.9%) will be double-blind administered to the patient, concomitantly with intravenous morphine (IV infusion: 20 min for both).

In both groups, a 0.1 mg/kg bolus dose of intravenous morphine will be administered with a maximum dose of 10 mg [2, 20–22]. Morphine will then be titrated every 10 min by bolus of 0.05 mg/kg, each bolus with a maximum dose of 5 mg per administration, until the patient has pain with an NVS at ≤ 3, or until the patient experiences a serious adverse event: severe hypotension, unconsciousness, respiratory depression requiring mechanical ventilation, or until a maximum total dose of 20 mg of morphine in the first 30 min is reached. For patients with a blood oxygen saturation level (SpO_2_) below 94% during the procedure, oxygen will be administered with a nasal cannula-delivering flow rate of 2 L/min and will be adapted based on SpO_2_ follow-up. If a patient has pain with an NVS ≥5 at 30 min, 45 min, 60 min, rescue analgesia will be administered to the patient for additional pain relief (earliest time: 30 min after initial bolus). Importantly, for the rescue analgesia, the choice of drugs and dose will be left to the discretion of the ER physician.

**Fig. 1:**
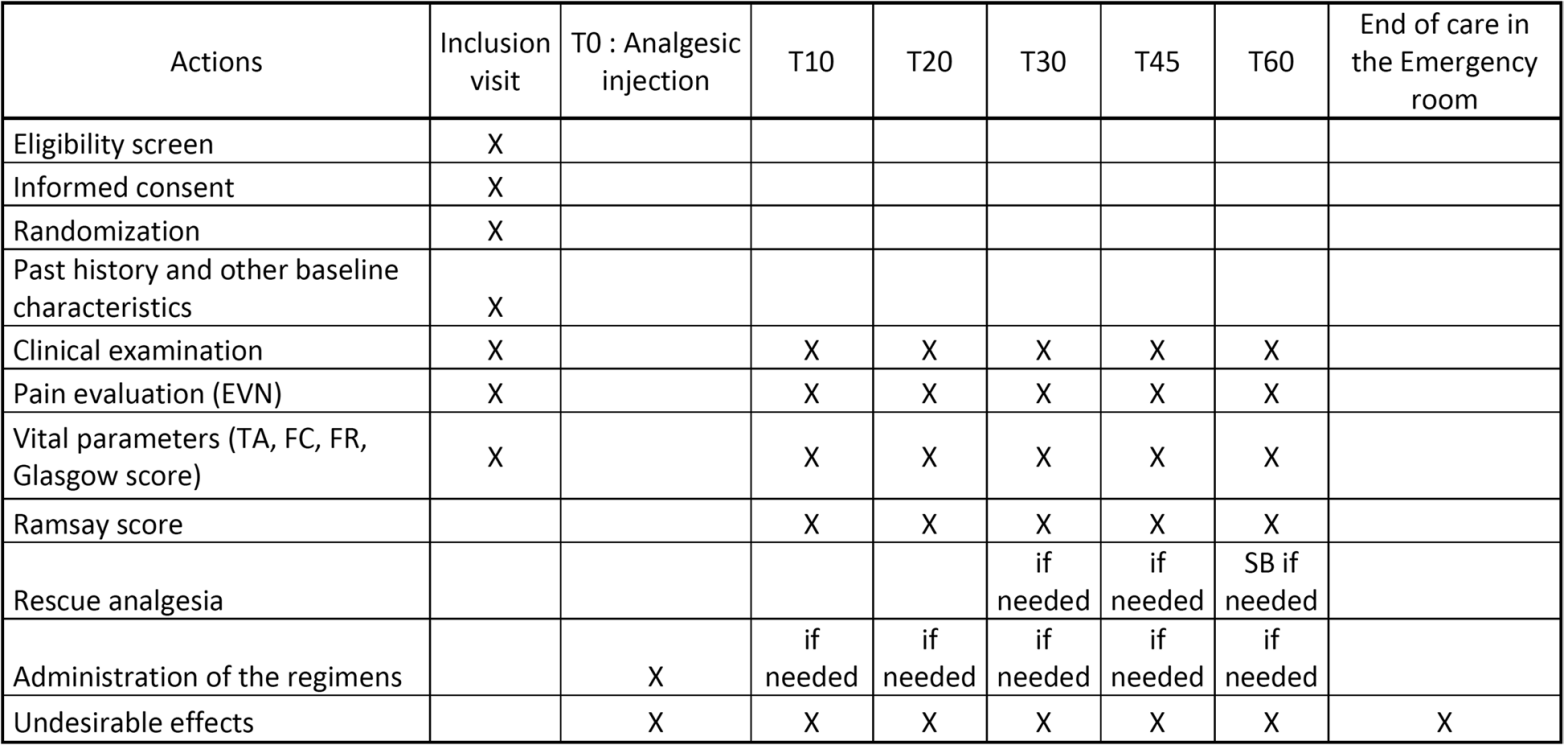
Standard Protocol Items: Recommendations for Interventional Trials (SPIRIT) Figure for the ADAMOPA trial. Schedule of enrollment, interventions, and assessments

The trial has been designed on the basis of the Consolidated Standards of Reporting Trials (CONSORT 2010) guidelines [23], and a SPIRIT Figure is provided (Fig. 1). A SPIRIT checklist file is attached (Additional file 1).

### Sample selection

Consecutive adults (18 years and older) will be enrolled in the study when admitted to the ER if they experience moderate to severe, acute, non-traumatic, or traumatic pain, defined as an NVS ≥5 on a standard 11-point numerical rating scale (0: no pain, to 10: as much pain as possible). Criteria for non-inclusion will be unstable vital signs (systolic blood pressure < 90 or > 200 mmHg, pulse rate < 50 or > 150 beats/min, and respiration rate < 10 or > 30 breaths/min with or without oxygen following standard guidelines, Glasgow Coma Scale score < 15), pregnancy, patients who weigh under 50 kilograms, patients requiring emergency fracture or joint reduction as part of the management of the traumatic pain, acute pulmonary edema, decompensated respiratory failure, acute coronary syndrome or ongoing unbalanced ischemic heart disease, acute intoxication with alcohol or a suspected illicit drug, patients who received analgesic treatment within the last 8 h for the current acute pain episode, inability to have venous access, known allergy to paracetamol or morphine, known history of renal or hepatic insufficiency, history of chronic pain during treatment, or patients taking buprenorphine, nalbuphine, or pentazocine.

The senior ER physician in charge of the patient will obtain informed consent. The patient will only be included in the ADAMOPA trial after reading the information note, signing, and dating the consent form (Additional file 2). The full protocol is attached as an additional file (Additional file 3).

### Measurements and planned outcomes

We hypothesize that in the management of patients with acute pain of moderate to severe intensity (NVS ≥5) in the ER, intravenous administration of morphine alone with an initial dose of 0.1 mg/kg will not be inferior to the administration of morphine with the same administration regimen combined with a dose of paracetamol 1g intravenously, 30 min from the first injection of the study drug.

The primary endpoint will be the between-group difference in mean change in NVS pain scores in the patients receiving the combination of intravenous morphine plus paracetamol or intravenous morphine given alone, measured from the time before administration of the study medication to 30 min later. The secondary endpoints will be (1) between-group difference in mean change in NVS pain scores in the patients receiving the combination of intravenous morphine plus paracetamol or intravenous morphine given alone, measured from the time before administration of the study medication to 15, 45, or 60 min later; (2) estimation of the cumulative dose of morphine in both groups in weight dose (mg/kg) during the first 30 min of administration of the study medication; (3) the frequency and intensity of adverse events (AE); (4) use of Naloxone; and (5) the number of prescriptions of rescue analgesic molecules at 30, 45, or 60 min. AEs reported spontaneously by the subject or observed by the investigator or the research staff will be recorded within the electronic case report forms. Investigators or research staffs could choose a predefined AE (injection pain, pruritus, dizziness, nausea, vomiting, other gastrointestinal complaints, hypotension, respiratory depression, respiratory depression requiring mechanical ventilation, somnolence, unconsciousness), or free text. Any AE, whether expected or unexpected, serious or not, will be real-time collected in the study eCRF. All serious AEs, whether expected or unexpected, will be reported immediately (from the day the of the investigator becoming aware of the event) to the sponsor by the mean of a notification form, and entered with the same terms and real-time in the eCRF. AEs will be presented as descriptive data in a table split by allocation arm.

### Provisions for ancillary and post-trial care

No provisions for ancillary or post-trials care were made. However, the sponsor has a liability insurance that provides cover for damage to research subjects through injury or death caused by the study. The contract complies with the French legal and regulatory provisions on research involving the human person and in particular with the provisions of law 88.1138 of 20/12/1988, modified by the subsequent texts in particular law n°2012-300 of March 5, 2012, and its implementing decree n°2016-1537 of November 16, 2016.

### Data collection

Prior to the beginning of the trial, study personnel will undergo training sessions on data collection and will be individually tested on data entry as well as outcome assessments. Study data will be collected and managed using Ennov Clinical electronic data capture tools at Nantes University Hospital. Ennov Clinical is a secure web-based application (electronic case report form), designed to support data capture for research studies, providing: (1) an intuitive interface for validated data entry; (2) audit trails for tracking data manipulation and export procedures; (3) automated export procedures for seamless data downloads to common statistical packages; and (4) procedures for importing data from external sources.

### Plans to promote participant retention and complete follow-up

To encourage participation in the trial, a great effort will be made to organize on-site visits or video conferences with researchers, hospital physicians, and nurses to facilitate investment in the research.

### Composition of the data monitoring committee, its role, and reporting structure

A data safety monitoring board or safety committee will not be established for this study since this is a low-risk study: (i) the study will not be performed in potentially fragile or vulnerable populations, (ii) the study endpoint will not require termination of the study before its planned completion, (iii) there are no *a priori* reasons for a particular safety concern, (iv) there is no prior information suggesting the potential for serious toxicity due to the study treatment, and (v) the study will not be performed in a population at elevated risk of death or other serious outcomes. However, on-site monitoring of all study sites will be performed regularly by an independent monitor of the Nantes University Hospital Research Unit (Nantes, France), as explained in more detail in the section “Frequency and plans for auditing trial conduct.

### Frequency and plans for auditing trial conduct

Monitoring will be carried out by the Research Division Promotion Department. A Clinical Research Associate will visit each site (investigator and dispensary) regularly to conduct quality control on the data reported in the case report forms. The on-site monitoring visits will be organized after making arrangements with the investigator. The CRAs will be able to consult on each site: the enrolled persons' data compilation records, the patients’ medical and nursing files, the investigator file. The monitors will review the source documents to determine whether the data reported in the electronic case report forms are complete and accurate. Also correct use of inclusion and exclusion criteria, data protection as well as medical research products will be verified.

### Who will be blinded

The randomized, double-blind design ensures that participants as well as the research team and care providers will be blinded for the intravenous acetaminophen intervention. The intravenous acetaminophen and placebo identification list will only be available to concerning research pharmacies.

### Procedure for unblinding if needed

During the trial, blinding will be maintained all the time, unless a suspected unexpected serious adverse reaction (SUSAR) occurs. The unblinded participant will exit the trial and the medical condition will be managed accordingly, and then will be recorded on the clinical report form.

### Plans for communicating important protocol amendments to relevant parties (e.g., trial participants, ethical committees)

In case of any substantial change in the protocol, an amendment will be submitted to the Ethics Committee (CPP OUEST I, October 10, 2019) that approved the version 1.0 of the protocol. Research team and participants will then be notified by the Nantes University Hospital Research Unit. If applicable, also the participant information letter, the informed consent form, study operator manuals, and trial registrations will be updated.

### Confidentiality

After informed consent, each participant included in the study will receive a unique study code with a number, which will be used for all study related documents and the electronic case report forms. The participant identification log will be stored separately and only accessible to the coordinating researchers at the concerning study sites and the responsible monitor.

### Sample size under non-inferiority hypothesis

To assess non-inferiority in the two subgroups of patients (i.e., traumatic and non-traumatic pain), with a non-inferiority margin of 1, standard deviation (SD) of 2.6, *α* = 5%/2, *β* = 10%, and a one-sided analysis, 572 patients are required (i.e., 143 in each group: morphine alone versus morphine plus paracetamol in traumatic patients, morphine alone versus morphine plus paracetamol in non-traumatic patients). The non-inferiority margin of 1 point of NVS that was selected was based in part on an expert opinion.

### Data analysis

Statistical analysis will be performed at the end of the study. Two independent analyses will be realized for the two groups of patients: traumatic or non-traumatic pain. For each quantitative or qualitative variable, we will report in each treatment group, the parameters of central tendency (mean and median) and dispersion (standard deviation, interquartile range, maximum and minimum values). No interim analysis is planned.

Since this is a non-inferiority study, analysis of the primary outcome will be performed on a per-protocol population. completed by a second analysis following the intention-to-treat principle. The non-inferiority could be claimed if the two analyses conclude to the non-inferiority.

The analyses will be based on a linear model explaining the difference of the NVS between baseline and 30 min by the center of recruitment (random effect), and the randomized group of the patients (fixed effect). The impact of the baseline value of the NVS will be included in the model (with two groups of values: 5 to 7 vs 8 to 10).

In order to take into account intercurrent events like death of the patient or impossibility to obtain a pain evaluation at 30 min for other reasons, a worst-case imputation will be realized on these patients. In these cases, the smallest observed difference of the NVS between baseline and 30 min among the patients with the same kind of pain and with the same randomized group will be imputed.

To conclude, we will compare the upper limit of the 97.5% unilateral confidence interval of the randomized group parameter to the non-inferiority margin fixed to 1 point. The selected type I error rate will be α of 0.025 for each group of patients, in order to obtain a global type I error of 5%. The analyses will be performed using Stata software (Stata Corp, TX, USA).

### Patient and public involvement

No patients were involved in the design process of this study, setting the research question, or the outcome measures nor were they involved in the analysis, interpretation, and writing of the results. Our findings from the trial will be shared with all participants, who will be provided with a lay abstract of our study and access to the full manuscript.

### List of study sites

Nantes University Hospital, La Roche sur Yon Hospital, Chateaubriand Hospital, Angers University Hospital, Bordeaux University Hospital, Grenoble University Hospital, Nancy University Hospital, La Pitié Salpetrière APHP University Hospital, Lariboisière APHP University Hospital, Saint Nazaire Hospital.

## Discussion

The trial is designed to compare the efficacy and safety of a combination of intravenous morphine with paracetamol and intravenous morphine given alone. This trial is multicenter including 10 hospitals in France and the study drug (paracetamol) will be administered in a double-blind manner. Moreover, the number of subjects lost to follow-up will be very low owing to the short duration of patient follow-up. This trial will make it possible to establish recommendations based on a multicenter randomized clinical trial. Two recent studies evaluating the analgesic efficacy of paracetamol added to hydromorphone in the management of patients with severe acute pain admitted to ER, demonstrated an analgesia not superior to treatment with hydromorphone alone [24, 25]. The main limitation of these studies is that hydromorphone, a molecule not commonly used in our departments, is prescribed at a fixed dose and not with a weight-dependent dose. Moreover, these studies did not take advantage of titrated and individualized intravenous morphine which could potentiate the effects of morphine and decrease the side effects of such therapy. A recent meta-analysis bringing together 5 randomized clinical trials found that paracetamol may be superior to morphine in the treatment of renal colic [26]. The main limitations are the significant heterogeneity of RCTs with different dosages of morphine and heterogeneous baseline pain intensity between each study. Moreover, several studies focused on renal colic, leaving aside all other pain that are commonly managed in the ED. Our trial will have practical consequences and may lead to a change in the management of patients admitted to ER with moderate to severe pain. Indeed, the demonstration of non-inferiority of morphine alone compared with the paracetamol with morphine combination will limit the use of paracetamol, de facto reducing the cost and the adverse events associated with the management of acute pain in the ER.

### Ethics and dissemination

The ADAMOPA trial is supported by a grant from the French Ministry of Health (PHRCI 2018 API18/N/066) and will be conducted in accordance with the International Council on Harmonization Good Clinical Practice adhering to the ethical principles of the Declaration of Helsinki (1964 and subsequent amendments). The funding source will have no role in the study design, data collection, data analysis, data interpretation, or report writing.

All of the authors have agreed to submit for publication. The trial is registered with the European Union Clinical Trials Register (EudraCT number 2019-002149-39) and ClinicalTrials.gov (NCT04148495). All trial documents and procedures have been reviewed and approved by the Ethics Committee (CPP OUEST I, October 10, 2019). Written informed consent will be obtained from all patients before the beginning of the trial.

The results of our trial will be actively disseminated through peer-reviewed journals, conference presentations, social media, broadcast media, print media, and the internet.

### Trial status

The trial commenced recruitment on 3 December 2019 according to protocol version 1.0, estimated study completion date: June 2022

### Trial registration

The trial is registered with the European Union Clinical Trials Register (EudraCT number 2019-002149-39) and ClinicalTrials.gov (NCT04148495). Date of trial registration: November 1, 2019.

## Supplementary Information

**Additional file 1.** SPIRIT Checklist.

**Additional file 2.** Consent form.

**Additional file 3.**

## Data Availability

The research team will have access to the final trial dataset which will be hosted by the Nantes University Hospital Research Unit. The investigator will archive all study data for at least 25 years after the end of the study. At the end of the study, the investigator shall also receive a copy of the data for each person in the investigator’s center sent by the sponsor.

## Abbreviations

CONSORT: Consolidated Standards of Reporting Trials
NVS: Numerical verbal scale
