# Supplementary material for: Intravenous acetaminophen with morphine versus intravenous morphine alone for acute pain in the emergency room: protocol for a multicenter, randomized, placebo-controlled, double-blinded study (ADAMOPA)
Source: Trials. 2022 Dec 15;23:1016. doi: 10.1186/s13063-022-06943-0 (PMC9756523; doi:10.1186/s13063-022-06943-0)
Supplement: Supplementary file 2 — Additional file 2. Consent form. [file 13063_2022_6943_MOESM2_ESM.pdf]

| CENTRE HOSPITALIER UNIVERSITAIRE DE NANTES<br>Version n° 1.1<br>Date : 17/09/2019 | **ANNEXE 4**<br>**Certificate of consent to research**<br>**« ADAMOPA trial: Intravenous Acetaminophen with Morphine versus Intravenous Morphine Alone for Acute Pain in the Emergency Room: a Multicenter, Randomized, Placebo-controlled, Double-blinded Study»**<br>**Responsible party*: CHU de Nantes***<br>***N° EudracT :* 2019-002149-39.** |
|---|---|

I, the undersigned
Me, M. (delete as appropriate) (first name, LAST NAME)…………………………………………………………………………
…………………………………………………………………………………………………………………………………………,

Date of Birth: …../……/……..
**freely and voluntarily accepts to participate in the research referenced above, coordinated by the Doctor X,** and organized by the Nantes University Hospital, promoter of research.

**Being heard that :**

- he investigator who informed me and answered all of my questions clearly, told me that my participation is free and that I can withdraw from the research at any time.
- I certify that I am not subject to protective measures (guardianship, curatorship, safeguard of justice), in addition I confirm that I am affiliated or benefit from a social security scheme.
- I was previously given an information note on this research specifying its purpose, methodology, expected benefits and foreseeable risks.
- I may have information from the investigator, during or after the research, of the information he has regarding my health.
- If the number of people foreseen in the study has been reached, I could ultimately not be included in the study even though I signed a consent. In this specific case, I will no longer be followed within the framework of the study and the data concerning me as well as the blood samples if necessary will be destroyed. This will in no case prejudice the quality of my care.
- I am fully aware that I can withdraw my consent to my participation in this research at any time, whatever my reasons and without bearing any responsibility, but I undertake in this case to inform the investigator . The fact of no longer participating in this research will not affect my relations with this investigator, nor the quality of the care that will be given to me.
- If I wish, the support person I have designated can assist me in my efforts and can attend medical interviews to help me in my decisions.

• I accept that my attending physician will be informed of my participation in the research:

☐ Yes, I accept ☐ No i refuse

- I may at any time request additional information from the investigator.
- If I wish, at the end, I will be informed by the investigator of the overall results of this research.
- My consent in no way relieves the investigator and the promoter of all their responsibilities and I retain all of my rights guaranteed by law.
- I accept that the data recorded during this research may be subject to computerized processing by the promoter or on his behalf, and I certify that I have been informed of all my rights concerning my personal data according to the terms and conditions described in the information note in force, which was sent to me for this protocol.

| **PERSON INCLUDED IN THE RESEARCH** | |
|---|---|
| **Dated :**<br>**...... / ..... / ………..** | **Signature :** |

| **INVESTIGATOR: I certify that I have fully explained to the signatory the purpose, the procedures and the potential risks of the research** | | |
|---|---|---|
| **Dated :**<br>**...... / ..... / ………..** | **NAME:** | **Signature :** |

This document must be produced in 3 original copies: the first must be kept by the investigator and the second is given to the person giving his consent. In the event of a duplicate, the original is kept by the investigator and a copy is given to the person who gave his consent. In the event of a triplicate, the promoter will collect one of the duplicate consents in sealed envelopes throughout the study.
