# Supplementary material for: Intravenous acetaminophen with morphine versus intravenous morphine alone for acute pain in the emergency room: protocol for a multicenter, randomized, placebo-controlled, double-blinded study (ADAMOPA)
Source: Trials. 2022 Dec 15;23:1016. doi: 10.1186/s13063-022-06943-0 (PMC9756523; doi:10.1186/s13063-022-06943-0)

Protocole *ADAMOPA*

Eudract : n°
Ref : RC/

«*Analgésie des douleurs aiguës aux urgences : Essai randomisé de non infériorité de la Morphine seule versus l'association Morphine et Paracétamol*»

Investigateur Coordonnateur :
Dr Céline LONGO
Service des Urgences- CHU Nantes
1 place Alexis Ricordeau
44093 Nantes
Tel : +33 (0) 2 53 48 20 44
Email : celine.longo@chu-nantes.fr

Méthodologiste :
Jean-Benoît HARDOUIN
Institut de recherche en Santé 2 EA4275-SPHERE
18 boulevard Benoni-Goullin
44000 Nantes

Promoteur :

CENTRE HOSPITALIER
UNIVERSITAIRE DE NANTES

CHU de Nantes
Direction des Affaires Médicales et de la Recherche

5, allée de l'île Gloriette
44 093 Nantes cedex 01 (FRANCE)
Tel : 02 53 48 28 35
Fax : 02 53 48 28 36

PAGE DE SIGNATURE

SIGNATURE DU PROMOTEUR

Le promoteur s'engage à réaliser cette étude selon toutes les dispositions législatives et réglementaires dont pourrait relever la recherche et selon le protocole.		
**Nom et Fonction du représentant signataire : Pour le promoteur et par délégation du Directeur Général, la Directrice des Affaires Médicales et de la Recherche**	**Date :**	**Signature :**

SIGNATURE DES INVESTIGATEURS

J'ai lu l'ensemble des pages du protocole de l'essai clinique dont le CHU de Nantes est le promoteur. Je confirme qu'il contient toutes les informations nécessaires à la conduite de l'essai. Je m'engage à réaliser l'essai en respectant le protocole et les termes et conditions qui y sont définis. Je m'engage à réaliser l'essai en respectant :

- les principes de la “Déclaration d'Helsinki”,
- les règles et recommandations de bonnes pratiques cliniques internationales (ICH) et françaises (règles de bonnes pratiques cliniques pour les recherches biomédicales portant sur des médicaments à usage humain)
- la règlementation européenne et/ou la législation nationale et la réglementation relative aux essais cliniques,

Je m'engage également à ce que les investigateurs et les autres membres qualifiés de mon équipe aient accès à ce protocole ainsi qu'aux documents relatifs à la conduite de l'essai pour leur permettre de travailler dans le respect des dispositions figurant dans ces documents.

**Investigateur coordonnateur**	**Nom :**	**Date :**	**Signature :**
**Investigateur principal**	**Nom et établissement :**	**Date :**	**Signature :**

LISTE DES ABREVIATIONS

ANSM	Agence Nationale de Sécurité du Médicament et des produits de santé
AMM	Autorisation de Mise sur le Marché
ARC	Attaché de Recherche Clinique (moniteur)
BPC	Bonnes Pratiques Cliniques
CPP	Comité de Protection des Personnes
CNIL	Commission Nationale de l'Informatique et des Libertés
CIS	Comité indépendant de surveillance
eCRF	Electronic Case Report Form (cahier d'observation électronique)
EMA	European Medicines Agency
EvIG	Evènement Indésirable Grave
EIG	Effet Indésirable Grave
EIGI	Effet Indésirable Grave Inattendu
FIH	First-in-Human = Essai de 1ère administration chez l'homme
MR	Méthodologie de Référence CNIL
RCP	Résumé des Caractéristiques d'un Produit
SUSAR	Suspected Unexpected Serious Adverse Reaction
TEC	Technicien d'Etude Clinique

TABLE DES MATIERES

Contenu

PAGE DE SIGNATURE **2**

LISTE DES ABREVIATIONS **3**

TABLE DES MATIERES **4**

INTRODUCTION **7**

1. JUSTIFICATION DE L'ETUDE **8**

1.1. POSITIONNEMENT DE LA RECHERCHE 8
1.2. BENEFICES ET RISQUES POUR LES PERSONNES SE PRETANT A LA RECHERCHE 18
1.2.1. Bénéfices 18
1.2.2. Risques 20
1.2.3. Balance bénéfices / risques 22

2. FAISABILITE DE L'ETUDE **23**

3. OBJECTIFS ET CRITERES DE JUGEMENT **24**

3.1. OBJECTIF ET CRITERE D'EVALUATION PRINCIPAL 24
3.1.1. Objectif principal 24
3.1.2. Critère d'évaluation principal 24
3.2. OBJECTIF ET CRITERE D'EVALUATION SECONDAIRE 25
3.2.1. Objectif(s) secondaire(s) 25
3.2.2. Critère(s) d'évaluation secondaire(s) 26

4. DESIGN DE LA RECHERCHE : **27**

4.1. METHODOLOGIE GENERALE DE LA RECHERCHE 27
4.2. SCHEMA DE L'ETUDE 27
4.3. DESCRIPTION ET JUSTIFICATION DU SCHEMA THERAPEUTIQUE 28
4.4. DUREE DE L'ETUDE 29

5. POPULATION ETUDIEE **30**

5.1. DESCRIPTION DE LA POPULATION 30
5.2. CRITERES D'INCLUSION 30
5.3. CRITERES DE NON-INCLUSION 31

6. TRAITEMENTS UTILISES PENDANT L'ETUDE **32**

6.1. DESCRIPTION ET MODALITES D'ADMINISTRATION 32
6.1.1. Médicament(s) expérimental (aux) / comparateur 32
6.1.2. Médicaments auxiliaires 35
6.2. ALLOCATION DES TRAITEMENTS ET MISE EN INSU 36
6.2.1. Allocation des traitements : procédures de randomisation 36
6.2.2. Méthode de mise en insu 37
6.3. MEDICAMENTS ET TRAITEMENTS AUTORISES ET INTERDITS 38
6.3.1. Traitements autorisés 38
6.3.2. Traitements non autorisés 38
6.3.3. Traitement d'urgence 38
6.4. METHODES DE SUIVI DE L'OBSERVANCE AU TRAITEMENT 39
6.5. CIRCUIT DES MEDICAMENTS EXPERIMENTAUX 39
6.5.1. Circuit général 39

6.5.2. Conditions de stockage des médicaments expérimentaux : *39*
6.5.3. Procédure de levée d'aveugle : *40*

7. DEROULEMENT DE L'ETUDE 41

7.1. CALENDRIER DE L'ETUDE 41
7.1.1. V0 : Sélection des patients et inclusion : *41*
7.1.2. V1 : Randomisation des patients *41*
7.1.3. V2 : Suivi des patients à T10, T20, T30, T45 et T60 ...... *43*
7.1.4. V3 : Visite de fin d'étude à la fin de prise de la prise en charge aux urgences *44*
7.2. IDENTIFICATION DE TOUTES LES DONNEES SOURCES NE FIGURANT PAS DANS LE DOSSIER MEDICAL 46
7.3. REGLES D'ARRET DE LA PARTICIPATION D'UNE PERSONNE 46
7.3.1. Critères d'arrêt prématuré de la participation d'une personne à la recherche *46*
7.3.2. Procédures d'arrêt prématuré de la participation d'une personne à la recherche *47*
7.3.3. Critères d'arrêt d'une partie ou de la totalité de la recherche (hors considérations biostatistiques) *47*
7.4. MODALITE DE PRISE EN CHARGE DES PATIENTS A LA FIN DE LA RECHERCHE 48
7.5. INDEMNISATION 48

8. DATA MANAGEMENT ET STATISTIQUES 49

8.1. RECUEIL ET TRAITEMENT DES DONNEES DE L'ETUDE 49
8.1.1. Recueil, traitement et circulation des données *49*
8.1.2. Identification du participant *49*
8.2. STATISTIQUES 50
8.2.1. Description des méthodes statistiques prévues, y compris du calendrier des analyses intermédiaires prévues *50*
8.2.2. Justification statistique du nombre d'inclusions *52*
8.2.3. Degré de signification statistique prévu *52*
8.2.4. Critères statistiques d'arrêt de la recherche *52*
8.2.5. Méthode de prise en compte des données manquantes, inutilisées ou non valides *52*
8.2.6. Gestion des modifications apportées au plan d'analyse de la stratégie initiale *53*
8.2.7. Choix des personnes à inclure dans les analyses *53*

9. PHARMACOVIGILANCE ET GESTION DES EVENEMENTS INDESIRABLES 54

9.1. DEFINITIONS 54
9.2. PARAMETRES D'EVALUATION DE LA SECURITE 55
9.2.1. Critères d'évaluation particuliers liés à la sécurité *55*
9.2.2. Méthodes et calendrier prévus pour mesurer, recueillir et analyser les paramètres d'évaluation de la sécurité *56*
9.3. LISTE DES EI ATTENDUS 56
9.3.1. Concernant le traitement à l'étude : le comparateur *56*
9.3.2. Concernant le protocole : *58*
9.3.3. Concernant la pathologie : *58*
9.4. GESTION DES EVENEMENTS INDESIRABLES 59
9.4.1. Recueil des EvI/EI *59*
9.4.2. Notification des EvIG/EIG *59*
9.4.3. Période de notification *60*
9.4.4. Comité indépendant de surveillance (CIS) *60*
9.5. MODALITES ET DUREE DU SUIVI DES PERSONNES SUITE A LA SURVENUE D'EVENEMENTS INDESIRABLES 60

10. ASPECTS ADMINISTRATIFS ET REGLEMENTAIRES 61

10.1. DROIT D'ACCES AUX DONNEES ET DOCUMENTS SOURCE 61
10.2. MONITORING DE L'ESSAI 61
10.3. INSPECTION / AUDIT 62
10.4. CONSIDERATIONS ETHIQUES 62
10.4.1. Consentement éclairé écrit 62
10.4.2. Modalités de recueil du consentement en cas d'urgence 62
10.4.3. Comité de Protection des Personnes 63
10.5. DECLARATION AUX AUTORITES COMPETENTES 63
10.6. AMENDEMENTS AU PROTOCOLE 63
10.7. FICHIER DES PERSONNES SE PRETANT AUX RECHERCHES BIOMEDICALES 63
10.8. FINANCEMENT ET ASSURANCE 63
10.9. REGLES RELATIVES A LA PUBLICATION 63
10.10. DEVENIR DES ECHANTILLONS BIOLOGIQUES 64
10.11. ARCHIVAGE 64

LISTE DES ANNEXES 1

ANNEXE 1 : BIBLIOGRAPHIE 2

ANNEXE 2: LISTING DES INVESTIGATEURS 9

ANNEXE 3: RESUME DU PROTOCOLE 10

ANNEXE 4: ECHELLE VERBALE NUMERIQUE 13

Annexe 5 : SCORE DE RAMSAY…………………………………………………………14

ANNEXE 6 : RCP CHLORURE DE SODIUM 15

ANNEXE 7: RCP PARACETAMOL 23

ANNEXE 8 : RCP MORPHINE……………………………………………………………32
ANNEXE 9 : DETAILS DU BUDGET ADAMOPA……………………………………….41
ANNEXE 10: LETTRES D'ENGAGEMENT DES CENTRES ASSOCIES……………45

INTRODUCTION

La lutte contre la douleur une priorité de santé publique. En médecine d'urgence, la douleur est un motif fréquent de consultation, mais est insuffisamment prise en charge dans ce contexte. En effet, il existe une grande variation dans la prise en charge de la douleur aiguë aux urgences. En France, la Société Française de Médecine d'Urgence (SFMU) et la Société Française d'Anesthésie et de Réanimation (SFAR) ont publié des recommandations formalisées d'experts sur la prise en charge de la douleur en urgence en 2010. Il est recommandé de recourir pour les douleurs intenses, aux morphiniques intraveineux seuls ou en analgésie multimodale en y associant le paracétamol. Cependant, les données de la littérature sont très modestes pour justifier l'association du paracétamol à la morphine versus la morphine seule dans la prise en charge de la douleur aux urgences.

Ainsi, le but de notre recherche est de fournir des preuves robustes, à l'aide d'un essai clinique multicentrique, randomisé, contrôlé, en aveugle, de l'intérêt ou non d'utiliser l'association morphine et paracétamol versus la morphine seule dans la prise en charge de la douleur modérée à sévère aux urgences. A notre connaissance, aucune étude d'un tel niveau de preuve n'a été publiée ou est en cours. Ainsi, notre étude pourrait fournir des données solides pour éviter aux patients douloureux aux urgences d'être exposé à la fois au paracétamol et à la morphine.

1. JUSTIFICATION DE L'ETUDE

1.1. POSITIONNEMENT DE LA RECHERCHE

La douleur : un des motifs de consultation les plus fréquents aux urgences

La douleur, souvent appelée « cinquième signe vital », (1) est un motif très fréquent de consultation aux urgences (2). En effet, 78% des patients pris en charge dans les services d'urgences sont douloureux, que cette douleur soit en lien avec le motif de consultation ou qu'elle soit provoquée par les soins (3). Globalement, la douleur d'origine traumatique représente 40% des patients douloureux admis aux urgences, les douleurs abdominales et urologiques représentant quant à elles chacune 13% (4). En outre, chez les patients admis aux urgences pour un traumatisme, la prévalence de la douleur est d'environ 90% (5). De plus, le pourcentage de patients rapportant une douleur sévère admis aux urgences est passé de 25% en 2003 à 40% en 2008 (6).

La lutte contre la douleur : une priorité de santé publique

Une prise en charge rapide, efficace et sécurisée de la douleur constitue une composante clé de la médecine d'urgence. Cependant, la prise en charge de la douleur aux urgences n'est pas optimale *(7, 8).* Une étude norvégienne a rapporté que les femmes ont une forte probabilité de ne pas être interrogées sur leur douleur, ainsi que les patients présentant une détresse vitale *(9).* De plus, un rapport, publié en 2011 a soulevé que la prise en charge de la douleur aiguë était souvent inadéquate *(Institute of Medicine Committee on Advancing Pain Research, Care, and Education, 2011)*. Dans une étude en Norvège, Dale and Bjørnsen rapportaient que seulement 14% des patients ayant une douleur modérée à sévère recevaient un traitement antalgique *(9).* Todd et al rapportaient que seulement 60% des patients ayant des douleurs aux urgences recevaient une analgésie alors que trois quarts de ces patients présentaient des douleurs modérées à sévères *(10).* En France, en

2011, une étude évaluait que 30% des patients sortants des urgences présentaient des douleurs modérées ou sévères non soulagées *(3).*

« La douleur aiguë est fréquente en médecine d'urgence et insuffisamment prise en charge dans ce contexte ». (11) Ainsi, la lutte contre la douleur est depuis plusieurs années une priorité de santé publique : ***« La mise en place de directives et recommandations nationales témoigne de la volonté des institutions, et des professionnels, d'optimiser la prise en charge de la douleur ». (12)***

La morphine : principal traitement des douleurs modérées à sévères aux urgences

Aux urgences, il existe une grande variation dans la prise en charge de la douleur aiguë, mais majoritairement, c'est la morphine qui est administrée en première intention pour soulager une douleur modérée ou sévère *(13, 14, 15, 16, 17, 18, 19, 20).* Aux Etats-Unis, 37% des patients admis pour une douleur reçoivent un traitement morphinique aux urgences *(6).* C'est d'ailleurs l'opioïde parentéral le plus couramment utilisé dans les services d'urgence aux États-Unis *(2).*

En France, la Société Française de Médecine d'Urgence (SFMU) et la Société Française d'Anesthésie et de Réanimation (SFAR) ont publié des recommandations formalisées d'experts sur la prise en charge de la douleur en urgence en 2010. Il y est recommandé de recourir d'emblée aux morphiniques intraveineux seuls ou en analgésie multimodale pour les douleurs intenses *(11).*

Place du paracétamol dans la prise en charge de la douleur aux urgences

Le paracétamol est couramment prescrit aux urgences dans la prise en charge de la douleur. Selon le rapport de l'Agence Nationale de Sécurité du Médicament de 2014, le paracétamol est la 29ème substance active la plus vendue à l'hôpital, représentant une dépense annuelle que l'on peut estimer à 25 millions d'euros à l'échelle nationale. L'agence américaine du médicament (FDA) autorise

depuis 2010 son utilisation dans la douleur légère à modérée, ainsi que pour la gestion de la douleur modérée à sévère, en appoint des analgésiques opioïdes *(21)* L'Organisation Mondiale de la Santé (OMS) classe le paracétamol comme antalgique de palier I. En France, les recommandations préconisent l'utilisation d'analgésiques de paliers I isolément pour traiter les douleurs faibles à modérées, ou en association dans le cadre d'une analgésie multimodale *(11).*

Cependant, le paracétamol n'est pas dénué d'effets secondaires potentiellement graves. En effet, l'impact de la toxicité du paracétamol sur la santé publique est important. Aux États-Unis, il serait responsable, du fait de sa toxicité hépatique, de 40% des insuffisances hépatiques aiguës (IHA) (22). En Europe, une étude évaluant tous les cas d'IHA entraînant un enregistrement pour transplantation, a montré qu'une surdose de paracétamol était responsable d'1/6ème des cas d'IHA au sein de 7 pays européens (23). Dans une étude prospective américaine, La part d'IHA secondaire à une intoxication involontaire au paracétamol représentait 48% des IHA *(24).* En outre, une étude a montré que le niveau de connaissance des patients sur le risque de toxicité hépatique ainsi que sur la dose maximale quotidienne possible est fragmentaire *(25).* **Ainsi, les études indiquent un degré important de toxicité du paracétamol, en particulier à la limite supérieure des doses analgésiques standards (26).** En dehors des situations de surdosage, l'IHA au paracétamol serait deux fois plus fréquente que l'IHA après exposition aux AINS *(27).*

Une question sans réponse à ce jour : morphine seule ou association morphine et paracétamol pour la prise en charge de la douleur aux urgences ?

De nombreuses études ont démontré l'intérêt de l'usage du paracétamol versus placebo dans l'analgésie *(28, 29, 30, 31, 32, 33).* Cependant, l'ensemble de ces études concernaient des patients douloureux pris en charge en période post-opératoire, et non pas dans le cadre de la médecine d'urgence.

Depuis 2014, il est recommandé d'appliquer des protocoles d'analgésie multimodale, reposant sur la combinaison de plusieurs analgésiques, afin d'obtenir

une association au moins additive, voire synergique. Toujours en période post-opératoire, l'analgésie multimodale a ainsi pour but d'associer différentes molécules ayant un mécanisme d'action différent, avec l'objectif de renforcer l'analgésie et/ou diminuer les besoins en analgésiques et leurs effets secondaires. Il est ainsi recommandé d'associer au moins un analgésique non morphinique lorsque la morphine est utilisée en postopératoire par voie systémique. Une méta-analyse de 52 études regroupant 4893 patients postopératoires a montré que l'association morphine et paracétamol permettait une réduction de la consommation d'opioïdes par le patient à 24 heures de sa prise en charge chirurgicale de 20% sans pour autant être plus efficace sur la réduction de la douleur que la morphine seule et sans réduction des effets indésirables de la morphine *(34)*. Une autre méta-analyse regroupant 7 essais et 256 patients a retrouvé cet effet épargneur de morphine avec une réduction de 20% sur la quantité d'opioïdes consommés à 24 heures *(35).* Cependant, il n'était pas rapporté de différence significative sur la survenue d'effets indésirables. Une autre méta-analyse de 36 études regroupant 3896 patients a montré que 37% des patients recevant du paracétamol en postopératoire associé à de la morphine ont éprouvé au moins 50% de soulagement de la douleur en 4 heures contre 16% chez ceux recevant le placebo associé à la morphine. Les patients recevant du paracétamol ont eu besoin de 30% de morphine en moins sur 4 heures par rapport à ceux recevant le placebo. Cependant, cela ne s'est pas traduit non plus par une réduction des effets indésirables induits par les opioïdes *(36).*

Des études plus récentes viennent appuyer ces résultats évoquant un effet épargneur de l'association morphinique-paracétamol, toujours dans ce contexte postopératoire : réduction de 27% de la consommation d'opioïdes dans le cadre d'une chirurgie thoracique, *(37)* réduction significative de 19% de la consommation d'opioïdes en post-césarienne *(38).* Cependant il n'y a pas toujours pas de différence concernant les effets indésirables liés aux opioïdes entre les deux groupes dans ces deux dernières études.

Pourtant malgré cet effet épargneur de morphinique, les réactualisations de la recommandation sur la prise en charge des douleurs postopératoires de la Société Française d'Anesthésie et de Réanimation (SFAR) préconisent l'utilisation d'AINS en association à la morphine au lieu du paracétamol, en absence de contre-indication ou de risque hémorragique important *(39).* **Car des**

arguments existent pour remettre en cause l'association systématique du paracétamol aux opioïdes pour l'analgésie dans un contexte postopératoire. Maund *et al.* montrait déjà en 2011 lors d'une revue de la littérature de 54 essais que les AINS avaient plus d'effet sur la réduction de la consommation de morphine pendant les premières 24 heures postopératoires que le paracétamol. Une étude américaine rétrospective retrouvait que le paracétamol ne diminuait pas significativement l'utilisation d'opioïdes postopératoires chez les patients ayant eu une intervention chirurgicale au genou. De plus, il y avait une tendance à l'augmentation de l'utilisation d'opioïdes dans le groupe paracétamol par rapport au groupe placebo *(40).* Chez des patients ayant eu une craniotomie, randomisés pour recevoir soit 1 gramme de paracétamol, soit un placebo, les besoins en opioïdes dans les 24 premières heures étaient similaires dans les 2 groupes *(41).* Dans un autre essai monocentrique en double aveugle où 147 patients américains ayant eu une chirurgie cardiaque ont été randomisés pour recevoir 1 gramme de paracétamol par voie intraveineuse toutes les 6 heures ou un placebo à partir de la fermeture sternale, le paracétamol était non inférieur au placebo sur la consommation d'opioïdes *(42).* De même que dans cet autre essai clinique américain monocentrique, randomisé, en double aveugle, ni l'administration intraveineuse du paracétamol ni celle par voie orale ne procuraient une analgésie supplémentaire dans la période postopératoire immédiate lorsqu'ils étaient administrés en complément à la morphine chez des patients ayant subis une arthroplastie totale du genou par rapport au groupe placebo *(43).*

Dans une étude dans un service de réanimation, le paracétamol (1 gramme de paracétamol par voie intraveineuse toutes les 6 heures pendant les 3 premiers jours d'admission) n'a eu aucun effet significatif sur la diminution des douleurs et le besoin en morphine chez les patients intubés admis pour une prise en charge non chirurgicale, par rapport au groupe placebo *(44).*

Plusieurs études ont aussi remis en cause l'intérêt de l'association morphine et paracétamol dans la prise en charge de la douleur chez les patients cancéreux *(45, 46, 47, 48, 49)*. De plus, l'association paracétamol et morphine versus morphine seule est aussi remise en cause par une étude dans le traitement de la lombalgie chronique *(50).*

Ainsi, toutes ces études relativisent l'intérêt d'une association systématique du paracétamol à un traitement morphinique versus la morphine seule. Cette vue d'ensemble remet en question la pratique consistant à utiliser systématiquement le paracétamol associé à la morphine pour des douleurs postopératoires aiguës, chroniques cancéreuses ou non cancéreuses.

Nous n'avons pu identifier que deux études comparant la morphine à l'association paracétamol et morphine dans la prise en charge de la douleur aux urgences. Cependant, ces études ont des limites méthodologiques importantes qui ne permettent pas de conclure, à ce jour, sur l'intérêt ou non d'une association paracétamol et morphine versus morphine seule. Un essai contrôlé randomisé en double aveugle a été réalisé chez 87 patients âgés de 21 à 65 ans, ayant une colique hépatique et recrutés dans deux services d'urgence iraniens. Ils ont été randomisés en deux groupes, le groupe A recevant 0,05 mg/kg morphine par voie intraveineuse associée à 1 gr de paracétamol par voie intraveineuse et le groupe B recevant 0,1 mg/kg de morphine par voie intraveineuse associée au placebo. Les scores de douleur ont été enregistrés en utilisant l'échelle visuelle analogique (EVA) à l'admission, puis à 15 et 30 minutes après l'administration du médicament. A 15 minutes après l'administration du médicament, la différence moyenne était de -0,35 (*IC* 95%: -1,15 à 0,45; p = 0,38) entre les 2 groupes et à 30 minutes, la différence moyenne était de -0,48 (*IC* 95%: -1,20 à 0,24; p = 0,19) *(51).* Cependant, les limites méthodologiques de cette étude étaient importantes : une restriction dans l'âge des patients inclus, l'inclusion de patients peu douloureux ne justifiant pas d'un traitement par morphine (critère d'inclusion EVA $\geq$ 3), l'inclusion uniquement de patients ayant une colique hépatique, un faible nombre de patients inadéquat pour évaluer la survenue d'effets secondaires, les doses de morphiniques utilisées étaient différentes entre les 2 groupes à l'étude, le délai non précisé entre l'entrée du patient aux urgences et sa randomisation (après une évaluation clinique par le médecin, attente des résultats de laboratoire et une échographie abdominale selon les critères d'inclusion) durant lequel le patient ne recevait aucun traitement antalgique. Or le lien entre la précocité du traitement et l'efficacité de l'analgésie ont bien établi *(52, 53).*

Un autre essai clinique a inclus 153 patients âgés de 15 à 60 ans se présentant avec une fracture osseuse, randomisés dans deux groupes recevant du

sulfate de morphine (5 mg) par voie intraveineuse ou de l'oxycodone (10 mg) par voie orale associé à du paracétamol (1 gramme) par voie intraveineuse. Les scores de douleur étaient similaires entre les groupes avant le traitement, à 30 et 60 minutes après le traitement. Les patients du groupe paracétamol/oxycodone ont présenté plus de nausées que les patients du groupe sulfate de morphine *(54).* Cependant, les limites méthodologiques de cette étude étaient importantes : une restriction dans l'âge des patients inclus, l'inclusion uniquement de patients ayant une douleur traumatique, et enfin les 2 groupes de traitement ne recevaient pas le même opioïde. Les différents profils d'opioïdes utilisés biaisent l'analyse de l'effet antalgique du paracétamol dans le cadre des douleurs aiguës dans cette étude. Selon les recommandations de la Haute Autorité de Santé (HAS), le rapport d'équianalgésie est le suivant : 10 mg d'oxycodone par voie orale sont équivalents à 20 mg de morphine orale, ce qui revient à donner plus que les 5 mg de morphine intraveineuse administrés dans cette étude. Il est d'autant plus surprenant que les auteurs ne retrouvent pas de supériorité du groupe oxycodone-paracétamol sur le groupe morphine concernant l'efficacité antalgique au vu des concentrations d'opioïdes différentes entre chaque groupe.

Ainsi, il n'existe à l'heure actuelle aucune donnée robuste sur l'intérêt d'associer systématiquement le paracétamol à la morphine versus la morphine seule dans la prise en charge de la douleur aux urgences.

Quel est le but notre recherche ?

Au vu de cette revue détaillée de la littérature, des questions restent actuellement sans réponse : avons-nous raison d'associer la morphine au paracétamol dans la prise en charge de la douleur aux urgences plutôt que de prescrire la morphine seule ? Est-ce que cela diminue la consommation de morphinique par le patient lorsque nous prescrivons l'association morphine-paracétamol plutôt que la morphine seule ? Est-ce que le patient est mieux et plus rapidement soulagé lorsque nous prescrivons l'association morphine-paracétamol plutôt que la morphine seule ? Est-ce que le patient présente moins d'évènements indésirables lorsque nous prescrivons l'association morphine-paracétamol plutôt que la morphine seule ? Le paracétamol ayant des effets secondaires potentiellement

graves, est-il licite de l'associer de façon systématique à la morphine dans le cadre de la prise en charge de la douleur aux urgences ?

Ainsi, le but de notre étude est de fournir des preuves robustes, à l'aide d'un essai clinique multicentrique, randomisé, contrôlé, en aveugle, sur l'intérêt ou non d'utiliser l'association morphine et paracétamol dans la prise en charge de la douleur modérée à sévère aux urgences.

Nous supposons que l'administration de morphine seule en intraveineux, avec une dose initiale de 0,1 mg/kg, fournira une analgésie non inférieure que la même dose de morphine associée à 1 gramme de paracétamol intraveineux chez les patients souffrant de douleur modérée à sévère pris en charge dans un service d'urgence.

Nous faisons l'hypothèse que dans le cadre de la prise en charge de patient ayant une douleur aiguë d'intensité modérée à sévère (EVN supérieure à égale à 5) aux urgences, l'administration intraveineuse de morphine seule avec une dose initiale de 0,1 mg/kg est non inférieure à l'administration de morphine avec le même schéma d'administration associée à une dose de paracétamol 1 gramme en intraveineux, à 30 minutes de la première injection du médicament à l'étude.

Quels seront les progrès apportés par l'étude dans la prise en charge de la douleur aux urgences?

Notre objectif est de fournir des preuves robustes sur la pertinence ou non de l'association systématique du paracétamol à la morphine dans la prise en charge de la douleur modérée à sévère aux urgences, à l'aide d'un essai clinique de haut niveau méthodologique.

A notre connaissance, aucune étude similaire n'a été publiée ou est en cours (PubMed, Google scholar, https://clinicaltrials.gov/).

Ainsi, notre étude fournira des données robustes et pourrait éviter aux patients douloureux aux urgences d'être exposés à la fois au paracétamol et à la morphine. Il paraît licite de limiter l'exposition des patients à des thérapeutiques inutiles et possiblement délétères pour le patient. En effet, le paracétamol, comme nous l'avons

rapporté précédemment est une molécule ayant des effets secondaires potentiellement graves. En outre, selon le rapport de l'Agence Nationale de Sécurité du Médicament de 2014, le paracétamol représentant une dépense annuelle estimée à 25 millions d'euros à l'échelle nationale. Ainsi, limiter l'utilisation du paracétamol permettra des économies de santé.

Ce projet se veut donc novateur et permettra d'établir des recommandations de haut grade, basées sur un essai clinique randomisé et multicentrique, donnée manquante dans la littérature. Notre essai aura des conséquences pratiques indiscutables et pourra aboutir à une modification de la prise en charge des patients se présentant aux urgences avec une douleur modérée à sévère.

Propositions d'amélioration et commentaires des rapporteurs de la lettre d'intention :

Lors de l'écriture du protocole détaillé de notre projet de recherche **ADAMOPA**, nous avons tenu compte des commentaires et des propositions d'amélioration des deux rapporteurs de la lettre d'intention. Nous répondons ici à leurs différentes remarques. Nous remercions les rapporteurs pour leurs rapports détaillés de la lettre d'intention du protocole **ADAMOPA**. Nos réponses à leurs commentaires sont rapportées ci-dessous.

Préselectionneur 1

1) Projet bien construit, répond à une vrai question Conséquences pratiques indiscutables Peu de doute sur la possibilité de mener à bien cette étude

Nous remercions le rapporteur pour ces commentaires positifs et de souligner l'intérêt du projet de recherche.

2) De nombreuses études ont été faites sur des questions proches mais nous n'en avons trouvé aucune qui traitait précisément de cette question. Ceci dit cette littérature est tellement abondante qu'il est difficile d'être exhaustif.

Nous remercions le rapporteur pour ce commentaire. Effectivement, la littérature est abondante dans le domaine comme nous le rapportons dans le justificatif de l'étude. Nous avons effectué une revue de la littérature (détaillée en 1.1)

qui montre qu'il manque, en effet, à ce jour un essai clinique randomisé multicentrique. Nous sommes confiants quant à l'exhaustivité de ce que nous avons rapporté et de notre recherche bibliographique. Les limites de toutes les études sont également rapportées. Essentiellement, les études sont de petite taille (sans calcul du nombre de sujets), dans des populations très différentes de celle des urgences (post-opératoires principalement), dans une autre situation (hors d'une prise en charge urgente). De plus les rares études réalisées aux urgences sont rapportées mais leurs limites méthodologiques sont très nombreuses et ne permettent pas de conclure sur la question posée, rendant indispensable la mise en place d'un essai randomisé, en double aveugle, multicentrique pour avoir une réponse claire à la question. Ainsi, notre essai ADAMOPA aura des conséquences pratiques indiscutables comme le souligne le rapporteur.

Préselectionneur 2

1) Projet qui parait intéressant et permettra d'avoir un haut niveau de preuve.
   Nous remercions le rapporteur pour ces commentaires positifs et de souligner l'intérêt du projet de recherche
2) dans les critères d'exclusion, sur le tiret "insuffisance respiratoire décompensée", supprimer la parenthèse (les patients avec ou sans ventilation artificielle seront exclus dans tous les cas!)

Nous remercions le rapporteur d'avoir relevé cette erreur typographique. Nous avons corrigé la proposition suivant la remarque du rapporteur.

3) L'analyse de la double population, douleur traumatique et douleur non traumatique, n'apparait que dans le nombre de patients à inclure et pas du tout auparavant. Il faudra corriger cela dans la rédaction du projet complet si LOI sélectionnée afin que l'on comprenne que les patients seront inclus dans l'une ou l'autre des études mais pas poolés dans la même étude

Nous remercions le rapporteur pour ce commentaire pertinent. En effet, nous avons prévu une analyse en sous-groupe (douleur traumatique et non traumatique) et le nombre de sujets nécessaires a été calculé pour que cette analyse en sous-groupe puisse se faire avec le bon nombre de sujet et la significativité statistique adaptée (modalités de l'analyse statistique détaillée en 8.2). Le nombre de sujet est

fait pour répondre aux deux analyses stratifiées. C'est pourquoi, comme le propose le rapporteur, nous avons modifié l'intitulé de l'objectif principal ainsi de clarifier notre propos et que l'on comprenne bien que les patients sont inclus et analysés suivant l'étiologie de la douleur (traumatique ou non traumatique). Nous avons de plus ajouté en objectif secondaire l'analyse combinée des 2 populations. 'Objectif principal: Evaluer la non-infériorité à 30 minutes de la morphine seule en intraveineux à l'association paracétamol et morphine en intraveineux chez les patients pris en charge aux urgences pour une douleur aiguë d'intensité modérée à sévère (EVN supérieure à égale à 5), dans un groupe de patients ayant une douleur traumatique et dans un groupe de patients ayant une douleur non traumatique.', 'Objectifs secondaires: 1/ Evaluer la non-infériorité à 30 minutes de la morphine seule en intraveineux à l'association paracétamol et morphine en intraveineux chez les patients pris en charge aux urgences pour une douleur aiguë d'intensité modérée à sévère (EVN supérieure à égale à 5), dans la population combinée des patients ayant une douleur traumatique et non traumatique.

1.2. BENEFICES ET RISQUES POUR LES PERSONNES SE PRETANT A LA RECHERCHE

1.2.1. **Bénéfices**

Bénéfice individuel

Il n'existe pas à l'heure actuelle de recommandations reposant sur des essais cliniques randomisés avec une bonne méthodologie justifiant d'associer le paracétamol à la morphine dans le cadre de la prise en charge de la douleur aiguë d'intensité modérée à sévère aux urgences. A notre connaissance et en l'état actuel des connaissances, aucune donnée ne permet d'affirmer une supériorité de cette combinaison versus morphine seule. Ainsi, les preuves sont insuffisantes pour en faire l'analgésique de référence dans cette situation, alors que c'est ce qui est classiquement appliqué dans les services d'urgence.

Le projet de recherche ADAMOPA permettra d'optimiser la prise en charge de la douleur aux urgences en ajoutant une donnée manquante à la littérature : un essai clinique randomisé et multicentrique, en double aveugle, de bon niveau méthodologique. A notre connaissance, aucun projet de recherche

comparant la morphine intraveineuse seule et la conjonction de paracétamol et de morphine intraveineuse pour l'analgésie aux urgences n'a été publié ou est en cours.

Des bénéfices individuels sont attendus pour les patients inclus dans notre étude, par une limitation de l'exposition au paracétamol et ses effets secondaires importants telle l'hypotension artérielle. En effet lors de son utilisation pour des patients hospitalisés en soins intensifs, en réinitialisant la thermorégulation des patients, l'administration du paracétamol initie un mécanisme physiologique de déperdition de chaleur, débouchant sur une baisse de la tension artérielle significative de plus de 15% par vasodilatation *(55).* Cet effet hypotenseur du paracétamol prédomine chez les patients en sepsis ou choc septique *(56)* et plus de 50% de ces hypotensions iatrogènes ont nécessité un remplissage vasculaire *(57).*

Il y aura aussi une évaluation extrêmement cadrée, protocolisée et rapprochée de la douleur, qui sous-tend intuitivement une amélioration de la prise en charge de la douleur pour le patient, dans le contexte d'une prise en charge dans un service d'urgence.

Bénéfice collectif

Des bénéfices collectifs sont aussi attendus. Le protocole ADAMOPA a pour ambition une amélioration de la prise en charge de la douleur aiguë aux urgences alors que plusieurs études ont rapportées une prise en charge de la douleur insuffisante aux urgences. Cette étude permettra, en effet, d'évaluer avec un haut niveau de preuve, l'efficacité qu'une combinaison paracétamol-morphine versus morphine seule, alors qu'il n'existe pas à l'heure actuelle de données fiables permettant d'attester de la supériorité de cette combinaison par rapport à la morphine seule. Collectivement, on peut envisager une optimisation de la prise en charge de la douleur avec notre protocole de recherche. Ainsi, les résultats de notre essai seront largement publiés et diffusés via les sociétés savantes françaises (SFMU : Société Française de Médecine d'Urgence, SFAR : Société Française d'Anesthésie-Réanimation), et vont bénéficier rapidement à la collectivité qui serait alors soustraite aux effets secondaires aigus potentiellement graves du paracétamol. Une réduction de prescription de paracétamol pourrait entraîner un bénéfice de santé publique avec une réduction de l'incidence d'insuffisance hépatique aiguë.

Enfin, un bénéfice médico-économique sociétal est également attendu. La mise en évidence d'une non-infériorité de la morphine seule par rapport à la combinaison paracétamol-morphine serait susceptible de limiter l'usage de paracétamol, réduisant de facto le coût associé à la prise en charge de la douleur aiguë aux urgences. Ainsi, l'intérêt économique est bel et bien réel, notre essai ADAMOPA permettrait de diminuer in fine le risque et les coûts associés aux prises en charge de la douleur aux urgences.

1.2.2. Risques

Risque individuel

Risques et contraintes physiques

Les stratégies thérapeutiques évaluées au cours de cet essai se basent sur l'emploi de la morphine et du paracétamol.

La prise en charge d'un patient hyperalgique via l'une des deux stratégies thérapeutiques faisant l'objet de l'étude ADAMOPA ne peut être évitée. Les deux stratégies antalgiques comparées sont actuellement utilisées dans le cadre de la pratique courante et sont principalement fonction du médecin/patient, du tableau clinique et de l'expérience du clinicien.

Le suivi réalisé durant le protocole de recherche ne s'accompagne d'aucun examen supplémentaire et n'induit pas de modifications de la prise en charge habituelle appliquée dans la gestion de la douleur aiguë aux urgences. En outre, il n'est prévu aucune journée d'hospitalisation supplémentaire, aucune prise de sang, aucune consultation et/ou déplacements supplémentaires, aucun examen complémentaire invasif ou non ou aucun questionnaire à renseigner dans le cadre de notre protocole de recherche. Hormis le recueil systématique des potentiels EI, la recherche ne présente donc pas de contraintes supplémentaires.

Ainsi, le protocole ADAMOPA ne présente pas de sur-risque ou de contrainte supplémentaire pour le patient. A contrario, le suivi du patient et l'évaluation extrêmement encadrée et rapprochée de la douleur permettra une amélioration de la prise en charge de la douleur pour le patient inclut.

Risques liés à la maladie

L'étiologie des contextes hyperalgiques modérés ou sévères aux urgences est multifactoriel. Il n'est donc pas possible de décrire la diversité et l'aggravation des tableaux cliniques éventuels rencontrés dans le protocole ADAMOPA. Il est en effet prévu d'inclure les douleurs traumatiques et non traumatiques se présentant aux urgences (cf. critères d'inclusion et de non inclusion).

Risques liés aux traitements à l'essai y compris comparateur le cas échéant (EI)

La morphine dispose d'une autorisation de mise sur le marché pour la prise en charge des douleurs intenses et/ou rebelles aux antalgiques de niveau plus faible. Il s'agit par ailleurs de l'antalgique de référence indiquée dans la prise en charge de la douleur aiguë aux urgences. Les EI sont majoritairement liés aux propriétés pharmacologiques et doses dépendants. Des complications digestives en particulier peuvent survenir.

Le paracétamol dispose également d'une autorisation de mise sur le marché pour le traitement de courte durée des douleurs d'intensité modérée. Sa toxicité en particulier hépatique s'exprime essentiellement en cas de surdosage réel ou relatif.

Les EI de ces deux médicaments sont listés dans les RCP respectifs des spécialités utilisées dans le cadre de l'AMM ou/et conformément aux recommandations d'experts.

Risques liés aux traitements et gestes/explorations associés

En fonction de la pathologie responsable de la douleur, des médicaments concomitants sont susceptibles d'être administrés au patient, et des dispositifs médicaux utilisés. En raison de la diversité des situations cliniques, il n'est pas possible d'en dresser une liste exhaustive. Ces traitements sont cependant mis en place conformément aux recommandations de bonne pratique et utilisés de la même façon que dans le soin courant. Leurs EI ou complications attendues sont listés dans les documents de référence habituels RCP ou Notice/Manuel d'utilisation en vigueur.

Risques et contraintes psychologiques

Compte tenu de l'efficacité des deux stratégies antalgiques mise en œuvre durant le protocole, le patient ne subira aucun préjudice psychologique en intégrant l'étude dans l'un ou l'autre bras du protocole. En outre, la contrainte psychologique induite par le recours à un placebo et à une randomisation est inexistante puisque les deux stratégies thérapeutiques faisant l'objet de cette étude sont déjà utilisées en pratique courante. Ainsi, par son design et son processus de recrutement, le protocole ADAMOPA est à notre sens sans sur-risques et/ou contrainte psychologique.

Risques socio-économiques

Les coûts limités et équivalents des stratégies thérapeutiques évaluées permettent de défendre l'absence de risques/contraintes socio-économiques. Au contraire, nous pouvons envisager un bénéfice collectif économique en cas de démonstration de la non-infériorité de la morphine seule versus l'association morphine et paracétamol qui permettrait de réduire l'association systématique du paracétamol à la morphine dans la prise en charge de la douleur aux urgences.

Risque collectif

Les médicaments testés ont déjà tous les deux une autorisation de mise sur le marché pour la prise en charge de la douleur aigue et sont largement utilisées en médecine d'urgence et/ou réanimation depuis de nombreuses années. Le protocole, conforme aux pratiques usuelles, n'engendrera pas de risque particulier pour la population.

1.2.3. **Balance bénéfices / risques**

En regard des contraintes et risques négligeables argumentés ci-avant, il nous semble pertinent de défendre une balance bénéfice-risque favorable, convaincante pour le patient dont l'adhésion est attendue maximisée.

Les références bibliographiques figurent en annexe du document.

2. FAISABILITE DE L'ETUDE

La douleur aigue est un motif de recours très fréquent dans les structures d'urgence. La prévalence de la douleur aiguë est évaluée à 75 % (3, 50). Pour 85% de ces patients, la douleur constitue le principal motif de recours aux services d'urgences *(58).*

Un centre comme le service des Urgences du CHU de Nantes reçoit de nombreux patients avec douleur aiguë. A titre d'exemple, 829 patients ont été pris en charge pour une douleur aiguë en 2017 (A noter que ce chiffre est sous-estimé par rapport au nombre de passage aux urgences en cette même année : 79 633. Ceci est dû à un changement de logiciel pour l'extraction des données). Ainsi, ces données démontrent la faisabilité de notre projet dans les délais proposés au vu du bassin de patients. De plus l'implication de CHU et CH assure une excellente représentativité du système et des parcours de soins, permettant d'évaluer dès à présent la reproductibilité de l'approche antalgique entre des profils de centre et d'expertise différents.

En effet, huit services d'urgences vont participer au protocole ADAMOPA (CHU de Nantes, CHU d'Angers, CHU de Tours, CHU de Brest, CHU de Bordeaux, CHU de Grenoble, CH de Châteaubriant, CH de la Roche-sur-Yon). Ils possèdent tous du personnel dédié à la recherche clinique et sont tous impliqués dans la recherche clinique en médecine d'urgence, ce qui permet de garantir le potentiel d'inclusion afin de mener à bien l'étude ADAMOPA. En effet, les équipes associées à ce projet ADAMOPA ont toutes la compétence pour mener à bien ce projet car les équipes ont déjà participé à des projets multicentriques publiés.

En outre, l'équipe à l'origine de ce projet à une solide expérience en recherche clinique dont attestent les différentes publications dans des revues nationales et internationales.

3. OBJECTIFS ET CRITERES DE JUGEMENT

3.1. OBJECTIF ET CRITERE D'EVALUATION PRINCIPAL

3.1.1. **Objectif principal**

Evaluer la non-infériorité à 30 minutes de la morphine seule en intraveineux à l'association paracétamol et morphine en intraveineux chez les patients pris en charge aux urgences pour une douleur aiguë d'intensité modérée à sévère (EVN supérieure à égale à 5), dans un groupe de patients ayant une douleur traumatique et dans un groupe de patients ayant une douleur non traumatique.

Le seuil de douleur (EVN supérieure à égale à 5) a été retenu car il est utilisé dans les études précédemment rapportées. (59, 60, 61)

Le délai d'évaluation à 30 minutes (par rapport à la première injection de paracétamol ou de morphine) a été retenu car il parait constituer un délai minimal et raisonnable permettant de stabiliser l'analgésie d'un patient. Par ailleurs, ce choix fait consensus et est aussi utilisé dans les études précédemment rapportées *(20, 62).*

3.1.2. **Critère d'évaluation principal**

Le critère de jugement principal sera la différence entre le score de l'Echelle Verbale Numérique (EVN) avant l'injection du traitement antalgique et à 30 min de la première injection du médicament à l'étude.

En effet, l'échelle verbale numérique (EVN ou échelle numérique : EN) mesure la perception de l'intensité douloureuse avec une échelle de 11 points, codée entre 0 et 10, où 0 représente l'absence de douleur et 10 représente la pire douleur imaginable. L'évaluation par l'EVN de la douleur sera faite par le médecin urgentiste en charge du patient. Il interrogera le patient pour que celui-ci donne un score à sa douleur, avant l'injection du médicament et à 30 minutes de la première injection du médicament.

Cette échelle verbale numérique (EVN) est bien corrélée avec l'échelle visuelle (EVA) analogique (63). L'EVA et l'EN ont été validés dans des études précédentes (64, 63, 65,66, 67) et il existe une grande concordance entre EVN

et l'EVA (66). L'EVN est classiquement utilisée dans la prise en charge de la douleur aiguë aux urgences et est recommandée dans les recommandations formalisées d'experts: « Il faut réévaluer l'intensité de la douleur pour apprécier l'efficacité des thérapeutiques et utiliser des échelles d'autoévaluation chez l'adulte communicant. L'échelle visuelle analogique (EVA) et l'échelle verbale numérique (EVN) sont les outils de référence » [Vivien et al. 2011]. En médecine d'urgence, ces échelles ont des taux de faisabilité de 83 à 94 % *(68, 69, 70).*

3.2. OBJECTIF ET CRITERE D'EVALUATION SECONDAIRE

3.2.1. **Objectif(s) secondaire(s)**

1/ Evaluer la non-infériorité à 30 minutes de la morphine seule en intraveineux à l'association paracétamol et morphine en intraveineux chez les patients pris en charge aux urgences pour une douleur aiguë d'intensité modérée à sévère (EVN supérieure à égale à 5), dans la population combinée des patients ayant une douleur traumatique et non traumatique.

2/ Comparer la réduction de la douleur, calculée suivant l'Échelle Verbale Numérique (EVN), à 10, 20, 30, 45 et 60 minutes de la première injection du médicament antalgique entre le groupe morphine-placebo et le groupe morphine-paracétamol.

3/ Comparer la consommation de morphine dans les deux bras durant les 30 premières minutes d'administration des produits à l'étude.

4/ Comparer le taux de succès (EVN inférieure ou égale à 3) de l'analgésie des patients à 30 minutes de la première injection de l'antalgique, dans les deux bras.

5/ Estimer et comparer la fréquence des effets indésirables dans les deux groupes de traitement à 10, 20, 30, 45 et 60 min de la première injection de l'antalgique.

6/ Comparer la nécessité d'une analgésie de secours à 30, 45 et 60 min de la première injection d'antalgique dans les deux groupes.

7/ Comparer la modification des signes vitaux à 10, 20, 30, 45 et 60 min de la première injection de l'antalgique dans les 2 groupes.

8/ Décrire la distribution de l'EVN à 10, 20, 30, 45 et à 60 min, dans les deux groupes.

3.2.2. Critère(s) d'évaluation secondaire(s)

1/ Différence entre le score de l'Echelle Verbale Numérique (EVN) avant l'injection du traitement antalgique et à 30 min de la première injection du médicament à l'étude.

2/ Évaluation de la douleur par l'EVN à 10, 20, 30, 45 et à 60 minutes de la première injection du médicament antalgique dans les deux bras.

3/ Estimation de la dose cumulée de morphine dans les deux bras en dose poids (mg/kg) durant les 30 premières minutes d'administration des traitements à l'étude.

4/ Proportion de patients ayant un score de l'échelle verbale numérique inférieur ou égale à 3 à 30 min après l'injection du médicament antalgique dans les deux bras.

5/ Fréquence et intensité des effets indésirables évalués et enregistrés par le médecin. Ces effets secondaires spécifiques incluront : nausées, vomissements, hypotension significative (pression artérielle systolique inférieure à 90 mmHg), vertiges, trouble de la vigilance, défaillance respiratoire, prurit.

6/ Nombre de prescription de molécules antalgiques de secours. Si le patient est en échec de l'analgésie (échelle verbale numérique d'évaluation de la douleur supérieure ou égale à 5) à 30, 45 ou 60 minutes, une analgésie de secours sera prodiguée par le médecin. Le choix des molécules utilisées sera laissé à la discrétion du médecin.

7/ Mesure des signes vitaux (fréquence cardiaque, fréquence respiratoire, pression artérielle, score de Glasgow) avant l'injection du médicament antalgique et ensuite à 10, 20, 30, 45 et 60 minutes.

8/ Distribution de l'EVN a 10, 20, 30, 45 et à 60 min : Paramètres de tendance centrale (moyenne et médiane), paramètres de dispersion (écart type, intervalle interquartiles et valeurs extrêmes), normalité de la distribution.

4. DESIGN DE LA RECHERCHE :

4.1. METHODOLOGIE GENERALE DE LA RECHERCHE

Il s'agit d'un essai clinique de non infériorité, prospectif, multicentrique de phase IV, contrôlé par placebo en 2 groupes parallèles, randomisé et stratifié sur le type de douleur (traumatique ou non traumatique), en double aveugle, comparant la non infériorité de la morphine en intraveineux à l'association morphine et paracétamol en intraveineux chez les patients se présentant aux urgences pour le traitement d'une douleur aigue d'intensité modérée à sévère (score de l'échelle verbale numérique ≥ 5).

4.2. SCHEMA DE L'ETUDE

La durée de recrutement de l'étude est estimée à 30 mois. Chaque patient est suivi jusqu'à la fin de sa prise en charge aux urgences.

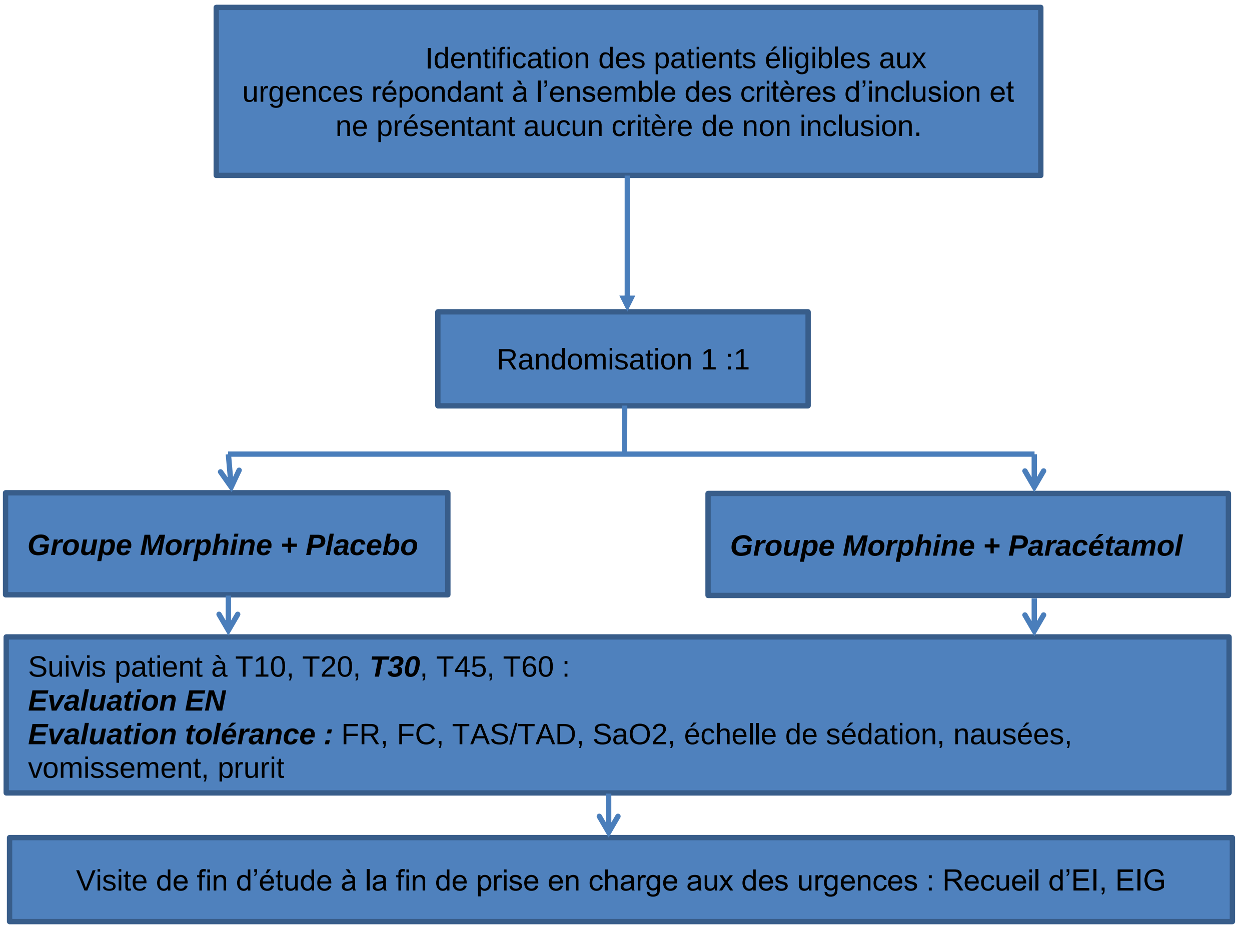

4.3. DESCRIPTION ET JUSTIFICATION DU SCHEMA THERAPEUTIQUE

La réalisation d'un essai de non infériorité, contrôlé, randomisé et en double aveugle constitue la référence méthodologique pour répondre à notre objectif : montrer la non infériorité de la morphine seule par rapport à l'association morphine et paracétamol dans la résolution de la douleur de patients pris en charge aux urgences pour douleurs aiguës d'intensité modérée à sévère (EVN≥ 5).

Les patients seront randomisés dans l'un des 2 bras de traitement pour recevoir soit de la morphine IV avec le placebo, soit la morphine IV associée au paracétamol IV.

La morphine utilisée est le chlorhydrate de morphine, qui est l'antalgique de référence recommandé en médecine d'urgence. Elle s'utilise par voie intraveineuse avec une dose de charge initiale de 0.1 mg/kg, ce qui est en accord avec les recommandations de la SFMU et de la SFAR pour les urgences intra hospitalières *(11)*. La forme intraveineuse a été préférée à celle inhalée. Les aérosols de morphine sont une alternative connue mais les informations disponibles d'efficacité et de sécurité sont insuffisantes chez l'adulte, et a fortiori aux urgences *(71)*. Les temps de nébulisation semblent le plus souvent arbitraires, avec une grande variabilité dans la cinétique de l'absorption des opiacés testés. La morphine par voie inhalée à la dose de 0,2 mg/kg ne semble pas avoir une efficacité suffisante pour une utilisation en urgence. **Ainsi, la morphine par voie intraveineuse reste la référence dans cette indication (11, 72, 73).**

Le paracétamol administré en intraveineux sera utilisé à la dose de 1 gramme. La forme intraveineuse a pour avantage une absorption plus rapide et plus prévisible que la forme orale, le délai de concentration maximale après injection intraveineuse est de 15 minutes contre une durée plus incertaine pour la prise per os, allant de 10 à 60 minutes, dû à une importante variation de la concentration plasmatique de la molécule ***(74, 75).***

4.4. DUREE DE L'ETUDE

- Durée prévue du recrutement : 30 mois.
- Durée de la participation de chaque patient: jusqu’à la fin de sa prise en charge aux urgences.
- Durée totale de l'étude: 30 mois
- Nombre de patients à recruter par mois par centre: 3

5. POPULATION ETUDIEE

5.1. DESCRIPTION DE LA POPULATION

Au total 572 patients avec les critères d'éligibilités seront recrutés dans notre étude (Cf. chapitre 8.2 Statistiques). La population cible de l'étude est constituée de patients âgés d'au moins 18 ans, pris en charge aux urgences pour une douleur aiguë d'intensité modérée ou sévère. Cette intensité de la douleur sera évaluée par l'Echelle Verbale Numérique (score supérieure ou égale à 5), et présentant une stabilité clinique. Nous excluons de notre population d'étude, les patients sous tutelle, curatelle, les femmes enceintes et allaitantes, ainsi que les mineurs.

Par ailleurs, les personnes se prêtant à cette recherche pourront participer à une autre recherche interventionnelle, une fois le management de la douleur entre la Baseline (administration des antalgiques selon le bras de randomisation) et le T60 minutes effectué. Cette fenêtre étant étendue jusqu'à la fin de la prise en charge du patient aux urgences afin de recueillir les EI/EIG en rapport avec les traitements de l'étude. Cela constitue la seule période d'exclusion de participation à une autre recherche interventionnelle.

Le recrutement des patients se fera exclusivement dans les services d'urgences participants. Chaque patient se présentant aux urgences pour douleur aiguë d'intensité modérée à sévère, quel que soit le type de douleur (traumatique ou non traumatique) sera éligible à l'étude, dès lors qu'il remplit l'ensemble des critères d'inclusion et ne présente aucun critère de non inclusion.

5.2. CRITERES D'INCLUSION

- Patient âgé d'au moins 18 ans.
- Patient rapportant une douleur évaluée par l'échelle verbale numérique supérieure ou égale à 5.
- Patient conscient (score de Glasgow = 15).
- Stabilité clinique (pression artérielle systolique >90 ou >180 mmHg, fréquence cardiaque >50 ou <150/min, et fréquence respiratoire >10 ou <30/min).
- Patient pouvant parler et donner une évaluation verbale de sa douleur avec

l'échelle verbale numérique.

- Patient majeur, hors contexte de tutelle et/ou curatelle, affilié au Régime de la Sécurité Sociale.

5.3. CRITERES DE NON-INCLUSION

- Femme enceinte ou allaitante.

La morphine utilisée en fin de grossesse peut être responsable chez le nouveau-né de différents types d'événements transitoires (syndrome de sevrage néonatal lors de prises prolongées, dépression respiratoire néonatale). Il est aussi préférable de suspendre l'allaitement pendant un traitement par morphine selon le Centre de Référence sur les Agents Tératogènes. De même nous exclurons les femmes susceptibles d'être enceintes au dire de la patiente, sans avoir recours systématiquement à un test de grossesse du fait de l'urgence de la situation.

- Patients avec un poids strictement inférieur à 50 kg.
- Patients nécessitant la réalisation d'un geste douloureux dans le cadre de la prise en charge d'une douleur traumatique (réalignement d'une fracture par exemple).
- Œdème aigu pulmonaire, insuffisance respiratoire décompensée.
- Syndrome coronarien aigu ou cardiopathie ischémique non équilibrée en cours.
- Intoxication aiguë alcoolique ou à une drogue illicite présumée.
- Patient ayant déjà bénéficié d'une administration de morphine ou de paracétamol pour l'épisode douloureux aigu en cours.
- Pas de possibilité d'avoir un accès veineux.
- Allergie connue au paracétamol ou à la morphine.
- Antécédents connus d'insuffisance rénale ou hépatique.
- Antécédents de douleurs chroniques en cours de traitement.
- Associations avec la buprénorphine, la nalbuphine et la pentazocine.

6. TRAITEMENTS UTILISES PENDANT L'ETUDE

Les patients répondant à l'ensemble des critères d'inclusion et ne présentant aucun critère de non inclusion, seront éligibles pour être randomisés à l'un des deux groupes de traitement pour recevoir soit de la morphine avec du placebo ou de la morphine avec du paracétamol.

6.1. DESCRIPTION ET MODALITES D'ADMINISTRATION

Le groupe expérimental correspond à celui recevant Morphine IV associée au placebo du paracétamol IV alors que le comparateur correspond au groupe traité par Morphine IV associée au paracétamol IV. La morphine administrée dans les deux groupes est considérée comme traitement de fond.

6.1.1. *Médicament(s) expérimental (aux) / comparateur*

Produit expérimental : PLACEBO DU PARACETAMOL IV

Le placebo du paracétamol IV consiste en du chlorure de sodium à 0,9 %. Il sera fourni par le CHU de Nantes prêt à l'emploi. Afin de maintenir le double aveugle, les poches de paracétamol 1 gramme et placebo 100mL seront masquées, étiquetées et numérotées.

Produit comparateur : PARACETAMOL IV

Description

PARACETAMOL (KABI) 10 mg/ml, solution injectable en poche 100mL

FORMES et PRÉSENTATIONS :

Solution injectable poche 100mL.

COMPOSITION :

Paracétamol 10,00 mg pour 1 ml de solution injectable.

Une poche de 100 ml contient 1000 mg de paracétamol.

Excipients : Cystéine, mannitol, eau pour préparations injectables.

Reconstitution

Il n'y a pas de préparation ou de reconstitution à réaliser avant d'administrer ce traitement. Les poches sont prêtes à l'emploi.

Administration Paracétamol IV :

Dans le groupe comparateur, le paracétamol sera administré à la dose de 1 gramme.

Adaptation posologie

Il n'y aura pas d'adaptation posologique.

Documents de référence

Le paracétamol est utilisé dans le cadre de son AMM ou des recommandations professionnelles (Cf. annexe 6 : RCP du Paracétamol).

Traitement de fond : MORPHINE IV

Description

Chaque centre investigateur administrera le chlorhydrate de morphine IV référencé dans son établissement (non fourni par le promoteur).

Reconstitution

La morphine sera diluée dans du sérum physiologique à raison de 1mg/ml dans une seringue de 10ml.

Administration

Dans les 2 groupes, l'administration de la morphine en intraveineux direct (IVD) se fera par une dose de charge initiale de 0,1 mg/kg, comme le préconisent plusieurs études déjà citées précédemment **(13, 62, 76, 77, 78)** ainsi que des manuels renommés tels que le *Tintinalli's Emergency Medicine: A Comprehensive Study Guide*. Il faut noter que les recommandations de la Société française de médecine d'urgence (SFMU) de même que celles de la SFAR pour les urgences intra-hospitalières recommandent également un bolus initial de 0,1 mg/kg ***(11).***

Adaptation posologie

Les posologies de morphine utilisées dans le cadre d'ADAMOPA font écho aux recommandations de la Société Française de Médecine d'Urgence (SFMU). La morphine sera titrée, toutes les dix minutes par bolus de 0,05 mg/kg, jusqu'à ce que le patient ait une douleur inférieure ou égale à 3, ou jusqu'à ce que le patient présente un effet indésirable sérieux : hypotension sévère, inconscience, dépression respiratoire requérant un support de ventilation.

> Recommandations formalisés d'experts de la SFMU : « Les objectifs thérapeutiques recommandés par les experts sont une EVA inférieure ou égale à 3 ou une EN inférieure ou égale à 3, avec un score de sédation Ramsay égal à 2 ou score EDS inférieur à 2 ». ***(11)***

Si le patient est en échec de l'analgésie (échelle verbale numérique d'évaluation de la douleur supérieure ou égale à 5) après 30, 45 ou 60 minutes, une analgésie de secours sera prodiguée par le médecin. Le choix des molécules utilisées sera laissé à la discrétion du médecin.

De même, en cas de signes de surdosage aux morphiniques avec apparition des symptômes suivants : somnolence (signe d'appel précoce de l'apparition d'une décompensation respiratoire, myosis extrême, hypotension), la morphine sera arrêtée. Une prise en charge adaptée sera engagée, avec stimulation-ventilation assistée, avant réanimation cardio-respiratoire en service spécialisé et traitement spécifique de l'intoxication aux morphiniques par la Naloxone (Cf. traitement auxiliaires et traitement d'urgence) avec surveillance pendant le temps nécessaire jusqu'à disparition des symptômes.

Documents de référence

La morphine est utilisée dans le cadre de son AMM ou des recommandations professionnelles (Cf. RCP en vigueur de la spécialité, un modèle est joint en Annexe 8).

6.1.2. *Médicaments auxiliaires*

Le protocole prévoit conformément aux pratiques des urgentistes, l'utilisation de la Naloxone dans le cadre d'un surdosage dans le groupe morphine. De même, la survenue de nausées ou de vomissements pourra nécessiter la prescription de métoclopramide. De plus, si l'analgésie n'est pas efficace, le médecin peut prescrire une autre molécule antalgique de son choix.

NALOXONE 0,4 mg/1 ml, solution injectable en ampoule

Description

FORME ET PRESENTATIONS:

Solution injectable.

COMPOSITION :

Chlorhydrate de Naloxone anhydre 0.4 mg pour 1 ampoule de 1ml.

Une ampoule de 1 ml contient 10 mg de chlorhydrate de morphine

Excipients : Chlorure de sodium, acide chlorhydrique concentré, eau pour préparations injectables.

Administration

La Naloxone est injectée par voie intraveineuse, des réinjections sont pratiquées si nécessaire jusqu'à normalisation des paramètres.

Une dose initiale de 0,4 à 2 mg de Naloxone peut être administrée par voie I.V. L'administration se fera par doses progressives de 0,1 mg jusqu'à l'obtention d'une ventilation respiratoire suffisante. Si l'amélioration clinique est jugée insuffisante, on renouvellera la dose initiale à des intervalles de 2 à 3 minutes.

Adaptation posologie

L'administration se fera par doses progressives de 0,1 mg jusqu'à l'obtention d'une ventilation respiratoire suffisante. Si l'amélioration clinique est jugée insuffisante, on renouvellera la dose initiale à des intervalles de 2 à 3 minutes.

CHLORHYDRATE DE METOCLOPRAMIDE 10 mg/2 mL, solution injectable en ampoule

Description

FORME ET PRESENTATIONS :

Solution injectable.

COMPOSITION :

Chlorhydrate de métoclopramide 10,50 mg (Quantité correspondante en chlorhydrate de métoclopramide anhydre 10,00 mg) Pour une ampoule de 2 mL.

Excipients : Chlorure de sodium, eau pour préparations injectables.

Administration

Pour le métoclopramide se reporter au RCP de la spécialité en vigueur.

Adaptation de la posologie

Non applicable

6.2. ALLOCATION DES TRAITEMENTS ET MISE EN INSU

6.2.1. *Allocation des traitements : procédures de randomisation*

Les sujets seront assignés au hasard à l'un des 2 bras de traitement dans un rapport 1:1 basé sur une liste de randomisation générée par ordinateur et préparé avant l'étude par le biostatisticien. Les produits à l'étude sont conditionnés en conformité avec cette liste. Les investigateurs n'auront pas accès à la liste randomisée des numéros de traitements.

La randomisation sera équilibrée en utilisant des blocs de permutations aléatoires et sera stratifiée sur le type de douleur (Traumatique/Non Traumatique). Elle se fera via le logiciel Clinsight en se connectant sur le site https://nantes-lrsy.hugo-online.fr/CSOnline/ avec un identifiant et un mot de passe émis par un responsable du département Promotion de la recherche du CHU de Nantes. La plateforme Clinsight attribuera un code de traitement unique, qui déterminera l'affectation du traitement et la poche de médicament correspondante pour le sujet. Un email de confirmation en aveugle sera envoyé à toutes les personnes impliquées dans cette étude.

Un patient randomisé est un patient qui a été placé dans un groupe de traitement randomisé, que le traitement ait été administré ou non. A noter qu'un patient ne peut pas être randomisé plus d'une fois dans l'étude.

6.2.2. *Méthode de mise en insu*

En raison des objectifs de l'étude, un design en double aveugle a été mis en place, le médecin et le patient ignorent tous les deux la nature du traitement administré. Afin de maitriser cet insu, les traitements sont indiscernables. Pour cela les poches de paracétamol 1 gramme et de son placebo seront masquées et étiquetées par la PUI, selon les procédures de fabrication en vigueur. Il sera ainsi impossible de reconnaître le comparateur (Paracétamol) et le placebo correspondant, ni par l'équipe médicale ou paramédicale, ni par le personnel de recherche, ni par le patient.

Seule la morphine sera le traitement connu car d'une part, il est non éthique d'administrer du placebo à la place de la morphine du fait de l'obligation de soulager le patient. D'autre part, la morphine possède des effets secondaires facilement reconnaissables par l'équipe médicale et pour lesquelles il peut y avoir besoin de prescrire un antidote ou d'un traitement correcteur, essentiellement en cas de surdosage. De même, dans le cadre des bonnes pratiques de soins, il est recommandé que l'infirmière fasse une double vérification du nom du produit et de la dose de médicament avant de l'injecter au patient pour assurer la sécurité de celui-ci *[HAS, guide_outils_securisation_autoevaluation_administration_medicaments]*.

6.3. MEDICAMENTS ET TRAITEMENTS AUTORISES ET INTERDITS

6.3.1. *Traitements autorisés*

ADAMOPA s'inscrivant dans la pratique courante de la Médecine d'Urgence, aucun traitement justifié par l'état clinique n'est exclu.

6.3.2. *Traitements non autorisés*

Aucun traitement n'est interdit dans le protocole, sauf l'ajout de morphine et/ou de paracétamol en plus des médicaments administrés selon le schéma protocolaire et ce jusqu'à 30 minutes après administration de celui-ci (horaire d'évaluation du critère principal).

6.3.3. *Traitement d'urgence*

Le protocole prévoit conformément aux pratiques des urgentistes, l'utilisation de la Naloxone (Cf. description chapitre 5.1.3.1 Médicaments auxiliaires) dans le cadre d'un surdosage de Morphine.

De même, la survenue de nausées ou de vomissements pourra nécessiter la prescription de métoclopramide (Cf. description chapitre 5.1.3.1 Médicaments auxiliaires).

Par ailleurs, comme décrit plus haut, si l'analgésie n'est pas efficace (EVN ≥ à 5 à 30 minutes du début de l'injection de l'antalgique de l'étude), le médecin peut prescrire un autre médicament antalgique de son choix. Cette possibilité de prescrire une analgésie de secours ne sera possible qu'à partir de la 30ème minute, moment où est évalué le critère de jugement principal.

6.4. METHODES DE SUIVI DE L'OBSERVANCE AU TRAITEMENT

L’utilisation du paracétamol/placebo seront tracés à chaque utilisation, en reportant le numéro présent sur la poche ou en collant l’étiquette.

En pratique, après chaque utilisation, l’ensemble des poches vides et entamées seront gardées, décomptées extemporanément par l’équipe de recherche clinique et renvoyées à la pharmacie, accompagnées du document de traçabilité.

La traçabilité de la morphine suit le schéma habituel de traçabilité des opioïdes dans les soins. Il n’y aura pas de traçabilité spécifique dans le cadre de cette recherche.

6.5. CIRCUIT DES MEDICAMENTS EXPERIMENTAUX

6.5.1. *Circuit général*

La Pharmacie du CHU de Nantes sera en charge d’acheter auprès des fournisseurs du paracétamol et de son placebo présentés sous forme de poche (voie IV). Elle procèdera à leur masquage et à leur étiquetage dans un but de mise en aveugle. Les UT étiquetées et numérotées seront envoyés aux pharmacies hospitalières des centres investigateurs puis mise en dotation dans le service du fait de l’urgence à traiter.

6.5.2. *Conditions de stockage des médicaments expérimentaux :*

Description du stockage à la pharmacie

Les UT de paracétamol et son placebo seront gérées par la pharmacie du CHU de Nantes en fonction du rythme des inclusions chaque centre (email de randomisation automatique transmis par l’interface de Capture System renseignée par les équipes de recherche clinique à chaque inclusion.

Les pharmaciens de chaque centre participant sont chargés du stockage des unités thérapeutiques à température ambiante dans un local à accès restreint.

Description du stockage dans le service

Dans la mesure où la mise en route du traitement antalgique est une urgence, une dotation sera mise en place dans le service des urgences. Les UT de paracétamol et son placebo seront stockées sur le site de l'étude au sein du service des urgences à une température ambiante dans un espace séparé des médicaments su soin courant.

6.5.3. *Procédure de levée d'aveugle :*

En cas d'Evénement Indésirable (EI) ou d'Evènement Indésirable Grave (EIG), la levée d'aveugle ne devra avoir lieu que dans les cas où il est nécessaire de connaître le produit à l'étude pour traiter le patient.

En cas d'EIGI, la levée d'aveugle est effectuée pour la transmission de l'EIGI à l'autorité de santé.

7. DEROULEMENT DE L'ETUDE

7.1. CALENDRIER DE L'ETUDE

7.1.1. *V0 : Sélection des patients et inclusion :*

Lors de la visite de sélection, les critères de sélection à l'étude seront vérifiés. Les patients qui répondent avec succès à tous les critères d'inclusion et n'ont aucun des critères de non inclusion seront éligibles pour participer à l'étude. Le consentement sera ensuite recueilli par l'investigateur après entretien et la délivrance de la lettre d'information.

Une fois le patient inclus, un numéro d'identification du patient est obtenu en se connectant, avec les identifiants personnels de chaque personne habilitée, au site internet Clinsight et les éléments suivants seront consignés :

- Critères d'inclusion et de non inclusion.
- Données démographiques: Age, sexe.
- Antécédents médicaux : angor ou ischémie myocardique, bronchopneumopathie obstructive, insuffisance rénale, insuffisance hépatique, antécédents de prescription d'opioïdes et si oui le nom du médicament, la date ainsi que la dose seront précises, antécédents d'antalgiques déjà reçu pour l'épisode douloureux en cours et si oui, le nom du médicament et la dose seront précisés.
- L'examen physique incluant : taille, poids, signes vitaux (fréquence cardiaque, fréquence respiratoire, pression artérielle, score de Glasgow).

7.1.2. *V1 : Randomisation des patients*

Les sujets qui répondent à tous les critères d'éligibilité seront randomisée selon un ratio 1:1 pour recevoir un des 2 traitements suivants :

- Traitement Bras 1: Morphine + Placebo.
- Traitement Bras 2 : Morphine + Paracetamol

La randomisation sera centralisée par internet via l'interface Web Clinsight avec une stratification sur le type douleurs : Traumatiques/Non Traumatiques. Les kits de randomisation incluront soit le paracétamol ou son placebo.

A la suite de la randomisation du patient, les éléments suivants seront complétés :

- Numéro de traitement attribué par randomisation.
- Le délai entre l'entrée du patient aux urgences et sa randomisation.
- Evaluation de la douleur avant l'injection du médicament par l'EVN.
- Type de la douleur:
    - Non traumatique :
        - Thoracique.
        - Douleur abdominale.
        - Céphalées.
        - Dos ou musculosquelettique.
        - Autres, précisez :
    - Traumatique :
        - Tête et cou.
        - Face.
        - Thorax.
        - Abdomen, pelvis.
        - Bassin, membres.
        - Peau, tissu sous cutané.
        - *NB: Pour chaque localisation de la lésion traumatique seront précisées la sévérité de la lésion qui se décline en 6 stades: mineure, modérée, sérieuse, sévère, critique et maximale et ce afin de calculer le Trauma Injury Severity Score (Score ISS).*
- Heure d'injection des médicaments à l'étude (T0) et la dose administrée.

7.1.3. *V2 : Suivi des patients à T10, T20, T30, T45 et T60*

Chaque patient sera suivi au temps 10 min, 20 min, 30 min, 45 min et 60 min du début de l'injection du traitement antalgique et ce afin d'évaluer la douleur et la tolérance.

Durant cette période de suivi, les données suivantes seront colligées :

- Evaluation de la douleur par l'EVN,
- Paramètres vitaux :
  - Fréquence cardiaque,
  - Fréquence respiratoire,
  - Pression artérielle,
  - Saturométrie pulsée
  - Score de Glasgow
- Dose cumulée de morphine dans les deux bras en dose poids (mg/kg),
- Evaluation des effets indésirables :
  - Sédation par le calcul du score de Ramsay :
    - <u>Niveau Réponse 1 :</u> Le malade est anxieux, agité
    - <u>Niveau Réponse 2 :</u> Le malade est coopérant, orienté et calme
    - <u>Niveau Réponse 3 :</u> Le malade est capable de répondre aux ordres
    - <u>Niveau Réponse 4 </u>: Le malade est endormi, mais peut répondre nettement à la stimulation de la glabelle ou à un bruit intense
    - <u>Niveau Réponse 5 :</u> Le malade est endormi, et répond faiblement aux stimulations verbales
    - <u>Niveau Réponse 6 :</u> Le malade ne répond pas aux stimulations nociceptives

  - Autres effets indésirables dont:
    - Signes neurosensoriels : Vertiges, malaise, troubles de la vigilance (autres que sédation).
    - Signes digestives dont Nausée, vomissements,

- hypotension significative (pression artérielle systolique inférieure à 90 mmHg),
- Signes respiratoires : Défaillance respiratoire,
- Signes cutanéomuqueux : Prurit et éruption.

- Prescription de Naloxone, d'un antalgique ou d'une autre molécule pouvant interagir avec les médicaments à l'étude et si oui, le nom de la molécule et la dose prescrite seront précisés.

Le temps d'évaluation à 30 min de la première injection du médicament devra être obligatoirement réalisé car il constitue l'élément clé dans l'évaluation du critère de jugement principal de l'étude.

Si le patient est en échec de l'analgésie (échelle verbale numérique d'évaluation de la douleur supérieure ou égale à 5) après 30, 45 ou 60 minutes, une analgésie de secours sera prodiguée par le médecin. Le choix des molécules utilisées sera laissé à la discrétion du médecin et le nom de ces molécules ainsi que leur dose seront consignés dans l'eCRF.

7.1.4. *V3 : Visite de fin d'étude à la fin de prise de la prise en charge aux urgences*

Cette visite aura lieu dès la décision médicale de fin de prise en charge du patient aux urgences et ce afin de relever d'éventuels effets indésirables durant la période d'exposition au traitement.

Lors de cette visite, le recueil de tous les EI et EIG en rapport avec l'utilisation des produits à l'étude sera vérifié.

CALENDRIER DE L'ETUDE

**Actions**	Inclusion	T0 : Injection de l'antalgique	T10	T20	T30	T45	T60	Fin de prise en charge aux urgences
Information patient	X							
Consentement éclairé	X							
Randomisation	X							
Antécédents	X							
Examen clinique	X		X	X	X	X	X	
Evaluation de la douleur (EVN)	X		X	X	X	X	X	
Paramètres vitaux (TA, FC, FR, Score de Glasgow)	X		X	X	X	X	X	
Score de Ramsay			X	X	X	X	X	
Analgésie de secours					SB*	SB	SB	
Dispensation des Traitements		X	SB	SB	SB	SB	SB	
Evénements indésirables		X	X	X	X	X	X	X

* *SB : Si Besoin. Pour le patient en échec de l'analgésie (score de l'échelle verbale numérique supérieure ou égale à 5) après 30, 45 ou 60 minutes, une analgésie de secours sera prodiguée par le médecin. Le choix des molécules utilisées sera laissé à la discrétion du médecin.*

7.2. IDENTIFICATION DE TOUTES LES DONNEES SOURCES NE FIGURANT PAS DANS LE DOSSIER MEDICAL

Toutes les données sources (Cf. calendrier de l'étude) qui seront recueillies directement dans le cahier d'observation électronique figureront dans le dossier médical du patient. Hormis les mesures répétées et rapprochées de l'EVN et des paramètres vitaux à 10, 20, 30, 45 et 60 min après injection des traitements à l'étude, pour lesquels un carnet de recueil sera constitué et ce afin de pouvoir répondre à notre objectif primaire et nos objectifs secondaires.

7.3. REGLES D'ARRET DE LA PARTICIPATION D'UNE PERSONNE

7.3.1. *Critères d'arrêt prématuré de la participation d'une personne à la recherche*

Une fois qu'un patient est randomisé dans l'étude, tout sera mis en œuvre dans la mesure du possible pour le suivre pendant toute la durée de l'étude, même s'il y a eu une déviation au protocole, un arrêt prématuré des traitements à l'étude ou des visites de suivi.

Cependant, des critères d'arrêt prématurés de participation d'un patient à la recherche sont définis comme suit :

- Patient demandant de sortir de l'étude à n'importe quel moment et quelle que soit la raison.
- Patients pour qui une évaluation rigoureuse du critère de jugement principal à 30 minutes de l'injection du médicament, n'a pu être effectuée.

L'exclusion du patient est obligatoire dans ces cas, et les données le concernant ne seront pas utilisées pour la recherche.

De même l'investigateur pourra interrompre temporairement ou définitivement la participation d'un sujet à l'étude pour toute raison qui servirait au mieux les intérêts du sujet en particulier en cas d'événements indésirables graves ou de faits nouveau vis-à-vis de la recherche.

Concernant les sujets perdus de vu, étant donné le temps de suivi très court dans cette étude, nous estimons qu'il n'y aura pas de perdu de vue. Si tout de même cela se produit, l'investigateur mettra tout en œuvre pour reprendre contact avec la personne. Les données recueillies pour les sujets perdus de vue ou sortis prématurément de l'étude seront exploitées au moment des analyses sauf en cas de retrait de consentement.

7.3.2. *Procédures d'arrêt prématuré de la participation d'une personne à la recherche*

En cas de sortie prématurée, l'investigateur doit en documenter les raisons de façon aussi complètes que possible dans le dossier source ainsi que dans l'eCRF.

Le suivi d'un patient exclu sera le même que pour un patient inclus. La sortie d'étude d'un patient ne changera en rien sa prise en charge habituelle par rapport à sa maladie. En cas d'évènement indésirable, grave ou non, un suivi adapté sera effectué.

7.3.3. *Critères d'arrêt d'une partie ou de la totalité de la recherche (hors considérations biostatistiques)*

La fin de la recherche correspond à la fin de la participation du dernier sujet inclus.

Une partie ou la totalité de l'étude peut être arrêtée définitivement ou temporairement sur décision de l'ANSM, du CPP. De même le CHU de Nantes se réservera le droit d'interrompre l'étude, s'il s'avérait que les objectifs d'inclusion n'étaient pas atteints.

Dans tous les cas :

Une confirmation écrite sera envoyée à l'investigateur coordonnateur de l'étude (précisant les raisons d'arrêt prématuré) ainsi qu'à l'investigateur principal de chaque centre le cas échéant.

Tous les patients de l'étude seront informés et devront réaliser leur visite de sortie prématurée. Tous les patients inclus dans l'étude doivent être informés et devraient assister à leur visite de sevrage précoce.

7.4. MODALITE DE PRISE EN CHARGE DES PATIENTS A LA FIN DE LA RECHERCHE

Il n’y a pas de modalité particulière de prise en charge des patients à la fin de la recherche, après la fin de leur prise en charge aux urgences et la surveillance de pharmacovigilance.

7.5. INDEMNISATION

Non applicable.

8. DATA MANAGEMENT ET STATISTIQUES

8.1. RECUEIL ET TRAITEMENT DES DONNEES DE L'ETUDE

8.1.1. *Recueil, traitement et circulation des données*

Le recueil des données de chaque personne se prêtant à la recherche est réalisé par l'intermédiaire d'un cahier d'observation électronique (eCRF). Toutes les informations requises par le protocole ainsi que les données nécessaires aux analyses statistiques doivent être fournies dans l'eCRF. Les données seront recueillies au fur et à mesure qu'elles sont obtenues. Des commentaires pourront être ajoutés pour justifier ou expliquer les données manquantes ou les valeurs en dehors des normes attendues. De même, l'eCRF doit inclure les données nécessaires pour confirmer le respect du protocole et identifier les écarts majeurs.

Les personnes responsables de remplir le formulaire eCRF (investigateur, TEC, ARC de monitoring…) doivent être définies et identifiées dans le tableau de délégation de responsabilités de chaque centre (conservé dans le dossier de l'investigateur). Chaque personne responsable de ce recueil aura un compté « utilisateur » avec les droits informatiques spécifiques à son rôle (droit de saisir ou modifier une donnée, droit de verrouiller, monitorer ou signer une page eCRF).

La saisie, la consultation ou la modification des données ne sera possible que via les pages de l'eCRF (masques de saisie), sur le site internet https://nantes-lrsy.hugo-online.fr/CSonline.

Le data manager en charge de l'essai assurera une assistance en cas de problème lié à l'utilisation de l'eCRF. A noter que le Data Manager ne peut ni effacer ni modifier les données enregistrées dans la piste d'audit.

8.1.2. *Identification du participant*

En signant le protocole, l'investigateur principal et l'ensemble des Co-investigateurs s'engagent à maintenir confidentielles les identités des personnes se prêtant à la recherche en leur attribuant un code.

Ce code est utilisé pour tous les eCRF et tous les documents (compte-rendu d'examens d'imagerie, de biologie, …) joints. C'est la seule information qui permet à postériori de faire la correspondance avec le participant.

Selon les recommandations de la CNIL, la première lettre du nom, la première lettre du prénom et la date de naissance (seulement mois et année de naissance), seront les seules informations qui figureront sur le cahier d'observation et qui permettront de rattacher à postériori l eCRF au patient. A cela on rajoutera le numéro d'inclusion selon le modèle (1er lettre du Nom + 1er lettre du prénom + N° de randomisation à 3 chiffres).

L'investigateur est également tenu de coder les données patientes sur tous les documents qu'il pourrait avoir en sa possession (compte-rendu d'examens d'imagerie, de biologie…) qui seraient joints à l eCRF. De même, la présentation des résultats de la recherche doit exclure toute identification directe ou indirecte.

8.2. STATISTIQUES

L'analyse statistique sera réalisée à l'aide du logiciel Stata ou SAS au sein de la plateforme de Biométrie du CHU de Nantes sous la responsabilité de Jean-Benoît Hardouin (Plateforme de Méthodologie et de Biostatistiques-DRCI-CHU de Nantes – jeanbenoit.hardouin@univ-nantes.fr).

8.2.1. *Description des méthodes statistiques prévues, y compris du calendrier des analyses intermédiaires prévues*

L'analyse statistique sera réalisée à la fin de l'étude. Il n'est pas prévu d'analyse intermédiaire.

Le diagramme de sélection des participants à l'étude sera réalisé ainsi qu'une analyse descriptive des caractéristiques de chaque groupe. Les variables qualitatives seront présentées avec les effectifs et pourcentages de chaque modalité. Les variables quantitatives seront décrites par la moyenne, la médiane, l'écart-type, les quartiles, le minimum et le maximum.

Analyse du critère principal

L'analyse du critère de jugement principal sera conduite en 2 temps : per protocole avec une hypothèse de non-infériorité du groupe intervention puis en intention de traiter. Cette double approche correspond aux préconisations d'analyse des essais de non-infériorité (D'agostino RB, Massaro JM, Sullivan LM. Non-Inferiority Trials).

La non infériorité du groupe morphine seule sera définie si les deux bornes inférieures des intervalles de confiance unilatéral à 97.5% de la variation de score mesuré par l'EVN entre l'inclusion et 30 minutes dans chaque des deux groupes (douleurs traumatiques et non traumatiques) seront supérieures aux deux moyennes du même critère dans les deux mêmes groupes pour les patients ayant reçu l'association morphine + paracétamol auquel nous soustrairons la marge de non infériorité fixée par les experts à 1 point.

Analyse des critères secondaires

Les critères secondaires seront analysés en per-protocole et en intention de traiter en utilisant des tests bilatéraux.

Pour chaque critère de jugement secondaire quantitatif ou qualitatif, nous estimerons dans chaque groupe de traitement, les paramètres de tendance centrale (moyenne et médiane) et de dispersion (écart-type, intervalle interquartiles, valeurs maximale et minimale). Ces critères seront comparés avec des tests de Student (critères quantitatifs) ou des tests du chi-deux (critères qualitatifs).

Analyse en sous-groupe

L'analyse du sous-groupe de patients inclus dans douleurs traumatiques et ceux du sous-groupe douleurs non traumatiques seront réalisés en per-protocole et en intention de traiter. Elles seront réalisées sur le critère de jugement principal (différence EVN à 30 minutes) et sur les autres critères secondaires avec des tests bilatéraux.

8.2.2. *Justification statistique du nombre d'inclusions*

Dans notre étude 572 patients seront nécessaires afin de répondre à notre objectif principal.

L'objectif principal de l'essai est de démontrer la non-infériorité de la morphine seule versus morphine associée au paracétamol par rapport au critère principal d'évaluation, à savoir la différence de l'Echelle Verbale Numérique entre la baseline et 30 min de l'injection des médicaments antalgiques, dans un groupe de patients ayant une douleur traumatique et dans un groupe de patients ayant une douleur non traumatique. Nous faisons l'hypothèse que l'utilisation de la morphine seule permettrait de soulager les patients avec autant d'efficacité que l'association morphine-paracétamol.

Afin de réaliser cette analyse de non-infériorité dans chacun des 2 sous-groupes de patients (douleur traumatique et douleur non-traumatique), une marge de non-infériorité définie à 1, un écart-type à 2.6, un risque alpha ajusté à 2.5% (5%/2) et une puissance à 90%, il faudra 143 patients dans chacun des 4 groupes : 2 sous-groupes et 2 bras de traitements comparés, soit 572 patients au total.

8.2.3. *Degré de signification statistique prévu*

Le degré de signification statistique prévu est de 5%, p = 0,05.

8.2.4. *Critères statistiques d'arrêt de la recherche*

Il n'y aura pas d'analyse intermédiaire et aucun critère statistique d'arrêt de la recherche.

8.2.5. **Méthode de prise en compte des données manquantes, inutilisées ou non valides**

Pour les patients pour lesquels le critère principal ne pourra pas être évalu**é** (décès, pas de possibilité de réévaluer le patient à 30 min…), une imputation par la méthode du « pire cas » sera réalisée. A ce titre, la plus petite différence observée sur les patients du même groupe (douleurs traumatiques/non traumatiques) et ayant reçu le même traitement (morphine seule ou morphine + paracétamol) sera imputée.

Une analyse de sensibilité en imputant pas la moyenne du groupe sera aussi réalisée. Par définition, cette imputation n'impactera que les analyses en intention de traiter.

8.2.6. Gestion des modifications apportées au plan d'analyse de la stratégie initiale

Pas de modification prévue au plan d'analyse de la stratégie initiale.

8.2.7. *Choix des personnes à inclure dans les analyses*

S'agissant d'un essai de non-infériorité, l'analyse se fera dans un premier temps en per-protocole et concernera seulement les patients qui n'ont présentés ni changement ou arrêt de traitement, ni infraction au protocole et pour lesquels le critère d'évaluation principal à 30min est disponible. Elle sera complétée dans un second temps par une analyse en intention de traiter où les données de tous les patients inclus seront utilisées en respectant le groupe d'appartenance à l'inclusion.

9. PHARMACOVIGILANCE ET GESTION DES EVENEMENTS INDESIRABLES

9.1. DEFINITIONS

Vigilance	C'est la surveillance des médicaments, dispositifs médicaux et autres produits de santé. Elle consiste également à la prévention du risque d'effet indésirable résultant de leur utilisation, que ce risque soit potentiel ou avéré
Evénements indésirables (EvI)	Toute manifestation nocive survenant chez une personne qui se prête à une recherche impliquant la personne humaine que cette manifestation soit liée ou non à la recherche ou au produit sur lequel porte cette recherche.
Intensité des Evénements indésirables (EvI)	*1 = bénin*<br>*2 = modéré*<br>*3 = sévère*<br>*4 = mettant en jeu le pronostic vital*<br>*5 = décès*<br>Score de Ramsay pour la sédation
Effets Indésirables (EI)	Evénement indésirable survenant chez une personne qui se prête à une recherche impliquant la personne humaine, lorsque cet événement est lié à la recherche ou au produit sur lequel porte cette recherche.
Effet indésirable d'un médicament expérimental	Toute réaction nocive et non désirée à un médicament expérimental quelle que soit la dose administrée. (également applicable aux produits de thérapie cellulaire)
Effets/ Evènements indésirables graves (EIG)/ (EvIG)	Tout effet / évènement indésirable qui :<br>* entraîne le décès,<br>* met en jeu le pronostic vital,<br>* entraîne une incapacité ou une invalidité temporaire ou définitive,<br>* nécessite ou prolonge une hospitalisation du patient,<br>* entraîne une anomalie congénitale ou néonatale,<br>* est médicalement important (la liste des effets/ évènements médicalement important est définie par l'EMA).
Effets indésirables inattendus (EII)	Tout effet indésirable dont la nature, la sévérité ou l'évolution ne concorde pas avec les informations relatives aux produits, actes pratiqués et méthodes utilisées au cours de la recherche.
Fait nouveau	Toute nouvelle donnée pouvant conduire à une réévaluation du rapport des bénéfices et des risques de la recherche ou du produit objet de la recherche, à des modifications dans l'utilisation de ce produit, dans la conduite

	de la recherche, ou des documents relatifs à la recherche, ou à suspendre ou interrompre ou modifier le protocole de la recherche ou des recherches similaires. Pour les essais portant sur la première administration ou utilisation d'un produit de santé chez des personnes qui ne présentent aucune affection : tout effet indésirable grave.
Abus	Utilisation excessive intentionnelle, persistante ou sporadique d'un médicament qui est accompagnée par des réactions physiques ou psychologiques nocives.
Surdosage	Administration d'une quantité de médicament, donnée lors d'une administration ou de manière cumulative, qui est au-dessus de la dose maximale recommandée selon les règles de conformité ou d'utilisation du produit. Un jugement clinique devra toujours être appliqué.<br>(surdosage réel : dû à une quantité brute trop importante /surdosage relatif : dû aux facteurs prédisposants du patient tel que insuffisance rénale, hypo-albuminémie…)
Mésusage ou utilisation hors AMM	Situation où le produit est intentionnellement utilisé de manière non conforme aux spécifications d'utilisation du produit (ex : voie d'administration/posologie ou indication différentes que celle listés dans le document de référence).
Défaut qualité	Non-conformité aux spécifications décrites dans le dossier d'AMM/ marquage CE/documentation technique ou une déviation par rapport aux bonnes pratiques de fabrication (BPF) / aux bonnes pratiques de distribution, de conservation, d'étiquetage.
Erreur médicamenteuse (EM)	Correspond à toute omission ou réalisation non intentionnelle, avérée (ou potentielle), d'un acte survenu au cours du processus de soins, *dans le circuit (de la fabrication à l'administration)* impliquant un produit qui peut être à l'origine d'un risque ou d'un événement indésirable pour le patient. Le risque d'erreur ou l'erreur potentielle, concerne les situations où l'erreur ne s'est pas produite, a été interceptée mais aurait pu survenir.

9.2. PARAMETRES D'EVALUATION DE LA SECURITE

9.2.1. Critères d'évaluation particuliers liés à la sécurité

La période d'observation de sécurité s'étendra du moment où le patient est inclus dans l'étude jusqu'à la fin de la fin de sa prise en charge aux urgences.

Les effets indésirables attends des traitements protocolaires seront monitorées en termes d'occurrence et d'intensité sur l'ensemble de la fenêtre thérapeutique, étendue jusqu'à la fin de la prise en charge du patient aux urgences. Les paramètres d'évaluation sont représentés essentiellement par l'EVN et la surveillance intègre le suivi clinique (conscience, signes digestifs, cutanées), les paramètres vitaux (FR, FC, PAS/PAD) qui seront monitorés, toutes les 10 min durant

les 30 premières minutes, puis toutes les 15 minutes durant les 30 minutes suivantes, jusqu'à T60min. Puis à H6.

9.2.2. Méthodes et calendrier prévus pour mesurer, recueillir et analyser les paramètres d'évaluation de la sécurité

La sécurité sera évaluée au décours de la fenêtre thérapeutique d'utilisation de la morphine et du paracétamol. Cette surveillance est étendue jusqu'à la fin de la pris en charge du patient aux urgences.

9.3. LISTE DES EI ATTENDUS

Dans le cadre du présent protocole, les EI attendus sont :

9.3.1. Concernant le traitement à l'étude : le comparateur

Les effets indésirables attendus des médicaments protocolaires sont listés au paragraphe 4.8 du RCP considéré comme les documents de référence applicables pour la détermination du caractère attendu/inattendu.

Les effets indésirables les plus classiquement décrits sont listés ci-dessous :

- Concernant le placebo de paracétamol (NaCl 0.9%) :

Les EI en lien avec l'administration du NaCL sont peu fréquent, le principal risque correspond à la survenue de variations de ioniques, dont hyper natrémie et aux signes cliniques associés, au demeurant peu probables dans le cadre de l'administration unique, en dehors de situation d'erreur.

Les EI liés au NaCL figurent dans le RCP.

- Concernant le paracétamol
  - Le principal risque est celui d'un surdosage réel ou relatif, à l'origine d'une toxicité principalement hépatique*
  - Les réactions secondaires sont rares (> 1/10 000, < 1/1000) ou très rares (< 1/10 000), elles sont décrites ci-dessous :

Système	Rares (> 1/10 000, < 1/1000)	Très rares (< 1/10 000),
Général	Malaise	Réaction d'hypersensibilité
Cardiovasculaire	Hypotension	
Hépatique	Elévation des transaminases hépatiques	
Plaquette/sang		Thrombocytopénie<br>Leucopénie<br>Neutropénie

- De très rares cas de réactions d'hypersensibilité allant du simple rash cutané ou urticaire au choc anaphylactique ont été rapportés et nécessitent l'arrêt du traitement.
- Des cas d'érythème, de bouffées vasomotrices, de prurit et de tachycardie ont été rapportés.

➢ Pour la morphine, les effets indésirables sont majoritairement liés aux propriétés pharmacologiques et doses dépendantes, les principaux EI attendus décrits sont :

  - Des troubles digestifs dont nausées, vomissements, constipation, sécheresse buccale…
  - Des signes respiratoires avec dyspnée et dépression respiratoire,
  - Hypertension Intracrânienne.
  - Des troubles neurosensoriels, avec somnolence, confusion, mais aussi excitation, hallucination, cauchemars et parfois myoclonies en particulier en cas de fortes doses administrées rapidement, un myosis aréactif à la lumière.
  - Des troubles urinaires avec dysurie, rétention d'urine, insuffisance rénale.
  - De plus, des réactions de type anaphylactoïdes, exceptionnellement anaphylactiques, avec prurit, rash, œdème,

bouffées vasomotrices peuvent être observées. Des formes sévères (choc) peuvent survenir, une évolution fatale ne pouvant être exclue.

- Des syndromes de sevrage peuvent être observés à l'arrêt brutal du traitement.

Le détail des EI de morphine est listé dans le RCP en vigueur de la spécialité.

Tout EIG non listé dans les RCP des médicaments à l'étude ou dans ce chapitre sera considéré comme un EIG inattendu. Les EI non listés correspondent à des « faits nouveaux ».

- Concernant les traitements auxiliaires et les médicaments d'urgence administrés lors de la prise en charge, les EI attendus sont détaillés dans les RCP respectifs de ces médicaments utilisés dans le cadre de leurs AMM. Ces effets seront pris en compte et analysés au regard d'une interaction potentielle avec le traitement protocolaire.

- De même de nombreux médicaments et dispositifs médicaux peuvent être utilisés de façon concomitante aux traitements protocolaires, il est cependant impossible de tous les décrire. Dans la mesure où ils sont utilisés en conformité avec leurs indications officielles, leurs EI attendus sont listés dans les documents références que sont les RCP ou notices /manuels d'utilisation.

9.3.2. Concernant le protocole :

Il n'existe pas de risque spécifique au protocole, au choix thérapeutique ou à la randomisation dans le contexte d'urgence (Cf. chapitre risques) : les questionnaires d'évaluation de la douleur font ainsi partie de l'évaluation habituelle de la douleur. Néanmoins le patient peut ressentir de l'anxiété quant à la randomisation ou à une difficulté personnelle à comprendre / remplir les questionnaires.

9.3.3. Concernant la pathologie :

La population cible de cette étude est constituée de patients douloureux dont les tableaux cliniques et les étiologies sont extrêmement variés rendant impossible

l'identification exhaustive des EvI et EvIG attendus pour chaque type de pathologie traitée.

9.4. GESTION DES EVENEMENTS INDESIRABLES

9.4.1. Recueil des EvI/EI

Tout EvI/EI, qu'il soit attendu ou inattendu, grave ou non grave, devrait être recueilli en temps réel dans l'eCRF de l'étude.

Toutefois dans le cadre de ce protocole ne nécessitent pas de relevé, dans la section vigilance du CRF, et pas de transmission au promoteur si un critère de gravité est présent :

- Les symptômes et complications en lien avec la pathologie justifiant le traitement antalgique.
- Les EI des médicaments et DM pour lesquels aucune interaction avec le traitement antalgique ne peut être raisonnablement suspectée.

9.4.2. Notification des EvIG/EIG

Tous les évènements indésirables non graves ou graves survenant au cours de l'étude seront traités conformément aux procédures établies par le promoteur selon la réglementation en vigueur.

Tout EvIG / EIG qu'il soit attendu ou inattendu doit être notifié sans délai au promoteur à compter du jour où l'investigateur en a connaissance par l'intermédiaire du formulaire de notification eCRF, à l'exception de ceux mentionnés ci-dessus comme ne justifiant pas de saisie dans l'e CRF

Les informations renseignées sur ce formulaire ainsi que sur les documents joints doivent être complètes, précises, claires (ne pas mettre d'abréviation…) et codées.

La grossesse, le surdosage, le mésusage, les erreurs ou risques d'erreurs, les défauts qualité font également l'objet d'une notification au promoteur même s'il n'y a pas d'événement indésirable associé.

9.4.3. Période de notification

Tout EIG doit être notifié au promoteur s'il survient pour un participant à la recherche :

- A partir de la date de signature du consentement,
- Pendant toute la durée de suivi du participant prévue par l'essai, donc jusqu'à sortie de l'unité dans le cadre du présent protocole
- Après la fin du suivi du patient et sans limitation de durée si l'investigateur a connaissance d'un EIG susceptible d'être dû au traitement expérimental.

9.4.4. Comité indépendant de surveillance (CIS)

Notre essai s'inscrit dans une approche de soins courants sur le plan des stratégies thérapeutiques, de même les produits étant utilisés dans le cadre du RCP, la constitution d'un comité de surveillance indépendant ne parait pas nécessaire à notre recherche.

9.5. MODALITES ET DUREE DU SUIVI DES PERSONNES SUITE A LA SURVENUE D'EVENEMENTS INDESIRABLES

Tout évènement notamment grave doit être suivi jusqu'à guérison, consolidation ou décès (évènement clos).

10. ASPECTS ADMINISTRATIFS ET REGLEMENTAIRES

10.1. DROIT D'ACCES AUX DONNEES ET DOCUMENTS SOURCE

Les données médicales de chaque patient ne seront transmises qu'au promoteur ou toute personne dûment habilitée par celui-ci et, le cas échéant aux autorités sanitaires habilitées, dans les conditions garantissant leur confidentialité.

Le promoteur et les autorités de tutelle pourront demander un accès direct au dossier médical pour vérification des procédures et/ou des données de l'essai clinique et dans les limites autorisées par les lois et réglementations.

Les données recueillies lors de l'essai feront l'objet d'un traitement informatique, en conformité avec les exigences de la CNIL.

10.2. MONITORING DE L'ESSAI

Le monitoring sera assuré par le département promotion de la Direction de la recherche. Un Attaché de Recherche Clinique (ARC) se rendra régulièrement sur chaque site (investigateur et pharmacie) afin de procéder au contrôle qualité des données rapportées dans les cahiers d'observations.

Le protocole a été classé selon le niveau risque estimé pour le patient se prêtant à la recherche. Il sera suivi de la manière suivante :

Risque B : risque prévisible proche de celui des soins usuels

Les visites de monitoring sur site seront organisées après rendez-vous avec l'investigateur. Les ARC devront pouvoir consulter sur chaque site:

- les cahiers de recueil de données des patients inclus,
- les dossiers médicaux et infirmiers des patients,
- le classeur investigateur
- les lieux de stockage et dispensation des médicaments

10.3. INSPECTION / AUDIT

Dans le cadre de la présente étude, une inspection ou un audit pourra avoir lieu. Le promoteur et/ou les centres participants doivent pouvoir donner l'accès aux données aux inspecteurs ou auditeurs.

Toutes les données, tous les documents et rapports peuvent faire l'objet d'audits et d'inspections réglementaires sans que puisse être opposé le secret médical.

10.4. CONSIDERATIONS ETHIQUES

10.4.1. *Consentement éclairé écrit*

L'investigateur s'engage à obtenir le consentement libre et éclairé de la personne, recueilli par écrit, après lui avoir délivré l'information sur le protocole. Il lui remettra un exemplaire de la note d'information et un formulaire de recueil de consentement. La personne ne pourra être incluse dans l'étude qu'après avoir pris connaissance de la note d'information et signé et daté le formulaire de recueil de consentement après avoir disposé, si nécessaire, d'un temps de réflexion.

L'investigateur doit également signer et dater le formulaire de recueil de consentement. Ces deux documents seront délivrés sur papier en 2 exemplaires minimum afin que le patient et l'investigateur puissent chacun en garder un exemplaire. L'original de l'investigateur sera classé dans le classeur investigateur. En cas de consentement signé en duplicate, l'investigateur conserve l'original, le duplicata est remis au patient.

Il faut noter que les mineurs et les majeurs sous tutelle ou curatelle ne seront pas inclus dans notre étude. Il n'y aura pas donc de modalités particulières de recueil de consentement de ces cas particuliers.

10.4.2. *Modalités de recueil du consentement en cas d'urgence*

Non applicable.

10.4.3. *Comité de Protection des Personnes*

Le promoteur s'engage à soumettre le projet d'étude à l'autorisation préalable d'un Comité de Protection des Personnes (CPP).

10.5. DECLARATION AUX AUTORITES COMPETENTES

Le présent protocole fera l'objet d'une demande d'autorisation auprès de l'ANSM.

10.6. AMENDEMENTS AU PROTOCOLE

Les demandes de modifications substantielles seront adressées par le promoteur pour autorisation auprès de l'ANSM et pour autorisation/information au comité de protection des personnes concerné conformément à la loi en vigueur et ses arrêtés d'application.

Le protocole modifié devra faire l'objet d'une version actualisée datée.

Les formulaires d'information et de recueil consentement du patient feront l'objet de modification si nécessaire.

10.7. FICHIER DES PERSONNES SE PRETANT AUX RECHERCHES BIOMEDICALES

Non applicable.

10.8. FINANCEMENT ET ASSURANCE

Le promoteur assure le financement de l'étude et souscrit pour toute la durée de l'étude une police d'assurance garantissant les conséquences pécuniaires de sa responsabilité civile, conformément à la réglementation, ainsi que celle de tout médecin impliqué dans la réalisation de l'étude.

10.9. REGLES RELATIVES A LA PUBLICATION

L'étude sera enregistrée sur le site web libre accès « Clinical Trial » avant l'inclusion du 1er patient dans cette étude.

Les communications et rapports scientifiques correspondant à cette étude seront réalisés sous la responsabilité de l'investigateur coordonnateur de l'étude avec l'accord des investigateurs responsables. Les coauteurs du rapport et des publications seront les investigateurs et les cliniciens impliqués, en proportion du nombre de sujets inclus et de leur implication dans l'étude, ainsi que le biostatisticien et les chercheurs associés.

Les règles de publications suivront les recommandations internationales (N Engl J Med, 1997;336 :309-315).

Une copie de la publication sera remise au CHU de Nantes, responsable de l'étude, qui devra nécessairement être cité.

Par ailleurs, les publications résultants des projets financés dans le cadre des appels à projet du Ministre de la Santé doivent obligatoirement porter la mention suivante : *"This study was supported by a grant from the French Ministry of Health (acronyme du programme, année du programme, n° d'enregistrement : ex PHRC 2014 XXXX, ou PREPS 2014 XXXX, …)".*

Enfin, le promoteur incrémentera la base de l'Union Européenne des résultats de l'essai clinique dès que la publication princeps issue de ces travaux sera effective et ce, afin de ne pas porter préjudice à la protection de la propriété intellectuelle.

10.10. DEVENIR DES ECHANTILLONS BIOLOGIQUES

Non applicable.

10.11. ARCHIVAGE

Les documents nommées ci-dessous, seront archivés par le nom de l'étude dans les locaux de l'Unité d'Investigation Clinique 8 Urgences du CHU de Nantes, jusqu' à la fin de la période d'utilité pratique :

- Protocole et annexes, ainsi que des amendements éventuels.
- Formulaires d'information et consentements originaux signées.
- Données individuelles (copies authentifiées de données brutes).
- Worksheets et documents de suivi.

- Analyses statistiques.

- Rapport final de l'étude.

A la fin de la période d'utilité pratique, l'ensemble des documents à archiver, tels que définis dans la procédure de « classement et archivage des documents liés aux recherches portant sur la personne humaine » sera transféré au Service central des Archives-Hôpital de Nantes et sera placé sous la responsabilité du Promoteur pendant 15 ans après la fin de l'étude conformément aux pratiques institutionnelles.

Aucun déplacement ou destruction ne pourra être effectué sans l'accord du promoteur, au terme de ces 15 ans, le promoteur sera consulté pour destruction. Toutes les données, tous les documents et rapports pourront faire l'objet d'audit ou d'inspection.

LISTE DES ANNEXES

Annexe 1 : Bibliographie
Annexe 2 : Listing investigateurs (version 1 du 07/09/2018)
Annexe 3 : Résumé du protocole (version 1 du 07/09/2018)
Annexe 4 : Echelle Verbale Numérique (version 1 du 07/09/2018)
Annexe 5 : Score de Ramsay (version 1 du 07/09/2018)
Annexe 6 : RCP Chlorure de Sodium (version 1 du 07/09/2018)
Annexe 7: RCP Paracétamol (version 1 du 07/09/2018)
Annexe 8: RCP Morphine (Version 1 du 07/09/2018)
Annexe 9: Détails du budget ADAMOPA (Version 1 du 07/09/2018).
Annexe 10 : Lettres d'engagement des centres associés

ANNEXE 1 : BIBLIOGRAPHIE

1. Lanser, P., et S. Gesell. 2001. « Pain Management: The Fifth Vital Sign ». *Healthcare Benchmarks* 8 (6): 68-70, 62

2. Mccaig, Linda F., Catharine W. Burt, Ed D, Division Health, et Care Statistics. 2005. « National hospital ambulatory medical care survey: 2003 emergency department summary ». Division of Health Care Statistics, Centers for Desease Control and Prevention, Department of Health and Human Services.

3. Boccard, E., F. Adnet, P.-Y. Gueugniaud, A. Filipovics, et A. Ricard-Hibon. 2011. « Prise en charge de la douleur chez l'adulte dans des services d'urgences en France en 2010 ». *Annales françaises de médecine d'urgence* 1 (5): 312. https://doi.org/10.1007/s13341-011-0094-4.

4. Mura, Paolo, Elisabetta Serra, Franco Marinangeli, Sebastiano Patti, Mario Musu, Ilenia Piras, Maria Valeria Massidda, Giorgio Pia, Maurizio Evangelista, et Gabriele Finco. 2017. « Prospective study on prevalence, intensity, type, and therapy of acute pain in a second-level urban emergency department ». *Journal of Pain Research* 10 (décembre): 2781-88. https://doi.org/10.2147/JPR.S137992.

5. Berben, Sivera A. A., Tineke H. J. M. Meijs, Robert T. M. van Dongen, Arie B. van Vugt, Lilian C. M. Vloet, Joke J. Mintjes-de Groot, et Theo van Achterberg. 2008. « Pain prevalence and pain relief in trauma patients in the Accident & Emergency department ». *Injury* 39 (5): 578-85. https://doi.org/10.1016/j.injury.2007.04.013.

6. Chang, Hsien-Yen, Matthew Daubresse, Stefan P. Kruszewski, et G. Caleb Alexander. 2014. « Prevalence and Treatment of Pain in EDs in the United States, 2000 to 2010 ». *The American Journal of Emergency Medicine* 32 (5): 421-31. https://doi.org/10.1016/j.ajem.2014.01.015.

7. Guéant, S. Taleb, A; Borel-Kühner, J; Cauterman, M; Raphael, M; Nathan, G; Ricard-Hibon, A. « Qualité de la prise en charge de la douleur à l'urgence: résultats d'une étude prospective multicentrique». European Journal of Anaesthesiology: 2011: 28(2), 97-105 doi: 10.1097 / EJA.0b013e3283418fb0

8.Chen, Esther H., Frances S. Shofer, Anthony J. Dean, Judd E. Hollander, William G. Baxt, Jennifer L. Robey, Keara L. Sease, et Angela M. Mills. s. d. « Gender Disparity in Analgesic Treatment of Emergency Department Patients with Acute Abdominal Pain ». *Academic Emergency Medicine* 15 (5): 414-18. https://doi.org/10.1111/j.1553-2712.2008.00100.x.

9.Dale, Jostein, et Lars Petter Bjørnsen. 2015. « Assessment of pain in a Norwegian Emergency Department ». *Scandinavian Journal of Trauma, Resuscitation and Emergency Medicine* 23 (1): 86. https://doi.org/10.1186/s13049-015-0166-3.

10. Todd, Knox H., James Ducharme, Manon Choiniere, Cameron S. Crandall, David E. Fosnocht, Peter Homel, et Paula Tanabe. 2007. « Pain in the Emergency Department: Results of the Pain and Emergency Medicine Initiative (PEMI) Multicenter Study ». *The Journal of Pain* 8 (6): 460-66. https://doi.org/10.1016/j.jpain.2006.12.005.

11. Vivien, Benoît, Frédéric Adnet, Vincent Bounes, Gérard Chéron, Xavier Combes, Jean-Stéphane David, Jean-François Diependaele, et al. 2010. « Sédation et analgésie en structure d'urgence (aéactualisation de la Conférence d'experts de la Sfar of 1999) ». *Annales Françaises d'Anesthésie et de Réanimation* 29 (12): 934-49. https://doi.org/10.1016/j.annfar.2010.10.005.

12. Galinski M, Adnet F. Prise en charge de la douleur aiguë en médecine d'urgence. Reanimation ; 2007 : 16, 652—659

13. Patanwala, Asad E, Samuel M Keim, et Brian L Erstad. 2010. « Intravenous Opioids for Severe Acute Pain in the Emergency Department Intravenous Opioids for Severe Acute Pain in the Emergency Department ». *Annals of Pharmacotherapy* 44 (11): 1800-1809. https://doi.org/10.1345/aph.1P438.

14. Ricard-Hibon, Agnes, Vanessa Belpomme, Charlotte Chollet, Marie-Laure Devaud, Frederic Adnet, Stephen Borron, Jean Mantz, et Jean Marty. 2008. « Compliance with a Morphine Protocol and Effect on Pain Relief in Out-of-Hospital Patients ». *The Journal of Emergency Medicine* 34 (3): 305-10. https://doi.org/10.1016/j.jemermed.2007.06.003.

15. Birnbaum, Adrienne, David Esses, Polly E. Bijur, Lynne Holden, et E. John Gallagher. 2007. « Randomized Double-Blind Placebo-Controlled Trial of Two Intravenous Morphine Dosages (0.10 mg/kg and 0.15 mg/kg) in Emergency Department Patients With Moderate to Severe Acute Pain ». Annals of Emergency Medicine 49 (4): 445-453.e2. https://doi.org/10.1016/j.annemergmed.2006.06.030.

16. Lvovschi, Virginie, Frédéric Aubrun, Pascale Bonnet, Anna Bouchara, Mouhssine Bendahou, Béatrice Humbert, Pierre Hausfater, et Bruno Riou. 2008. « Intravenous morphine titration to treat severe pain in the ED ». The American Journal of Emergency Medicine 26 (6): 676‑82. https://doi.org/10.1016/j.ajem.2007.10.025.

17.Bijur, Polly E., Mark K. Kenny, et E. John Gallagher. 2005. « Intravenous Morphine at 0.1 mg/kg Is Not Effective for Controlling Severe Acute Pain In the Majority of Patients ». *Annals of Emergency Medicine* 46 (4): 362-67. https://doi.org/10.1016/j.annemergmed.2005.03.010.

18. Bounes, Vincent, Béatrice Charriton-Dadone, Jacques Levraut, Cyril Delangue, Françoise Carpentier, Stéphanie Mary-Chalon, Vanessa Houze-Cerfon, Agnès Sommet, Charles-Henri Houze-Cerfon, et Michael Ganetsky. 2017. « Predicting Morphine Related Side Effects in the ED: An International Cohort Study ». *The American Journal of Emergency Medicine* 35 (4): 531-35. https://doi.org/10.1016/j.ajem.2016.11.053.

19. Tavassoli, N., Lapeyre-Mestre, M., Sommet, A., Montastruc, J. et (2009), Taux de notification des réactions indésirables au système français de pharmacovigilance avec trois médicaments antalgiques de stade 2: dextropropoxyphène, tramadol et codéine (en combinaison avec du paracétamol). British Journal of Clinical Pharmacology, 68: 422-426. doi: 10.1111 / j.1365-2125.2009.03472.x

20. Bijur, Polly E., Mark K. Kenny, et E. John Gallagher. 2005. « Intravenous Morphine at 0.1 mg/kg Is Not Effective for Controlling Severe Acute Pain In the Majority of Patients ». *Annals of Emergency Medicine* 46 (4): 362-67. https://doi.org/10.1016/j.annemergmed.2005.03.010.

21. Smith, Howard S. 2011. « Perioperative Intravenous Acetaminophen and NSAIDs ». *Pain Medicine (Malden, Mass.)* 12 (6): 961-81. https://doi.org/10.1111/j.1526-4637.2011.01141.x.

22. Norris, William, Angelo H. Paredes, et James H. Lewis. 2008. « Drug-Induced Liver Injury in 2007 ». Current Opinion in Gastroenterology 24 (3): 287-97. https://doi.org/10.1097/MOG.0b013e3282f9764b.

23. Gulmez, Sinem Ezgi, Dominique Larrey, Georges-Philippe Pageaux, Jacques Bernuau, Franco Bissoli, Yves Horsmans, Douglas Thorburn, et al. 2015. « Liver Transplant Associated with Paracetamol Overdose: Results from the Seven-Country SALT Study ». British Journal of Clinical Pharmacology 80 (3): 599-606. https://doi.org/10.1111/bcp.12635.

24. Larson, Anne M., Julie Polson, Robert J. Fontana, Timothy J. Davern, Ezmina Lalani, Linda S. Hynan, Joan S. Reisch, et al. 2005. « Acetaminophen-Induced Acute Liver Failure: Results of a United States

Multicenter, Prospective Study ». Hepatology (Baltimore, Md.) 42 (6): 1364-72. https://doi.org/10.1002/hep.20948.

25. Boudjemai, Y., P. Mbida, V. Potinet-Pagliaroli, F. Géffard, G. Leboucher, J. -L. Brazier, B. Allenet, et B. Charpiat. 2013. « Patients' knowledge about paracetamol (acetaminophen): A study in a French hospital emergency department ». Annales Pharmaceutiques Françaises 71 (4): 260-67. https://doi.org/10.1016/j.pharma.2013.03.001.

26. Roberts, Emmert, Vanessa Delgado Nunes, Sara Buckner, Susan Latchem, Margaret Constanti, Paul Miller, Michael Doherty, et al. 2015. « Paracetamol: Not as Safe as We Thought? A Systematic Literature Review of Observational Studies ». Annals of the Rheumatic Diseases, février, annrheumdis-2014-206914. https://doi.org/10.1136/annrheumdis-2014-206914

27. Gulmez, Sinem Ezgi, Dominique Larrey, Georges-Philippe Pageaux, Severine Lignot, Régis Lassalle, Jérémy Jové, Angelo Gatta, et al. 2013. « Transplantation for Acute Liver Failure in Patients Exposed to NSAIDs or Paracetamol (Acetaminophen): The Multinational Case-Population SALT Study ». Drug Safety 36 (2): 135-44. https://doi.org/10.1007/s40264-012-0013-7.

28. Wininger, Steven J., Howard Miller, Harold S. Minkowitz, Mike A. Royal, Robert Y. Ang, James B. Breitmeyer, et Neil K. Singla. 2010. « A Randomized, Double-Blind, Placebo-Controlled, Multicenter, Repeat-Dose Study of Two Intravenous Acetaminophen Dosing Regimens for the Treatment of Pain After Abdominal Laparoscopic Surgery ». Clinical Therapeutics 32 (14): 2348-69. https://doi.org/10.1016/j.clinthera.2010.12.011.

29. Atef, Ahmed, et Ahmed Aly Fawaz. 2008. « Intravenous Paracetamol Is Highly Effective in Pain Treatment after Tonsillectomy in Adults ». *European Archives of Oto-Rhino-Laryngology* 265 (3): 351-55. https://doi.org/10.1007/s00405-007-0451-5.

30. Kemppainen, Tatu, Hannu Kokki, Henri Tuomilehto, Juha Seppä, et Juhani Nuutinen. s. d. « Acetaminophen Is Highly Effective in Pain Treatment After Endoscopic Sinus Surgery ». *The Laryngoscope* 116 (12): 2125-28. https://doi.org/10.1097/01.mlg.0000239108.12081.35.

31. Gimbel, J. Leclerc, A. Royal, M. « Efficacy and Safety of IV Acetaminophen in the Treatment of Pain Following Primary Total Hip Arthroplasty: Results of a Double-Blind, Randomized, Placebo-Controlled, Multiple-Dose, 24 Hour Study ». Pain Medicine. 9(1):141, 2008 https://insights.ovid.com/pain-medicine/pnmed/2008/01/000/efficacy-safety-iv-acetaminophen-treatment-pain/99/00130495.

32. Minkowitz, H. Leclerc, A. Mike A. Royal, Kenney, B. 2008 « Efficacy and Safety of IV Acetaminophen in the Treatment of Pain Following Vaginal Hysterectomy: Results of a Double-Blind, Randomized, Placebo-Controlled, Multiple-Dose, 24 Hour Study ». https://aapm.confex.com/aapm/2008am/techprogram/P2944.HTM.

33. De Oliveira GS Jr, Castro-Alves LJ, McCarthy RJ. Single-dose systemic acetaminophen to prevent postoperative pain: A meta-analysis of randomized controlled trials. Clin J Pain. 2015;31(1):86-93.

34. Elia, Nadia, Christopher Lysakowski, Martin R. TramAMPERSANDNUMBERSIGNx000E8, et r. 2005. « Does Multimodal Analgesia with Acetaminophen, Nonsteroidal Antiinflammatory Drugs, or Selective Cyclooxygenase-2 Inhibitors and Patient-controlled Analgesia Morphine Offer Advantages over Morphine Alone?Meta-analyses of Randomized Trials ». *Anesthesiology: The Journal of the American Society of Anesthesiologists* 103 (6): 1296-1304. https://doi.org/0000542-200512000-00025.

35. Remy, C., E. Marret, et F. Bonnet. 2005. « Effects of acetaminophen on morphine side-effects and consumption after major surgery: meta-analysis of randomized controlled trials ». *BJA: British Journal of Anaesthesia* 94 (4): 505-13. https://doi.org/10.1093/bja/aei085.

36. McNicol, E. D., A. Tzortzopoulou, M. S. Cepeda, M. B. D. Francia, T. Farhat, et R. Schumann. 2011. « Single-dose intravenous paracetamol or propacetamol for prevention or treatment of postoperative pain: a systematic review and meta-analysis ». *BJA: British Journal of Anaesthesia* 106 (6): 764-75. https://doi.org/10.1093/bja/aer107.

37. Jelacic, Srdjan, Laurent Bollag, Andrew Bowdle, Cyril Rivat, Kevin C. Cain, et Philippe Richebe. 2016. « Intravenous Acetaminophen as an Adjunct Analgesic in Cardiac Surgery Reduces Opioid Consumption But Not Opioid-Related Adverse Effects: A Randomized Controlled Trial ». *Journal of Cardiothoracic and Vascular Anesthesia* 30 (4): 997-1004. https://doi.org/10.1053/j.jvca.2016.02.010.

38. Altenau, Brie, Catrina C. Crisp, C. Ganga Devaiah, et Donna S. Lambers. 2017. « Randomized Controlled Trial of Intravenous Acetaminophen for Postcesarean Delivery Pain Control ». *American Journal of Obstetrics and Gynecology* 217 (3): 362.e1-362.e6. https://doi.org/10.1016/j.ajog.2017.04.030.

39. Aubrun, Frédéric, Karine Nouette Gaulain, Dominique Fletcher, Anissa Belbachir, Hélène Beloeil, Michel Carles, Philippe Cuvillon, et al. 2016. « Réactualisation de la recommandation sur la douleur postopératoire ». Anesthésie & Réanimation 2 (6): 421‑30. https://doi.org/10.1016/j.anrea.2016.09.006.Axelsson B, Stellborn P, Ström G (2008) Analgesic effect of paracetamol on cancer related pain in concurrent strong opioid therapy. A prospective clinical study. Acta Oncol 47:891-898

40. Kelly, Jennifer S., Yekaterina Opsha, Jennifer Costello, Daryl Schiller, et Eric T. Hola. 2014. « Opioid Use in Knee Arthroplasty after Receiving Intravenous Acetaminophen ». *Pharmacotherapy* 34 Suppl 1 (décembre): 22S-26S. https://doi.org/10.1002/phar.1518.41. Greenberg, Steven, Glenn S. Murphy, Michael J. Avram, Torin Shear, Jessica Benson, Kruti N. Parikh,Aashka Patel, et al. 2018. « Postoperative Intravenous Acetaminophen for Craniotomy Patients: A Randomized Controlled Trial ». *World Neurosurgery* 109 (janvier): e554-62. https://doi.org/10.1016/j.wneu.2017.10.021

42. Mamoun, Negmeldeen F., Peirong Lin, Nicole M. Zimmerman, Edward J. Mascha, Stephanie L. Mick, Steven R. Insler, Daniel I. Sessler, et Andra E. Duncan. 2016. « Intravenous Acetaminophen Analgesia after Cardiac Surgery: A Randomized, Blinded, Controlled Superiority Trial ». *The Journal of Thoracic and Cardiovascular Surgery* 152 (3): 881-889.e1. https://doi.org/10.1016/j.jtcvs.2016.04.078.

43. O'Neal, Jason B., Andrew A. Freiberg, Marc D. Yelle, Yandong Jiang, Chengwei Zhang, Yin Gu, Xiangyi Kong, Wenling Jian, Wesley T. O'Neal, et Jingping Wang. 2017. « Intravenous vs Oral Acetaminophen as an Adjunct to Multimodal Analgesia After Total Knee Arthroplasty: A Prospective, Randomized, Double-Blind Clinical Trial ». *The Journal of Arthroplasty* 32 (10): 3029-33. https://doi.org/10.1016/j.arth.2017.05.019.

44.Mahshidfar, Babak, Azadeh Sameti, Saeed Abbasi, Davood Farsi, Mani Mofidi, Peyman Hafezimoghadam, Popak Rahimzadeh, et Mahdi Rezai. 2016. « Can Intravenous Acetaminophen Reduce the Needs to More Opioids to Control Pain in Intubated Patients? » *Indian Journal of Critical Care Medicine* 20 (8): 465. https://doi.org/10.4103/0972-5229.188197.

45. Zernikow B, Smale H, Michel E, Hasan C, Jorch N, Andler C (2006) Paediatric cancer pain management using the WHO analgesic ladder—results of a prospective analysis from 2265 treatment days during a guality improvement study. Eur J Pain 10:587–595

46 Axelsson B, Stellborn P, Ström G (2008) Analgesic effect of paracetamol on cancer related pain in concurrent strong opioid therapy. A prospective clinical study. Acta Oncol 47:891-898

47. Israel, Fiona J., Greg Parker, Margaret Charles, et Liz Reymond. 2010. « Lack of Benefit from Paracetamol (Acetaminophen) for Palliative Cancer Patients Requiring High-Dose Strong Opioids: A

Randomized, Double-Blind, Placebo-Controlled, Crossover Trial ». *Journal of Pain and Symptom Management* 39 (3): 548-54. https://doi.org/10.1016/j.jpainsymman.2009.07.008.

48. Tasmacioglu, Buket, Isik Aydinli, Kader Keskinbora, Ali Ferit Pekel, Tamer Salihoglu, et Abdullah Sonsuz. 2009. « Effect of Intravenous Administration of Paracetamol on Morphine Consumption in Cancer Pain Control ». *Supportive Care in Cancer: Official Journal of the Multinational Association of Supportive Care in Cancer* 17 (12): 1475-81. https://doi.org/10.1007/s00520-009-0612-8.

49. Wiffen, Philip J., Sheena Derry, R. Andrew Moore, Ewan D. McNicol, Rae F. Bell, Daniel B. Carr, Mairead McIntyre, et Bee Wee. 2017. « Oral Paracetamol (Acetaminophen) for Cancer Pain ». *The Cochrane Database of Systematic Reviews* 7: CD012637. https://doi.org/10.1002/14651858.CD012637.pub2.50. Machado, Gustavo C., Chris G. Maher, Paulo H. Ferreira, Marina B. Pinheiro, Chung-Wei Christine Lin, Richard O. Day, Andrew J. McLachlan, et Manuela L. Ferreira. 2015. « Efficacy and Safety of Paracetamol for Spinal Pain and Osteoarthritis: Systematic Review and Meta-Analysis of Randomised Placebo Controlled Trials ». *BMJ (Clinical Research Ed.)* 350 (mars): h1225

50 Machado, Gustavo C., Chris G. Maher, Paulo H. Ferreira, Marina B. Pinheiro, Chung-Wei Christine Lin, Richard O. Day, Andrew J. McLachlan, et Manuela L. Ferreira. 2015. « Efficacy and Safety of Paracetamol for Spinal Pain and Osteoarthritis: Systematic Review and Meta-Analysis of Randomised Placebo Controlled Trials ». *BMJ (Clinical Research Ed.)* 350 (mars): h1225.

51. Farnia MR, Babaei R, Shirani F, et al. Analgesic effect of paracetamol combined with low-dose morphine versus morphine alone on patients with biliary colic: A double blind, randomized controlled trial. World J Emerg Med. 2016;7(1):25-9

52. Milojevic, K, J. P Cantineau, L Simon, S Bataille, R Ruiz, B Coudert, N Simon, et Y Lambert. 2001. « Douleur aiguë intense en médecine d'urgence. Les clefs d'une analgésie efficace ». *Annales Françaises d'Anesthésie et de Réanimation* 20 (9): 745-51. https://doi.org/10.1016/S0750-7658(01)00482-8.

53. Aida, Sumihisa, Hideyoshi Fujihara, Kiichiro Taga, Satoru Fukuda, et Koki Shimoji. 2000. « Involvement of presurgical pain in preemptive analgesia for orthopedic surgery: a randomized double blind study ». PAIN 84 (2): 169‑73. https://doi.org/10.1016/S0304-3959(99)00196-7.

54. Zare, Mohammad Amin, Alireza Hassan Ghalyaie, Marzieh Fathi, Davood Farsi, Saeed Abbasi, et Peyman Hafezimoghadam. 2014. « Oral Oxycodone plus Intravenous Acetaminophen versus Intravenous Morphine Sulfate in Acute Bone Fracture Pain Control: A Double-Blind Placebo-Controlled Randomized Clinical Trial ». *European Journal of Orthopaedic Surgery & Traumatology: Orthopedie Traumatologie* 24 (7): 1305-9. https://doi.org/10.1007/s00590-013-1392-x.

55. Boyle, Martin, Lisa Nicholson, Maureen O'Brien, Gordon M. Flynn, David W. Collins, William R. Walsh, et David Bihari. 2010. « Paracetamol Induced Skin Blood Flow and Blood Pressure Changes in Febrile Intensive Care Patients: An Observational Study ». Australian Critical Care: Official Journal of the Confederation of Australian Critical Care Nurses 23 (4): 208-14. https://doi.org/10.1016/j.aucc.2010.06.004.

56.Schell-Chaple, Hildy M., Kathleen D. Liu, Michael A. Matthay, Daniel I. Sessler, et Kathleen A. Puntillo. 2017. « Effects of IV Acetaminophen on Core Body Temperature and Hemodynamic Responses in Febrile Critically Ill Adults: A Randomized Controlled Trial ». Critical Care Medicine 45 (7): 1199-1207. https://doi.org/10.1097/CCM.0000000000002340.

57. Mrozek, S., J. -M. Constantin, E. Futier, M. Zenut, G. Ghardes, S. Cayot-Constantin, M. Bonnard, N. Ait-Bensaid, A. Eschalier, et J. -E. Bazin. 2009. « Étude prospective de l'incidence des hypotensions artérielles induites par l'injection intraveineuse de paracétamol

en réanimation ». Annales Françaises d'Anesthésie et de Réanimation 28 (5): 448-53. https://doi.org/10.1016/j.annfar.2009.01.01858. WILLIAM H. CORDELL, KELLY K. KEENE, BEVERLY K. GILES,JAMES B. JONES, ,JAMES H. JONES, AND EDWARD J. BRIZENDINE.2002 "The High Prevalence of Pain in Emergency Medical Care". The American Journal of Emergency Medicine 2002; 20: 165-169.

59. Leinisch, Elke, Stefan Evers, Nina Kaempfe, Christoffer Kraemer, Petra Sostak, Tim Jürgens, Andreas Straube, et Arne May. 2005. « Evaluation of the Efficacy of Intravenous Acetaminophen in the Treatment of Acute Migraine Attacks: A Double-Blind, Placebo-Controlled Parallel Group Multicenter Study ». *Pain* 117 (3): 396-400. https://doi.org/10.1016/j.pain.2005.07.002.

60. Craig M, Jeavons R, Probert J, Benger J. Randomised comparison of intravenous paracetamol and intravenous morphine for acute traumatic limb pain in the emergency department. Emerg Med J 2012;29:37–9.

61. Esmailian M, Moshiri R, Zamani M. Comparison of the analgesic effect of intravenous acetaminophen and morphine sulfate in rib fracture; a randomized double-blind clinical trial. Emerg (Tehran). 2015;3(3):99-102

62. Bounes, Vincent, Sandrine Charpentier, Charles-Henri Houze-Cerfon, Cédric Bellard, et Jean Louis Ducassé. 2008. « Is there an ideal morphine dose for prehospital treatment of severe acute pain? A randomized, double-blind comparison of 2 doses ». The American Journal of Emergency Medicine 26 (2): 148 54. https://doi.org/10.1016/j.ajem.2007.04.020

63. MacIntyre PA, Scott DA, Schug SA, et al. Acute Pain Management: Scientific Evidence. 3rd ed. Dallas, TX: Australian and New Zealand College of Anaethetists and Faculty of Pain Medicine; 2010

64. Todd KH, Funk KG, Funk JP, Bonacci R. Clinical significance of reported changes in pain severity. Ann Emerg Med. 1996;27:485-9.

65. Bijur PE, Silver W, Gallagher EJ. Reliability Reliability of the visual analog scale for measurment of acute pain. Acad Emerg Med 2001;8:1153-7

66. Bijur PE, Latimer CT, Gallagher EJ. Validation of a verbally administered numerical rating scale of acute pain for use in the emergency department. Acad Emerg Med 2003;10: 390-2

67. Holdgate, Anna, Stephen Asha, Jonathan Craig, et Jennifer Thompson. 2003. « Comparison of a Verbal Numeric Rating Scale with the Visual Analogue Scale for the Measurement of Acute Pain ». Emergency Medicine (Fremantle, W.A.) 15 (5‑6): 441‑46.

68. Ricard-Hibon, A., et V. Bounes. 2012. « Sédation et analgésie en structure d'urgence. Quelles sédation et analgésie chez le patient en ventilation spontanée en structure d'urgence ? » *Annales Françaises d'Anesthésie et de Réanimation*, Sédation et analgésie en structure d'urgence, 31 (4): 295-312. https://doi.org/10.1016/j.annfar.2012.01.007

69. Blettery B, Ebrahim L, Honnart D, Aube H. Les échelles de mesure de la douleur dans un service d'accueil des urgences. Réan Urg 1996;5:691-697.

70. Berthier F, Potel G, Leconte P, et al. Comparative study of methods of measuring acute pain intensity in an ED. Am J Emerg Med. 1998;16:132-6

71. Hansen MS, Dahl JB. Limited evidence for intranasal fentanyl in the emergency department and the prehospital setting — a systematic review. Dan Med J 2013;60:A4563.

72. Bounes V, Ducassé JL, Bona AM, Battefort F, Houze-Cerfon C-H, Lauque D. Nebulized morphine for analgesia in an emergency setting. J Opioid Manag 2009;5:23—6.

73. Ducassé, J.-L., V. Bounes, A. Momo Bona, F. Battefort, et D. Lauque. 2008. « La morphine par voie inhalée agit-elle sur les douleurs aiguës sévères ? » /data/revues/09939857/0020001S/26_3/, mars. http://www.em-consulte.com/en/article/110560.

74.Yeh, Yu-Chen, et Prabashni Reddy. s. d. « Clinical and Economic Evidence for Intravenous Acetaminophen ». Pharmacotherapy: The Journal of Human Pharmacology and Drug Therapy 32 (6): 559‑79. https://doi.org/10.1002/j.1875-9114.2011.01085.x.

75. Pettersson, P. Holmér, A. Öwall, et J. Jakobsson. 2004. « Early Bioavailability of Paracetamol after Oral or Intravenous Administration ». Acta Anaesthesiologica Scandinavica 48 (7): 867‑70. https://doi.org/10.1111/j.0001-5172.2004.00452.x.

762. Gallagher, E. John, David Esses, Conroy Lee, Michael Lahn, et Polly E. Bijur. 2006. « Randomized Clinical Trial of Morphine in Acute Abdominal Pain ». *Annals of Emergency Medicine* 48 (2): 150-160.e4. https://doi.org/10.1016/j.annemergmed.2005.11.020.

77. Chang, Andrew K., Polly E. Bijur, Robert H. Meyer, Mark K. Kenny, Clemencia Solorzano, et E. John Gallagher. 2006. « Safety and Efficacy of Hydromorphone as an Analgesic Alternative to Morphine in Acute Pain: A Randomized Clinical Trial ». *Annals of Emergency Medicine* 48 (2): 164-72. https://doi.org/10.1016/j.annemergmed.2006.03.005.

78. Galinski M, Dolveck F, Borron S, Tual L, Van Laer V, Lardeur J, et al. A randomized, double blind study comparing morphine with fentanyl in prehospital analgesia. Am J Emerg Med 2005;23:114-119

ANNEXE 2: LISTING INVESTIGATEUR

NOM ET PRENOM	Spécialité	Fonction	Nom de l'établissement	Nom et adresse du service de rattachement	Téléphone, fax et e-mail	N° RPPS
Dr Céline LONGO	URGENCES	PH	CHU NANTES	CHU Nantes Urgences SAMU 44 de Nantes<br>1 place Alexis Ricordeau, 44093 Nantes	Tel : 02 53 48 20 44<br>Celine.longo@chu-nantes.fr	1000253821
Pr LARIBI Saïd	URGENCES	PU-PH	CHRU TOURS	CHRU Tours Urgences SAMU 37 SMUR de Tours<br>2 bd Tonnellé, 37000 Tours	Tel: 02 47 47 90 22<br>S.LARIBI@chu-tours.fr/	10003748919
Dr QUERELLOU Emgan	URGENCES	PU-PH	CHRU BREST	CHRU Brest/Site la Cavale Blanche SAMU29<br>Boulevard Tanguy Prigent, 29200 Brest	Tel : 02 98 22 33 33<br>gael-emgan.querellou@chu-brest.fr	10002673985
Dr MAIGNAN Maxime	URGENCES	MCU-PH	CHU GRENOBLE ALPES	CHU Grenoble Alpes, SAMU 38-SMUR Grenoble Boulevard de la Chantourne, 38700 La Tronche	Tel : 04 76 76 75 75<br>MMaignan@chu-grenoble.fr	10100074698
Dr GALINSKI Michel	URGENCES	PH	CHU BORDEAUX	Groupe hospitalier Pellegrin Service des Urgences<br>Place Amélie Raba Léon, 33076 Bordeaux	Tel : 05 57 82 00 02<br>michel.galinski@chu-bordeaux.fr	10001166791
Dr Philippe FRADIN	URGENCES	PH	CHD VENDEE	CHD Vendée SAMU 85 SMUR de La Roche Sur Yon Bd Stéphane Moreau, 85600 La Roche sur Yon	Tel : 02 51 44 61 61<br>Philippe.fradin@chd-vendee.fr	10002574308
Dr HOURDIN Nicolas	URGENCES	PH	CH CHATEAUBRIANT	CH Châteaubriant Urgences SMUR de Châteaubriant<br>9 rue de Verdun, 44146 Châteaubriant	Tel : 02 40 55 88 88<br>Nicolas.hourdin@ch-cnp.fr	10002590007
Pr ROY Pierre-Marie	URGENCES	PU-PH	CHU ANGERS	CHU Angers Département de Médecine d'Urgence, Pôle 4 USSAR<br>4 rue Larrey, 49100 Angers	Tel : 02 41 35 36 37<br>PMRoy@chu-angers.fr	10100074227

ANNEXE 3: RESUME DU PROTOCOLE

**Titre de l'étude**	Analgésie des douleurs aiguës aux urgences : Essai randomisé de non infériorité de la Morphine seule versus l'association Morphine et Paracétamol
**Mots clés**	Douleur aiguë, analgésie aux urgences, morphine, paracétamol
**Promoteur de l'étude**	**CHU DE NANTES**
**Investigateur coordonnateur**	Dr Céline LONGO
**Nombre de centres prévus**	*8*
**Planning de l 'étude**	❖ Durée totale : *30 mois*<br>❖ Période de recrutement : *30 mois*<br>❖ Durée de traitement par patient : *1h*<br>❖ Durée de suivi par patient : *Jusqu'à la fin de prise en charge aux urgences.*
**Type et design de l'étude**	Etude interventionnelle sur médicament<br>❖ multicentrique nationale<br>❖ Phase : 4<br>❖ Contrôlée<br>❖ Randomisée<br>❖ Double insu<br>❖ Prospective
**Nombre de cas prévisionnel**	572
**Objectifs de l'étude**	***Objectif principal :***<br>Evaluer la non-infériorité à 30 minutes de la morphine seule en intraveineux à l'association paracétamol et morphine en intraveineux chez les patients pris en charge aux urgences pour une douleur aiguë d'intensité modérée à sévère (EVN supérieure à égale à 5), dans un groupe de patients ayant une douleur traumatique et dans un groupe de patients ayant une douleur non traumatique.<br>***Objectif(s) secondaire(s) :***<br>1/ Evaluer la non-infériorité à 30 minutes de la morphine seule en intraveineux à l'association paracétamol et morphine en intraveineux chez les patients pris en charge aux urgences pour une douleur aiguë d'intensité modérée à sévère (EVN supérieure à égale à 5), dans la population combinée des patients ayant une douleur traumatique et non traumatique.<br>2/ Comparer la réduction de la douleur, calculée suivant l'Échelle Verbale Numérique (EVN), à 10, 20, 30, 45 et 60 minutes de la première injection du médicament antalgique entre le groupe morphine-placebo et le groupe morphine-paracétamol.<br>3/ Comparer la consommation de morphine dans les deux bras durant les 30 premières minutes d'administration des produits à l'étude.<br>4/ Comparer le taux de succès (EVN inférieure ou égale à 3) de l'analgésie des patients à 30 minutes de la première injection de l'antalgique, dans les deux bras.

	5/ Estimer et comparer la fréquence des effets indésirables dans les deux groupes de traitement à 10, 20, 30, 45 et 60 min de la première injection de l'antalgique.<br>6/ Comparer la nécessité d'une analgésie de secours à 30, 45 et 60 min de la première injection d'antalgique dans les deux groupes.<br>7/ Comparer la modification des signes vitaux à 10, 20, 30, 45 et 60 min de la première injection de l'antalgique dans les 2 groupes.<br>8/ Décrire la distribution de l'EVN à 10, 20, 30, 45 et à 60 min, dans les deux groupes.
**Critères de jugement**	***<u>Critère de jugement principal :</u>***<br>Différence entre le score de l'Echelle Verbale Numérique (EVN) avant l'injection du traitement antalgique et à 30 min de la première injection du médicament à l'étude.<br><br>***<u>Critère(s) de jugement secondaire(s) :</u>***<br>1/ Différence entre le score de l'Echelle Verbale Numérique (EVN) avant l'injection du traitement antalgique et à 30 min de la première injection du médicament à l'étude.<br>2/ Évaluation de la douleur par l'EVN à 10, 20, 30, 45 et à 60 minutes de la première injection du médicament antalgique.<br>3/ Estimation de la dose cumulée de morphine dans les deux bras en dose poids (mg/kg) durant les 30 premières minutes d'administration des traitements à l'étude.<br>4/ Proportion de patients ayant un score de l'échelle verbale numérique inférieur ou égale à 3 à 30 min après l'injection du médicament antalgique.<br>5/ Fréquence et intensité des effets indésirables évalués et enregistrés par le médecin.<br>6/ Nombre de prescription de molécules antalgiques de secours si le patient est en échec de l'analgésie (échelle verbale numérique d'évaluation de la douleur supérieure ou égale à 5) à 30, 45 ou 60 minutes.<br>7/ Mesure des signes vitaux (fréquence cardiaque, fréquence respiratoire, pression artérielle, score de Glasgow) avant l'injection du médicament antalgique et ensuite à 10, 20, 30, 45 et 60 minutes.<br>8/ Distribution de l'EVN a 10, 20, 30, 45 et à 60 min : Paramètres de tendance centrale (moyenne et médiane), paramètres de dispersion (écart type, intervalle interquartiles et valeurs extrêmes), normalité de la distribution.
**Traitement à l'étude**	Morphine Intraveineuse + Placebo
**Traitement comparateur/ placebo)**	Morphine Intraveineuse associée au Paracétamol
**Critères principaux de sélection, d'inclusion, de non-inclusion et d'exclusion**	***<u>Critères d'inclusion :</u>***<br>- Patient âgé d'au moins 18 ans rapportant une douleur évaluée par l'échelle verbale numérique supérieure ou égale à 5.<br>- Patient conscient (score de Glasgow = 15) pouvant parler et donner une évaluation verbale de sa douleur avec l'échelle verbale numérique<br>- Stabilité clinique (pression artérielle systolique >90 ou >180 mmHg, fréquence cardiaque >50 ou <150/min, et fréquence respiratoire >10 ou <30/min).<br>- Patient majeur, hors contexte de tutelle et/ou curatelle, affilié au Régime de la Sécurité Sociale.

	<u>Critères de non inclusion :</u><br>- Femmes enceinte ou allaitante.<br>- Patients avec un poids strictement inférieur à 50kg.<br>- Patients nécessitant la réalisation d'un geste douloureux dans le cadre de la prise en charge d'une douleur traumatique (réalignement d'une fracture par exemple).<br>- Œdème aigu pulmonaire, insuffisance respiratoire décompensée. Syndrome coronarien aigu ou cardiopathie ischémique non équilibrée en cours.<br>- Intoxication alcoolique ou à une drogue illicite présumée.<br>- Patient ayant déjà bénéficié d'une administration de morphine ou de paracétamol pour l'épisode douloureux aigu en cours.<br>- Pas de possibilité d'avoir un accès veineux.<br>- Allergie connue au paracétamol ou à la morphine.<br>- Antécédents connus d'insuffisance rénale ou hépatique.<br>- Antécédents de douleurs chroniques en cours de traitement.<br>- Associations avec la buprénorphine, la nalbuphine et la pentazocine.
**Calendrier des différentes visites et des différents examens**	V0 : Sélection des patients et inclusion<br>V1 : Randomisation des patients.<br>V2 : Suivis des patients à T0, T20, T30, T45 et T60min<br>V3 : Visite de fin d'étude à la fin de la prise en charge aux urgences.
**Analyse statistiques**	L'analyse du critère de jugement principal sera conduite en 2 temps : per protocole avec une hypothèse de non-infériorité du groupe intervention puis en intention de traiter.<br><br>La non infériorité du groupe morphine seule sera définie si les deux bornes inférieures des intervalles de confiance unilatéral à 97.5% de la variation de score mesuré par l'EVN entre l'inclusion et 30min dans chaque des deux groupes (douleurs traumatiques et non traumatiques) seront supérieures aux deux moyennes du même critère dans les deux mêmes groupes pour les patients ayant reçu l'association morphine + paracétamol auquel nous soustrairons la marge de non infériorité fixée par les experts à 1 point.

ANNEXE 4: ECHELLE VERBALE NUMERIQUE

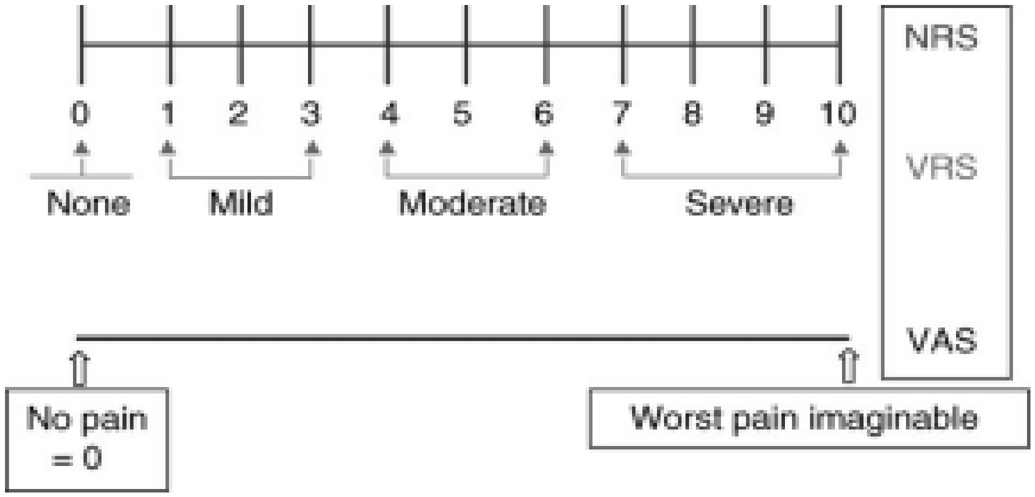

L’échelle verbale numérique (EVN ou échelle numérique : EN) mesure la perception de l’intensité douloureuse avec une échelle de 11 points, codée entre 0 et 10, où 0 représente l’absence de douleur et 10 représente la pire douleur imaginable. L’EVA et l’EN ont été validés dans des études précédentes (Todd et al. 1996; MacIntyre et al. 2010; Bijur et al., 2001 et 2003; Holdgate et al., 2003). Bijur et al. ont démontré la bonne reproductibilité de l’EVA en médecine d’urgence. En effet, la variation moyenne entre deux mesures effectuées à une minute d’intervalle en dehors de tout traitement était de 2 à 9 mm et ce quel que soit le niveau de l’intensité douloureuse [Bijur et al. 2001]. La même équipe a montré ensuite qu’il existait une grande concordance entre l’EN et l’EVA (Bijur et al. 2003).

L’EN est donc classiquement utilisée dans prise en charge de la douleur aiguë en préhospitalier et est recommandée dans les recommandations formalisées d’experts de la SFMU : « Il faut réévaluer l’intensité de la douleur pour apprécier l’efficacité des thérapeutiques et utiliser des échelles d’autoévaluation chez l’adulte communicant. L’échelle visuelle analogique (EVA) et l’échelle numérique (EN) sont les outils de référence » (Vivien et al. 2011). En médecine d’urgence, ces échelles ont des taux de faisabilité de 83 à 94 % (Riccard-Hibon et al. 1999; Blettery et al. 1996; Berthier et al. 1998).

Annexe 5 : Score de Ramsay

Echelle de Ramsay	
NIVEAU	REPONSE
1	Malade anxieux, agité.
2	Malade coopérant, orienté et calme.
3	Malade répondant aux ordres.
4	Malade endormi mais avec une réponse nette à la stimulation de la glabelle ou à un bruit intense
5	Malade endormi répondant faiblement aux stimulations ci-dessus.
6	Pas de réponse aux stimulations nociceptives.

Sédation optimale légère = score 2 à 3,
Sédation profonde = score 4 à 5,
Surdosage fréquent = score 6.

ANNEXE 6 : RCP CHLORURE DE SODIUM

RÉSUMÉ DES CARACTÉRISTIQUES DU PRODUIT

ANSM - Mis à jour le : 02/01/2018

1. 1. DENOMINATION DU MEDICAMENT

CHLORURE DE SODIUM FRESENIUS 0,9 %, solution injectable

2. 2. COMPOSITION QUALITATIVE ET QUANTITATIVE

Chlorure de sodium.. 0,9 g
Pour 100 ml de solution injectable.
Sodium.. 15,4 mmol/100 ml
Chlorures.. 15,4 mmol/100 ml
Osmolarité .... 308 mOsm/l
Osmolalité.. 290 mOsm/kg
pH compris entre 4,5 et 7
Pour la liste complète des excipients, voir rubrique 6.1.

3. 3. FORME PHARMACEUTIQUE

Solution injectable.

4. 4. DONNEES CLINIQUES

4.1. Indications thérapeutiques

5. • Rééquilibration ionique par apport de chlorure et de sodium.
6. • Déshydratations extra-cellulaires.
7. • Véhicule pour apport thérapeutique.
8. • Hypovolémie

4.2. Posologie et mode d'administration

Posologie

Les doses peuvent être exprimées en mEq ou mmol de sodium, masse de sodium ou masse de sel de sodium (1 g NaCl = 394 mg de Na, 17,1 mEq ou 17,1 mmol de Na et Cl).
La posologie, le débit et la durée d'administration doivent être déterminés en fonction de plusieurs facteurs comprenant l'âge, le poids, l'état clinique, le traitement concomitant et en particulier l'état d'hydratation du patient, des besoins en ions sodium et chlorure, la réponse clinique et biologique au traitement. L'équilibre hydrique et les concentrations électrolytiques sériques doivent être surveillés attentivement.
Pour le traitement de la déshydratation extracellulaire isotonique et de la déplétion sodique la posologie recommandée est:

9. • *Adultes* : 500 ml à 3000 ml /24 h.
10. • *Population pédiatrique* : 20 à 100 ml par 24 h et par kg de poids corporel. La posologie, le débit et le volume de perfusion dépendent de l'âge, du poids, du statut clinique et métabolique du patient et du traitement concomitant. La solution doit être administrée par un personnel expérimenté.

Lorsque la solution de CHLORURE DE SODIUM FRESENIUS 0,9 % est utilisée comme véhicule ou diluant pour préparation injectable d'autres médicaments, la posologie et le débit de perfusion seront principalement fonction de la nature et de la posologie du médicament à administrer.

Mode d'administration

Perfusion intraveineuse, injection intramusculaire ou sous-cutanée
Lorsque la solution est utilisée pour la dilution et l'administration de médicaments complémentaires, les instructions d'utilisation des substances ajoutées détermineront les volumes appropriés, les modes et voies d'administration pour chaque traitement.

4.3. Contre-indications

11. • Hyperchlorémie
12. • Hypernatrémie
13. • Cas sévères d'inflation hydrique et de rétention hydro-sodée particulièrement en cas d'insuffisance cardiaque décompensée, d'insuffisance hépatique décompensée (insuffisance œdémato-ascitique des cirrhoses), de prééclampsie / éclampsie.

Les contre-indications relatives au(x) médicament(s) ajouté(s) doivent être prises en compte.

4.4. Mises en garde spéciales et précautions d'emploi

CHLORURE DE SODIUM FRESENIUS 0,9 % est une solution isotonique.
La rééquilibration en sodium ne doit pas être effectuée à un rythme trop rapide, en particulier en raison d'un risque de complications neurologiques graves, tel que le syndrome de démyélinisation osmotique (voir rubriques 4.2 et 4.9).
En fonction du volume et du débit de perfusion, l'administration intraveineuse de chlorure de sodium peut provoquer :

14. • une surcharge hydrique et/ou en soluté conduisant à une hyperhydratation/hypervolémie (ex., états congestifs, notamment œdème périphérique)
15. • des perturbations électrolytiques cliniquement significatives et un déséquilibre acido-basique.

Utiliser ce médicament avec précaution chez les patients atteints d'hypertension, d'insuffisance cardiaque, d'insuffisance hépatocellulaire avec œdème et ascite, de cirrhose du foie, d'œdème périphérique ou pulmonaire, de fonction rénale altérée, d'obstruction du tractus urinaire, d'acidose métabolique, de pré-éclampsie, d'hyperaldostéronisme, d'hypervolémie, d'hypoprotéinémie ou d'autres affections et traitements (ex., corticostéroïdes) associés à une rétention hydrosodée (voir rubrique 4.5).
Ce traitement doit être effectué sous surveillance médicale stricte, la posologie devant être adaptée selon les modifications hydroélectrolytiques, en particulier les ions sodium et chlorure. L'administration doit être réalisée sous surveillance régulière et attentive.

Extravasation

Le site du cathéter doit être régulièrement contrôlé pour détecter les signes d'extravasation. En cas d'extravasation, l'administration doit être interrompue immédiatement, tout en maintenant en place la canule ou le cathéter inséré pour une prise en charge immédiate du patient. Si possible, une aspiration doit être pratiquée à travers la canule/le cathéter inséré afin de réduire la quantité de liquide présent dans les tissus avant de retirer la canule/le cathéter. Si une extrémité est atteinte, le membre concerné doit être surélevé.
Selon le produit extravasé (y compris les produits mélangés avec CHLORURE DE SODIUM FRESENIUS 0,9 % le cas échéant) et le stade/l'étendue des lésions éventuelles, des mesures spécifiques appropriées doivent être prises. Les options thérapeutiques peuvent inclure des interventions non pharmacologiques, pharmacologiques et/ou chirurgicales. En cas de dégradation de

la zone affectée (douleur continue, nécrose, ulcération), un chirurgien plasticien doit être consulté immédiatement (voir rubrique 4.8).
Le site d'extravasation doit être contrôlé au moins toutes les 4 heures pendant les premières 24 heures, puis une fois par jour.

Risque d'embolie gazeuse (voir rubrique 6.6)

16. • Ne pas utiliser les poches plastiques pour des connexions en série. Cette utilisation pourrait entraîner une embolie gazeuse en raison de l'aspiration de l'air résiduel de la première poche avant la fin de l'administration de solution venant de la deuxième poche.
17. • L'exercice d'une pression sur le récipient en plastique flexible contenant la solution intraveineuse pour augmenter le débit peut entraîner une embolie gazeuse si l'air résiduel contenu dans le récipient n'est pas complètement évacué avant l'administration.
18. • L'utilisation d'un set d'administration par voie intraveineuse avec une prise d'air en position ouverte pourrait entraîner une embolie gazeuse. Les sets d'administration par voie intraveineuse avec une prise d'air en position ouverte ne doivent pas être utilisés avec des récipients en plastique souple.

Utilisation gériatrique

Lors de la sélection du type de solution de perfusion et du volume/débit de perfusion pour un patient âgé, il est nécessaire de prendre en considération la susceptibilité de ces patients à présenter des maladies cardiaques, rénales, hépatiques ou autres, ainsi que leurs traitements médicamenteux concomitants.

Population pédiatrique

Chez les nouveau-nés, les nourrissons et les enfants, l'administration du produit nécessite une surveillance accrue.
Chez le nouveau-né et le prématuré, il peut exister une rétention de sodium en excès due à une fonction rénale immature. Chez ces patients, les perfusions répétées de chlorure de sodium doivent être réalisées uniquement après la détermination des concentrations plasmatiques en sodium.
Le dispositif de perfusion intraveineuse et le matériel d'administration doivent être contrôlés régulièrement.
En cas d'ajout de médicament, vérifier la compatibilité, la limpidité et la couleur avant usage (voir rubrique 6.2).
Ne pas conserver le mélange (voir rubrique 6.6).

4.5. Interactions avec d'autres médicaments et autres formes d'interactions

Associations faisant l'objet de précautions d'emploi :

19. • **Lithium**

L'administration de CHLORURE DE SODIUM FRESENIUS 0,9 % peut entraîner une diminution des concentrations en lithium et donc un risque de baisse de l'efficacité du lithium. En effet, la clairance rénale du sodium et du lithium peut être augmentée lors de l'administration de CHLORURE DE SODIUM FRESENIUS 0,9. La prudence est donc recommandée chez les patients qui ont un traitement au lithium.

20. • **Corticostéroïdes**

Les corticostéroïdes sont associés à une rétention hydrosodée (avec œdème et hypertension). Voir rubrique 4.4.

4.6. Fertilité, grossesse et allaitement

Ce produit peut être utilisé pendant la grossesse ou l'allaitement, si nécessaire. Les risques et les bénéfices pour chaque patiente doivent être attentivement considérés avant d'administrer la solution de CHLORURE DE SODIUM.
La prudence est recommandée chez les patientes souffrant de pré-éclampsie (voir rubriques 4.3 et 4.4).
Lorsqu'un médicament est ajouté, la nature du médicament et son utilisation pendant la grossesse et l'allaitement doivent être évaluées séparément.

4.7. Effets sur l'aptitude à conduire des véhicules et à utiliser des machines

Il n'existe pas d'information sur les effets du CHLORURE DE SODIUM FRESENIUS 0,9 % sur l'aptitude à conduire des véhicules et à utiliser des machines.

4.8. Effets indésirables

Les effets indésirables pouvant survenir chez des patients traités avec CHLORURE DE SODIUM FRESENIUS 0,9 % par perfusion intraveineuse sont indiqués ci-dessous.
Les effets indésirables listés dans cette rubrique sont mentionnés selon la convention suivante : très fréquent (≥ 1/10), fréquent (≥ 1/100 et < 1/10), peu fréquent (≥ 1/1000 et < 1/100), rare (≥ 1/10000 et < 1/1000), très rare (< 1/10000) et fréquence indéterminée (ne peut être estimée sur la base des données disponibles).

Classe de systèmes d'organe (SOC)	Effet indésirable (Terme MedDRA)	Fréquence
**Affections du système nerveux**	Tremblements	Indéterminée
**Troubles du métabolisme et de la nutrition**	Hypervolémie<br>Hypernatrémie<br>Acidose métabolique hyperchlorémique	Indéterminée
**Affections de la peau et du tissu sous-cutané**	Urticaire<br>Eruption cutanée<br>Prurit	Indéterminée
**Affections vasculaires**	Thrombose veineuse<br>Thrombophlébite<br>Hypotension	Indéterminée
**Troubles généraux et anomalies au site d'administration**	Frissons<br>Fièvre<br>Infection au niveau site de perfusion<br>Irritation au site de perfusion<br>Extravasation<br>Réaction locale<br>Douleur localisée*<br>Nécrose / ulcère*	Indéterminée

* Effets indésirables pouvant être notamment associés à une extravasation
Lorsque CHLORURE DE SODIUM FRESENIUS 0,9 % est utilisé comme véhicule ou diluant pour des préparations injectables d'autres médicaments, d'autres effets indésirables au(x) médicament(s) ajouté(s) à la solution peuvent survenir.

Déclaration des effets indésirables suspectés

La déclaration des effets indésirables suspectés après autorisation du médicament est importante. Elle permet une surveillance continue du rapport bénéfice/risque du médicament. Les professionnels de santé déclarent tout effet indésirable suspecté via le système national de déclaration : Agence nationale de sécurité du médicament et des produits de santé (ANSM) et réseau des Centres Régionaux de Pharmacovigilance - Site internet: www.ansm.sante.fr.

4.9. Surdosage

Symptômes du surdosage en chlorure de sodium

Une administration excessive de CHLORURE DE SODIUM FRESENIUS 0,9 % peut conduire à une hypernatrémie entraînant une déshydratation intracellulaire, qui doit être traitée en milieu spécialisé.
Les effets indésirables généraux de l'excès de sodium comprennent nausées, vomissements, diarrhée, crampes abdominales, soif, diminution de la sécrétion de salive et de larmes, sudation, fièvre, tachycardie, hypertension, insuffisance rénale, œdème cérébral, œdème pulmonaire et périphérique, arrêt respiratoire, céphalées, étourdissement, impatiences, irritabilité, lipothymie, contraction et raideur musculaire, convulsions, coma et décès.
Les signes cliniques du syndrome de démyélinisation osmotique sont progressifs : confusion, dysarthrie, dysphagie, faiblesse des membres, puis tétraplégie, délire et finalement coma. Les symptômes cliniques surviennent plusieurs jours après une correction trop rapide et/ou trop importante de l'hyponatrémie (voir rubriques 4.2 et 4.4).
Les chlorures en excès dans l'organisme peuvent provoquer une perte de bicarbonate avec une acidose.

Traitement

La première mesure à prendre est la réduction de la vitesse de perfusion ou l'arrêt de la perfusion.
Le traitement consiste en la surveillance de la natrémie et en l'administration de solution pour perfusion de glucose. En cas de convulsions le diazépam pourra être administré.
Lorsque le CHLORURE DE SODIUM FRESENIUS 0,9 % est utilisé comme véhicule ou diluant pour des préparations injectables d'autres médicaments, des signes et symptômes d'une perfusion excessive peuvent provenir du médicament ajouté. En cas de perfusion excessive accidentelle, interrompre le traitement et observer chez le patient toute apparition des signes et symptômes cliniques liés au médicament administré. Instaurer un traitement symptomatique et de soutien adapté, en fonction des besoins.

21. 5. PROPRIETES PHARMACOLOGIQUES

5.1. Propriétés pharmacodynamiques

Classe pharmacothérapeutique : Substituts du plasma et solutions pour perfusion/ solution d'électrolytes / chlorure de sodium code ATC : B05XA03.
(B: sang et organes hématopoïétiques)
CHLORURE DE SODIUM FRESENIUS 0,9 % est une solution isotonique dont l'osmolarité est d'environ 308 mOsm/l.
Les propriétés pharmacodynamiques de la solution sont celles des ions sodium et chlorure, qui maintiennent l'équilibre hydroélectrolytique.
Les ions tels que le sodium circulent à travers la membrane cellulaire, en utilisant des mécanismes de transport variés, parmi lesquels la pompe à sodium (Na^+, K^+-ATPase). Le sodium joue un rôle important dans la neurotransmission et l'électrophysiologie cardiaque, ainsi que dans le métabolisme rénal.
En cas d'ajout de médicament, la pharmacodynamie de la préparation dépendra aussi du médicament ajouté.

5.2. Propriétés pharmacocinétiques

Absorption

Étant donné que le chlorure de sodium est administré par voie intraveineuse, son absorption est complète, soit de 100 %.

Élimination

Les ions sodium et chlorure sont excrétés principalement dans les urines. De faibles quantités de sodium sont éliminées dans les fèces et la sueur.

En cas d’ajout de médicament, la pharmacocinétique de la préparation dépendra aussi du médicament ajouté.

5.3. Données de sécurité préclinique

Pas de données particulières, le chlorure de sodium étant un composant physiologique du plasma à la fois chez l'animal et chez l'Homme.
Les données de sécurité de l'additif doivent être considérées séparément.

22. 6. DONNEES PHARMACEUTIQUES

6.1. Liste des excipients

Eau pour préparations injectables.

6.2. Incompatibilités

Comme avec toutes les solutions parentérales, la compatibilité des médicaments avec la solution doit être vérifiée avant ajout.
L'incompatibilité du médicament vis-à-vis de la solution de CHLORURE DE SODIUM FRESENIUS 0,9 % doit être déterminée en contrôlant un éventuel changement de couleur et/ou une éventuelle formation de précipité, de complexe insoluble ou de cristaux. Se référer également à la notice accompagnant le médicament à ajouter.
En cas d'ajout de médicament, vérifier si la zone de pH, pour laquelle il est efficace, correspond à celle de la solution de CHLORURE DE SODIUM FRESENIUS 0,9 %.
Lorsqu'un médicament est ajouté à la solution de CHLORURE DE SODIUM FRESENIUS 0,9 %, le mélange doit être administré immédiatement.
Les médicaments connus pour être incompatibles ne doivent pas être utilisés.

6.3. Durée de conservation

Ampoule (verre): 5 ans.
Flacon (verre): 5 ans.
Flacon (polyéthylène): 2 ans
Poche Biluer: 14 mois
Poche B-Flex: 24 mois
Poche Careflex (polyoléfine) : 3 ans
Poche Perfuflex de 50 ml et 60 ml: 1 an
Poche Perfuflex de 100 ml et 120 ml: 18 mois
Poche Perfuflex de 250 ml, 300 ml, 500 ml, 1 000 ml, 2 000 ml et 4500 ml: 2 ans
Ampoule (polypropylène): 3 ans.

6.4. Précautions particulières de conservation

Poches: à conserver à une température ne dépassant pas 25°C.
Flacons et poches Careflex: pas de précautions particulières de conservation.

6.5. Nature et contenu de l'emballage extérieur

Ampoule de 2 ml, 5 ml, 10 ml ou 20 ml en verre incolore de type I, munie de deux pointes autocassables.
Flacon de 125 ml, 250 ml, 500 ml ou 1000 ml en verre incolore de type II, fermé par un bouchon en caoutchouc chlorobutyl.

Flacon de 125 ml en verre incolore de type II, fermé par un bouchon en caoutchouc chlorobutyl, rempli à 50 ml, 60 ml, 100 ml.
Flacon de 250 ml en verre incolore de type II, fermé par un bouchon en caoutchouc chlorobutyl, rempli à 125 ml.
Flacon en polyéthylène de 100 ml rempli à 50 ml, de 100 ml, 250 ml, 500 ml ou 1000 ml.
Poche souple type Biluer de 300 ml, 500 ml, 1000 ml, 2000 ml, 3000 ml ou 4500 ml en polychlorure de vinyle (PVC) plastifié, munie de deux tubulures en PVC obturées par des embouts en polycarbonate.
Poche souple type Biluer de 500 ml en polychlorure de vinyle (PVC) plastifié, munie de deux tubulures en PVC obturées par des embouts en polycarbonate remplie à 300 ml.
Poche souple type B-Flex de 2000 ml, 3000 ml ou 4500 ml en polychlorure de vinyle (PVC) plastifié, munie d'une tubulure en PVC obturée par un embout composé d'un corps en polycarbonate et d'un bouchon en bromobutyl.
Poche Careflex (polyoléfine) de 2000 ml ou 3000 ml.
Poche souple type Perfuflex de 50 ml, 60 ml, 100 ml, 120 ml, 250 ml, 300 ml, 500 ml, 1000 ml, 2000 ml ou 4500 ml en polychlorure de vinyle (PVC) plastifié, munie d'un twist-off en PVC et d'une tubulure en PVC obturée par un embout en polycarbonate, d'un bouchon en latex et éventuellement d'un disque en PVC.
Poche souple type Perfuflex de 150 ml en polychlorure de vinyle (PVC) plastifié, munie d'un twist-off en PVC et d'une tubulure en PVC obturée par un embout en polycarbonate, d'un bouchon en latex et éventuellement d'un disque en PVC, remplie à 50 ml, 100 ml.
Poche souple type Perfuflex de 300 ml en polychlorure de vinyle (PVC) plastifié, munie d'un twist-off en PVC et d'une tubulure en PVC obturée par un embout en polycarbonate, d'un bouchon en latex et éventuellement d'un disque en PVC, remplie à 250 ml.
Ampoule (polypropylène) de 10 ml ou 20 ml.
Toutes les présentations peuvent ne pas être commercialisées.

6.6. Précautions particulières d’élimination et de manipulation

Ne pas utiliser si l'emballage /la poche/ l'ampoule/ le flacon est endommagé(e).
Ne pas réutiliser : usage unique.
Eliminer tout(e) poche/ampoule/flacon partiellement utilisé(e).
Ne pas reconnecter une poche partiellement utilisée.
En cas d’ajout de médicament, bien mélanger la solution avant utilisation.
La solution doit être inspectée visuellement afin de détecter toute particule, tout dommage de la poche/ de l’ampoule/ du flacon et tout signe visible de détérioration avant administration.
En cas d’ajout de médicament, la solution doit être administrée avec un matériel stérile, apyrogène et en utilisant une technique aseptique. Le matériel doit être amorcé à l'aide de la solution pour éviter toute introduction d'air dans le système.
Tout produit non utilisé ou déchet doit être éliminé conformément à la réglementation en vigueur.
Ne pas utiliser les poches plastiques pour des connexions en série. Cette utilisation pourrait entraîner une embolie gazeuse en raison de l'aspiration de l'air résiduel de la première poche avant la fin de l'administration de solution venant de la deuxième poche.
L’exercice d’une pression sur le récipient en plastique flexible contenant la solution intraveineuse pour augmenter le débit peut entraîner une embolie gazeuse si l’air résiduel contenu dans le récipient n’est pas complètement évacué avant l’administration.
L’utilisation d’un set d’administration par voie intraveineuse avec une prise d’air en position ouverte pourrait entraîner une embolie gazeuse. Les sets d’administration par voie intraveineuse avec une prise d’air en position ouverte ne doivent pas être utilisés avec des récipients en plastique souple.

23. 7. TITULAIRE DE L’AUTORISATION DE MISE SUR LE MARCHE

FRESENIUS KABI FRANCE SA
5, PLACE DU MARIVEL
92316 SEVRES CEDEX

24. 8. NUMERO(S) D’AUTORISATION DE MISE SUR LE MARCHE

25. • 34009 560 775 8 8: 2 ml en ampoule (verre), boîte de 50.
26. • 34009 560 776 4 9: 5 ml en ampoule (verre), boîte de 50.
27. • 34009 560 777 0 0: 10 ml en ampoule (verre), boîte de 50.
28. • 34009 552 720 3 8: 20 ml en ampoule (verre), boîte de 50.

29. • 34009 560 778 7 8: 2 ml en ampoule (verre), boîte de 100.
30. • 34009 552 718 9 5: 5 ml en ampoule (verre), boîte de 100.
31. • 34009 554 056 3 4: 10 ml en ampoule (verre), boîte de 100.
32. • 34009 554 478 5 6: 50 ml en flacon de 125 ml (verre).
33. • 34009 554 477 9 5: 60 ml en flacon de 125 ml (verre).
34. • 34009 554 476 2 7: 100 ml en flacon de 125 ml (verre).

ANNEXE 7: RCP PARACETAMOL

RÉSUMÉ DES CARACTÉRISTIQUES DU PRODUIT

ANSM - Mis à jour le : 31/08/2016

1. DENOMINATION DU MEDICAMENT

PARACETAMOL KABI 10 mg/ml, solution pour perfusion

2. COMPOSITION QUALITATIVE ET QUANTITATIVE

Paracétamol .... 10,00 mg
Pour 1 mL
Un flacon de 100 mL contient 1000 mg de paracétamol.
Une poche de 100 mL contient 1000 mg de paracétamol.
Un flacon de 50 mL contient 500 mg de paracétamol.
Une poche de 50 mL contient 500 mg de paracétamol.
Pour la liste complète des excipients, voir rubrique 6.1

3. FORME PHARMACEUTIQUE

Solution pour perfusion.
Solution claire légèrement jaune.

4. DONNEES CLINIQUES

4.1. Indications thérapeutiques

PARACETAMOL KABI est indiqué dans le traitement de courte durée des douleurs d'intensité modérée, en particulier en période post-opératoire et dans le traitement de courte durée de la fièvre, lorsque la voie intraveineuse est cliniquement justifiée par l'urgence de traiter la douleur ou l'hyperthermie et/ou lorsque d'autres voies d'administration ne sont pas possibles.

4.2. Posologie et mode d'administration

Voie intraveineuse.
Flacon et poche de **100 mL** : réservé à l'adulte, à l'adolescent et à l'enfant de **plus de 33 kg**.
Flacon et poche de **50 mL** : réservé aux nouveau-nés, aux nourrissons et à l'enfant de **moins de 33 kg**.

Posologie:

La dose à administrer et la taille du flacon à utiliser dépendent uniquement du poids du patient. Le volume administré ne doit pas dépasser la dose établie. Le cas échéant, il faut diluer le volume souhaité dans une solution pour perfusion appropriée avant administration (voir rubrique 6.6) ou utiliser un pousse seringue :

**Flacon et poche de 50 mL**				
**Poids du patient**	**Dose par prise**	**Volume par prise**	**Volume maximal de Paracétamol Kabi 10 mg/ml par prise basé sur les limites de poids supérieures (mL)*** **	**Dose journalière maximale****
≤ 10 kg*	7,5 mg/kg	0,75 mL/kg	7,5 mL	30 mg/kg
> 10 kg et ≤ 33 kg	15 mg/kg	1,5 mL/kg	49,5 mL	60 mg/kg sans dépasser 2 g
**Flacon et poche de 100 mL**				
**Poids du patient**	**Dose par prise**	**Volume par prise**	**Volume maximal de Paracétamol Kabi 10 mg/ml par prise basé sur les limites de poids supérieures (mL)*** **	**Dose journalière maximale****
> 33 kg et ≤ 50 kg	15 mg/kg	1,5 mL/kg	75 mL	60 mg/kg sans dépasser 3 g
> 50 kg avec facteurs de risque additionnels d'hépatotoxicité	1 g	100 mL	100 mL	3 g
> 50 kg sans facteurs de risque additionnels d'hépatotoxicité	1 g	100 mL	100 mL	4 g

*** Nouveau-né prématuré** : aucune donnée de sécurité et d'efficacité n'est disponible chez les nouveau-nés prématurés (voir section 5.2)

**** Dose journalière maximale** : la dose journalière maximale présentée dans les tableaux ci-dessus est valable pour les patients ne recevant pas d'autres médicaments contenant du paracétamol et doit être ajustée si besoin en tenant compte de l'apport de tels médicaments

***** Les patients de plus faible poids nécessiteront des volumes plus faibles**

L'intervalle minimal entre deux administrations doit être d'au moins 4 heures.

L'intervalle minimal entre deux prises chez les patients insuffisants rénaux sévères (clairance de la créatinine ≤ 30 mL/min) doit être d'au moins 6 heures.

Chez le patient adulte, en cas d'insuffisance hépatocellulaire, d'alcoolisme chronique, de malnutrition chronique (réserves basses en glutathion hépatique) ou de déshydratation, la dose maximale journalière ne doit pas dépasser 3 g (voir rubrique 4.4).

Insuffisance rénale sévère

Il est recommandé d'augmenter l'intervalle entre 2 administrations à 6 heures au moins lors de l'administration du paracétamol chez l'insuffisant rénal sévère (clairance de la créatinine ≤ 30 mL/min) (voir rubrique 5.2).

Mode d'administration

RISQUE D'ERREURS MEDICAMENTEUSES

Faites attention lorsque vous prescrivez ou administrez PARACETAMOL KABI 10 mg/ml afin d'éviter les erreurs de dose administrée dues à la confusion entre milligrammes (mg) et millilitres (mL), qui pourraient conduire à un surdosage accidentel et à un décès.

Assurez-vous que la bonne dose est communiquée et administrée. Lors de la prescription, il est recommandé d'indiquer la dose en mg et le volume correspondant en mL. Assurez-vous que la dose est précisément mesurée et administrée.

La solution de paracétamol est administrée en perfusion intraveineuse de 15 minutes.

Patients de poids ≤ 10 kg :

- Le flacon/la poche de PARACETAMOL KABI 10 mg/ml ne doit pas être suspendu comme une perfusion en raison du faible volume de médicament à administrer à ces patients.

- Le volume à administrer doit être prélevé du flacon/de la poche et peut être administré en l'état ou dilué (dans un volume allant de 1 à 9) dans une solution de chlorure de sodium à 0,9% ou de glucose à 5% et administré en 15 minutes

- Une seringue de 5 ou 10 mL peut être utilisée pour mesurer la dose appropriée au poids de l'enfant et le volume souhaité. **Cependant, le volume ne devra jamais dépasser 7,5 mL par dose.**

- L'utilisateur doit se référer à l'information produit pour les recommandations de posologies.

Pour les flacons de 50 mL :

- Le flacon de PARACETAMOL KABI 10 mg/ml peut éventuellement être dilué (dans un volume allant de 1 à 9) dans une solution de chlorure de sodium à 0,9% ou de glucose à 5%. Dans ce cas, la solution diluée doit être utilisée dans l'heure suivant la préparation (incluant le temps de perfusion).

- Pour prélever la solution, une aiguille de 0,8 mm (aiguille Gauge 21) doit être utilisée et le bouchon devra être perforé verticalement à l'endroit spécifiquement désigné.

- Comme pour toutes les solutions pour perfusion conditionnées dans des flacons en verre, il est rappelé qu'une surveillance étroite est particulièrement recommandée à la fin de la perfusion quelle que soit la voie d'administration. Cette surveillance à la fin de la perfusion s'applique tout particulièrement aux perfusions par voie centrale de façon à éviter une embolie gazeuse.

Pour les flacons de 100 mL :

- Pour prélever la solution, une aiguille de 0,8 mm (aiguille Gauge 21) doit être utilisée et le bouchon devra être perforé verticalement à l'endroit spécifiquement désigné.

- Comme pour toutes les solutions pour perfusion conditionnées dans des flacons en verre, il est rappelé qu'une surveillance étroite est particulièrement recommandée à la fin de la perfusion quelle que soit la

voie d'administration. Cette surveillance à la fin de la perfusion s'applique tout particulièrement aux perfusions par voie centrale de façon à éviter une embolie gazeuse.

4.3. Contre-indications

PARACETAMOL KABI est contre-indiqué :

- en cas d'hypersensibilité au paracétamol ou au chlorhydrate de propacétamol (prodrogue du paracétamol) ou à l'un des excipients,

RISQUE D'ERREURS MEDICAMENTEUSES

Faites attention aux erreurs de posologie dues à la confusion entre milligrammes (mg) et millilitres (mL) qui pourraient conduire à un surdosage accidentel et à un décès (voir rubrique 4.2).

Il est recommandé d'avoir recours à un traitement antalgique adapté *per os* dès que cette voie d'administration est possible.
Pour éviter un risque de surdosage, vérifier l'absence de paracétamol dans la composition d'autres médicaments associés.
Des doses supérieures à celles recommandées entraînent un risque d'atteinte hépatique très sévère. Les symptômes et les signes cliniques de l'atteinte hépatique (incluant hépatite fulminante, insuffisance hépatique, hépatite cholestatique, hépatite cytolytique) sont généralement observés après 2 jours et atteignent habituellement un maximum après 4 à 6 jours. Un traitement avec antidote doit être donné dès que possible (voir rubrique 4.9).

Précautions d'emploi

Le paracétamol est à utiliser avec précaution en cas :

- d'insuffisance hépato-cellulaire,
- d'insuffisance rénale sévère (clairance de la créatinine ≤ 30 ml/min (voir rubriques 4.2 et 5.2)),
- d'alcoolisme chronique,
- de malnutrition chronique (réserves basses en gluthation hépatique),
- de déshydratation.

4.5. Interactions avec d'autres médicaments et autres formes d'interactions

Le probénécide entraîne une diminution de près de la moitié de la clairance du paracétamol en inhibant sa conjugaison à l'acide glucuronique. Une diminution de la dose de paracétamol est à envisager en cas d'association au probénécide.
Le salicylamide peut allonger la demi-vie d'élimination du paracétamol.
Une attention particulière doit être exercée en cas de prise concomitante d'inducteurs enzymatiques (voir rubrique 4.9).
L'utilisation concomitante de paracétamol (4 g par jour pendant au moins 4 jours) et d'anticoagulants oraux peut conduire à de légères variations de l'INR. Dans ce cas, une surveillance accrue de l'INR est nécessaire pendant la période d'utilisation concomitante et 1 semaine après l'arrêt du paracétamol.

4.6. Fertilité, grossesse et allaitement

Grossesse

L'expérience clinique de l'administration intraveineuse de paracétamol est limitée. Cependant, les données épidémiologiques sur l'utilisation de doses thérapeutiques orales de paracétamol ne montrent aucun effet indésirable sur la grossesse ou sur la santé du fœtus ou du nouveau-né.
Des données prospectives dans des situations de grossesses exposées à des surdosages n'ont pas montré d'augmentation du risque de malformation.
Chez l'animal, les études sur la reproduction n'ont pas été effectuées avec la forme intraveineuse. Cependant, les études effectuées avec la voie orale n'ont pas mis en évidence d'effet malformatif ou fœtotoxique.
Néanmoins, PARACETAMOL KABI ne doit être utilisé pendant la grossesse qu'après une évaluation soigneuse du rapport bénéfice/risque. Dans ce cas, la posologie et la durée de traitement recommandées doivent être strictement respectées.

Allaitement

Après administration orale, le paracétamol passe en faible quantité dans le lait maternel. Aucun effet indésirable sur le nourrisson n'a été rapporté. En conséquence, PARACETAMOL KABI peut être utilisé pendant l'allaitement.

4.7. Effets sur l'aptitude à conduire des véhicules et à utiliser des machines

Sans objet.

4.8. Effets indésirables

Comme avec tous les médicaments contenant du paracétamol, les réactions secondaires sont rares (> 1/10 000, < 1/1000) ou très rares (< 1/10 000), elles sont décrites ci-dessous :

**Système**	**Rare**<br>**> 1/10 000, < 1/1000**	**Très rare**<br>**< 1/10 000**
Général	Malaise	Réaction d'hypersensibilité
Cardiovasculaire	Hypotension	
Hépatique	Elévation des transaminases hépatiques	
Plaquettes/sang		Thrombocytopénie<br>Leucopénie<br>Neutropénie

De très rares cas de réactions d'hypersensibilité allant du simple rash cutané ou urticaire au choc anaphylactique ont été rapportés et nécessitent l'arrêt du traitement. De très rares cas de réactions cutanées graves ont été rapportés.
Des cas d'érythème, de bouffées vasomotrices, de prurit et de tachycardie ont été rapportés.

<u>Déclaration des effets indésirables suspectés</u>

La déclaration des effets indésirables suspectés après autorisation du médicament est importante. Elle permet une surveillance continue du rapport bénéfice/risque du médicament. Les professionnels de santé déclarent tout effet indésirable suspecté via le système national de déclaration : Agence nationale de sécurité du médicament et des produits de santé (ANSM) et réseau des Centres Régionaux de Pharmacovigilance - Site internet : www.ansm.sante.fr.

4.9. Surdosage

Le risque d'atteinte hépatique (incluant hépatite fulminante, insuffisance hépatique, hépatite cholestatique, hépatite cytolytique) est particulièrement à craindre chez les sujets âgés, chez les jeunes enfants, chez les patients avec atteinte hépatique, en cas d'alcoolisme chronique, chez les

patients souffrant de malnutrition chronique, et chez les patients recevant des inducteurs enzymatiques. Dans ces cas, l'intoxication peut être mortelle.
Les symptômes apparaissent généralement dans les 24 premières heures et comprennent : nausées, vomissements, anorexie, pâleur et douleurs abdominales.
Un surdosage, à partir de 7,5 g de paracétamol en une seule prise chez l'adulte et 140 mg/kg de poids corporel en 1 seule prise chez l'enfant, provoque une cytolyse hépatique susceptible d'aboutir à une nécrose complète et irréversible se traduisant par une insuffisance hépato-cellulaire, une acidose métabolique, une encéphalopathie pouvant aller jusqu'au coma et à la mort.
Simultanément, on observe une augmentation des transaminases hépatiques (ASAT, ALAT), de la lactico-déshydrogénase, de la bilirubine, ainsi qu'une diminution du taux de prothrombine pouvant apparaître 12 à 48 heures après administration.
Les symptômes cliniques de l'atteinte hépatique sont généralement observés après deux jours, et atteignent un maximum après 4 à 6 jours.

Conduite d'urgence

- Hospitalisation immédiate.

- Avant de commencer le traitement, prélever un tube de sang pour faire le dosage plasmatique du paracétamol, dès que possible après le surdosage.

- Le traitement du surdosage comprend l'administration de l'antidote N-acétylcystéine (NAC) par voie intraveineuse ou voie orale, si possible avant la dixième heure. La NAC peut cependant apporter une certaine protection même après 10 heures, mais dans ce cas un traitement prolongé est donné.

- Traitement symptomatique.

- Des tests hépatiques doivent être effectués au début et répétés toutes les 24 heures.

- Habituellement les transaminases hépatiques se normalisent après une ou deux semaines avec récupération complète de la fonction hépatique. Cependant, dans les cas très sévères, une transplantation hépatique peut être nécessaire.

5. PROPRIETES PHARMACOLOGIQUES

5.1. Propriétés pharmacodynamiques

Classe pharmacothérapeutique : **AUTRES ANALGESIQUES ET ANTIPYRETIQUES,** code ATC : **N02BE01**
Le mécanisme précis des propriétés antalgiques et antipyrétiques du paracétamol reste à établir pouvant impliquer des actions centrales et périphériques.
PARACETAMOL KABI permet un début de soulagement de la douleur dans les 5 à 10 minutes suivant le début de l'administration. Le pic de l'effet antalgique est obtenu en 1 heure et la durée de cet effet est habituellement de 4 à 6 heures.
PARACETAMOL KABI réduit la fièvre dans les 30 minutes suivant le début de l'administration et la durée d'effet antipyrétique est d'au moins 6 heures.

5.2. Propriétés pharmacocinétiques

Adultes

Absorption

La pharmacocinétique du paracétamol est linéaire jusqu'à 2 g en dose unique et après administration répétée sur 24 h.

La biodisponibilité du paracétamol après perfusion de 500 mg et 1 g de PARACETAMOL KABI est similaire à celle observée après perfusion de 1 g et 2 g de propacétamol (contenant 500 mg et 1 g de paracétamol respectivement).
La concentration plasmatique maximale (C_{max}) de paracétamol observée à la fin des 15 minutes de perfusion intraveineuse de 500 mg et 1 g de PARACETAMOL KABI est de l'ordre de 15 µg/ml et 30 µg/ml respectivement.

Distribution

- Le volume de distribution du paracétamol est approximativement de 1 l/kg.

- La liaison aux protéines plasmatiques est faible.

- Après perfusion de 1 g de paracétamol, des concentrations significatives de paracétamol (de l'ordre de 1,5 µg/ml) ont été retrouvées dans le liquide céphalo-rachidien dès la 20ème minute après la perfusion.

Métabolisme

Le paracétamol est métabolisé essentiellement au niveau du foie selon 2 voies hépatiques majeures : la glycuroconjugaison et la sulfoconjugaison. Cette dernière voie est rapidement saturable aux posologies supérieures aux doses thérapeutiques. Une faible proportion (moins de 4%) est transformée par le cytochrome P 450 en un intermédiaire réactif (le N-acétyl benzoquinone imine) qui, dans les conditions normales d'utilisation, est rapidement détoxifié par le glutathion réduit et éliminé dans les urines après conjugaison à la cystéine et à l'acide mercaptopurique. En revanche, lors d'intoxications massives, la quantité de ce métabolite toxique est augmentée.

Elimination

L'élimination des métabolites du paracétamol est essentiellement urinaire. 90% de la dose administrée est excrétée dans les urines en 24 heures, principalement sous forme glycuroconjuguée (60 à 80%) et sulfoconjuguée (20 à 30%).
Moins de 5% est éliminé sous forme inchangée.
La demi-vie plasmatique est de 2,7 heures et la clairance corporelle totale est d'environ 18 l/h.

Nouveau-nés, nourrissons et enfants

Les paramètres pharmacocinétiques du paracétamol observés chez le nourrisson et l'enfant sont similaires à ceux obtenus chez l'adulte, à l'exception de la demi-vie plasmatique qui est légèrement plus courte (1,5 à 2 heures). Chez le nouveau-né, la demi-vie plasmatique est plus longue que chez le nourrisson, à savoir environ 3,5 heures. Le nouveau-né, le nourrisson et l'enfant jusqu'à 10 ans éliminent significativement moins de dérivés glycuroconjugués et plus de dérivés sulfoconjugués que l'adulte.

Tableau. Les valeurs pharmacocinétiques en fonction de l'âge (clairance standardisée$^{*}CL_{std}/F_{orale}$ ($l.h^{-1}$ 70 kg^{-1}) sont présentées ci -dessous.

Age	Poids (kg)	CL_{std}/F_{orale} ($l.h^{-1}$ 70 kg^{-1})
40 semaines d'aménorrhée	3.3	5.9
3 mois	6	8.8
6 mois	7.5	11.1
1 an	10	13.6
2 ans	12	15.6
5 ans	20	16.3
8 ans	25	16.3

$^{*}CL_{std}$ est l'estimation de la CL pour la population

Populations particulières

Insuffisant rénal

En cas d'insuffisance rénale sévère (clairance de la créatinine 10-30 ml/min), l'élimination du paracétamol est légèrement retardée, la demi-vie d'élimination variant de 2 à 5,3 h. La vitesse d'élimination des dérivés glycuro et sulfoconjugués est 3 fois plus lente chez l'insuffisant rénal sévère que chez le sujet sain.
En conséquence, il est recommandé de respecter un intervalle d'au moins 6 heures entre deux administrations chez l'insuffisant rénal sévère (clairance de la créatinine ≤ 30 ml/min) (voir rubrique 4.2).

Sujet âgé

La pharmacocinétique et le métabolisme du paracétamol ne sont pas modifiés chez le sujet âgé. Aucun ajustement de dose n'est requis dans cette population.

5.3. Données de sécurité préclinique

Les données précliniques n'indiquent aucun autre risque particulier en dehors de l'information déjà incluse dans les autres rubriques du RCP.
Des études de tolérance locale effectuées chez le rat et le lapin ont montré une bonne tolérance de PARACETAMOL KABI.
L'absence d'hypersensibilité retardée de contact a été testée chez le cobaye.

6. DONNEES PHARMACEUTIQUES

6.1. Liste des excipients

Cystéine, mannitol, eau pour préparations injectables.

6.2. Incompatibilités

En l’absence d’études de compatibilité, ce médicament ne doit pas être mélangé avec d’autres médicaments.

6.3. Durée de conservation

Flacon : 2 ans.
Poche : 2 ans.
D'un point de vue microbiologique, le produit doit être utilisé immédiatement.
Après dilution dans du chlorure de sodium à 0,9% ou de glucose à 5%, la solution doit être utilisée immédiatement ou dans l'heure qui suit sa préparation incluant le temps de perfusion.

6.4. Précautions particulières de conservation

Poche : A conserver à une température ne dépassant pas 25°C.
Ne pas mettre au réfrigérateur. Ne pas congeler.

6.5. Nature et contenu de l'emballage extérieur

100 mL et 50 mL en flacon (verre incolore de type II) avec bouchon en halobutyl et capsule Flip-Off en aluminium/plastique.
Boites de 1, 10, 12 ou 20 flacons.
100 mL et 50 mL en poche Freeflex (polypropylène/SIS-polypropylène/Styrène éthylène butadiène) avec suremballage en aluminium ou transparent/aluminium.
Boites de 20, 50 et 60 poches.

Toutes les présentations peuvent ne pas être commercialisées.

6.6. Précautions particulières d'élimination et de manipulation

Avant toute administration, le produit doit être visuellement contrôlé pour détecter toute particule et jaunissement. A usage unique. Toute solution non utilisée doit être éliminée.
La solution diluée doit être contrôlée visuellement et ne doit pas être utilisée en présence d'opalescence, de particules visibles ou de précipité.

7. TITULAIRE DE L'AUTORISATION DE MISE SUR LE MARCHE

FRESENIUS KABI FRANCE
5 place du Marivel
92316 SEVRES CEDEX

8. NUMERO(S) D'AUTORISATION DE MISE SUR LE MARCHE

- 34009 580 073 9 2 : 50 mL en flacon (verre) ; boite de 1.
- 34009 580 074 5 3 : 50 mL en flacon (verre) ; boite de 10.
- 34009 580 075 1 4 : 50 mL en flacon (verre) ; boite de 12.
- 34009 580 076 8 2 : 50 mL en flacon (verre) ; boite de 20.
- 34009 580 077 4 3 : 100 mL en flacon (verre) ; boite de 1.
- 34009 580 078 0 4 : 100 mL en flacon (verre) ; boite de 10.
- 34009 580 079 7 2 : 100 mL en flacon (verre) ; boite de 12.
- 34009 580 080 5 4 : 100 mL en flacon (verre) ; boite de 20.

ANNEXE 8 : MODELE DE RCP MORPHINE

35. RÉSUMÉ DES CARACTÉRISTIQUES DU PRODUIT

ANSM - Mis à jour le : 30/05/2017

36. 1. DENOMINATION DU MEDICAMENT

MORPHINE (CHLORHYDRATE) AGUETTANT 10 mg/mL, solution injectable

37. 2. COMPOSITION QUALITATIVE ET QUANTITATIVE

Chlorhydrate de morphine.. 10,00 mg
Pour 1 mL de solution injectable.
Une ampoule de 1 ml contient 10 mg de chlorhydrate de morphine.
Une ampoule de 5 ml contient 50 mg de chlorhydrate de morphine.
Une ampoule de 10 ml contient 100 mg de chlorhydrate de morphine.
Excipient(s) à effet notoire : sodium
Une ampoule de 1 mL contient 3,0 mg de sodium, équivalent à 0,13 mmol de sodium.
Une ampoule de 5 mL contient 14,8 mg de sodium, équivalent à 0,64 mmol de sodium.
Une ampoule de 10 mL contient 29,5 mg de sodium, équivalent à 1,28 mmol de sodium.
Pour la liste complète des excipients, voir rubrique 6.1.

38. 3. FORME PHARMACEUTIQUE

Solution injectable.

39. 4. DONNEES CLINIQUES

4.1. Indications thérapeutiques

Douleurs intenses et/ou rebelles aux antalgiques de niveau plus faible.

4.2. Posologie et mode d'administration

Posologie

La relation dose-efficacité-tolérance est très variable d'un patient à l'autre. Il est donc important d'évaluer fréquemment l'efficacité et la tolérance, et d'adapter la posologie progressivement en fonction des besoins du patient. Il n'y a pas de dose maximale, tant que les effets indésirables peuvent être contrôlés.
La voie intramusculaire n'est pas recommandée car elle est douloureuse et ne présente pas d'avantage cinétique par rapport à la voie sous-cutanée.
Les voies péridurale, intrathécale et intraventriculaire, nécessitent que :

- la morphine utilisée soit SANS CONSERVATEUR,

- la solution soit FILTREE AVANT L'INJECTION à travers un filtre de 0,22 µm (pour prévenir une éventuelle contamination particulaire suite à l'ouverture de l'ampoule).

Ordre d'équivalence des doses selon la voie d'administration, à titre indicatif :

Voie orale	Sous-cutanée	Intraveineuse	Péridurale	Intrathécale
1 mg	½ à 1/3 mg	½ à 1/3 mg	1/10 à 1/20 mg	1/50 à 1/200 mg

L'administration simultanée de morphine par deux voies d'administration différentes est à éviter, car elle expose à un risque de surdosage en raison des différences cinétiques entre les différentes voies d'administration.

Traitement des douleurs aiguës (notamment post-opératoires) :

Voies intra-veineuse et sous-cutanée:
Chez l'adulte, la morphine est le plus souvent administrée par voie intra-veineuse de manière fractionnée («par titration»), à la dose de 1 à 3 mg (en fonction du terrain, principalement de l'âge du patient), toutes les 10 min environ, jusqu'à obtention d'une analgésie satisfaisante (ou apparition d'effet indésirable) et avec surveillance continuelle du patient.
Si un traitement relais s'avère nécessaire, il peut être fait appel soit à des injections sous-cutanées de 5 à 10 mg toutes les 4 à 6 heures, soit à une analgésie autocontrôlée par voie intra-veineuse avec des bolus de 0,5 à 1 mg suivi d'une période sans injection possible («période réfractaire») d'environ 10 minutes.
La morphine en perfusion intra-veineuse (1 à 5 mg/h) est habituellement réservée à des patients en ventilation contrôlée en service de réanimation.
Population pédiatrique : La morphine est le plus souvent administrée par voie intra-veineuse de manière fractionnée («par titration»). Une dose initiale de 0,025 à 0,1 mg/kg (en fonction du terrain, principalement de l'âge du patient) est suivie, si besoin, de bolus d'environ 0,025 mg/kg toutes les 5 à 10 minutes, jusqu'à obtention d'une analgésie satisfaisante (ou apparition d'effet indésirable) et avec surveillance continuelle du patient.
Si un traitement relais s'avère nécessaire, il peut être fait appel à une perfusion intra-veineuse continue de 0,01 à 0,02 mg/kg/h sous surveillance en salle de réveil ou en soins intensifs.
L'analgésie autocontrôlée par voie intra-veineuse peut être réalisable à partir de 6 ans, les bolus sont de 0,015 à 0,02 mg/kg, suivis d'une période sans injection possible («période réfractaire») de 10 à 15 minutes. Il peut y être associé une dose continue de 0,005 à 0,02 mg/kg/h.
En raison de son caractère douloureux, la voie sous-cutanée n'est pas recommandée chez l'enfant.
Voie péridurale :
Chez l'adulte : 2 à 6 mg toutes les 12 à 24 heures.
Population pédiatrique : 0,03 à 0,05 mg/kg, à renouveler si besoin en fonction de la surveillance clinique après 12 à 24 heures.
Voie intrathécale
Chez l'adulte : 0,1 à 0,2 mg toutes les 12 à 24 heures.

Traitement des douleurs chroniques (notamment d'origine cancéreuse) :
Doses initiales en fonction de la voie d'administration
Rapportées au poids, les doses chez l'enfant et chez l'adulte sont équivalentes.
Voie sous-cutanée :
Chez les patients n'ayant pas de traitement préalable par de la morphine orale, la posologie initiale quotidienne sera de 0,5 mg/kg/j (classiquement 30 mg/j chez l'adulte), en perfusion continue de préférence (plutôt qu'en injections itératives toutes les quatre à six heures).
Chez les patients recevant auparavant de la morphine par voie orale, la posologie initiale quotidienne sera la moitié de la dose orale administrée. Si la posologie orale était insuffisante, il est possible de passer d'emblée à une posologie supérieure (Voir adaptation posologique).
Voie intraveineuse :
Chez les patients n'ayant pas de traitement préalable par de la morphine orale, la posologie initiale quotidienne sera de 0,3 mg/kg/j (classiquement 20 mg/j chez l'adulte), en perfusion continue de préférence.
Chez les patients recevant auparavant de la morphine par voie orale, la posologie initiale quotidienne sera le tiers de la dose orale administrée. Si la posologie orale était insuffisante, il est possible de passer d'emblée à une posologie supérieure (Voir adaptation posologique).
Chez les patients présentant des douleurs d'intensité variable dans la journée, il est possible d'utiliser un système d'analgésie contrôlée par le patient; une perfusion continue (à la posologie habituelle) sera associée à des bolus auto-administrables, équivalent à environ une heure de perfusion. Chaque bolus sera suivi d'une période sans injection possible («période réfractaire») de 10 minutes minimum.

Voie péridurale, intrathécale et intraventriculaire :
Au cours des douleurs chroniques, il sera fait appel à ces voies lorsque les autres modes d'administration sont responsables d'effets indésirables inacceptables
A titre indicatif:

- la posologie quotidienne initiale par voie péridurale, répartie en une ou deux injections, est d'environ 1/10ème de la posologie parentérale,

- la posologie quotidienne initiale par voie intrathécale, répartie en une ou deux injections, est 1/100ème de la posologie parentérale,

- la voie intraventriculaire est exceptionnellement utilisée, par certains spécialistes (la posologie de départ est de l'ordre de 0,1 à 0,2 mg/24h).

Adaptation posologique:
Fréquence de l'évaluation (degré de soulagement de la douleur, présence d'effet indésirable).
Il ne faut pas s'attarder sur une posologie qui s'avère inefficace. Le patient doit donc être vu de manière rapprochée principalement à l'instauration du traitement, tant que la douleur n'est pas contrôlée.
Augmentation de la posologie
Si la douleur n'est pas contrôlée, il convient d'augmenter la posologie quotidienne de morphine d'environ 30 à 50 %.
Dans ce processus d'ajustement des doses, il n'y a pas de limite supérieure tant que les effets indésirables peuvent être contrôlés.

4.3. Contre-indications

Ce médicament ne doit jamais être utilisé dans les cas suivants :

- hypersensibilité à la substance active ou à l’un des excipients mentionnés à la rubrique 6.1,

- insuffisance respiratoire décompensée (en l'absence de ventilation artificielle),

- insuffisance hépatocellulaire sévère (avec encéphalopathie),

- en aigu: traumatisme crânien et hypertension intracrânienne en l'absence de ventilation contrôlée,

- épilepsie non contrôlée,

- associations avec la buprénorphine, la nalbuphine, la pentazocine et la naltrexone (voir rubrique 4.5),

- allaitement, en cas d'instauration ou de poursuite après la naissance d'un traitement au long cours.

Contre-indications liées aux voies péridurale, intrathécale et intraventiculaire :

- trouble de l'hémostase au moment de la réalisation de l'acte,

- infections locales cutanées, régionales ou générales, en évolution,

- hypertension intracrânienne évolutive.

4.4. Mises en garde spéciales et précautions d'emploi

Mises en garde spéciales

En raison de sa concentration, la présentation en ampoule de 10 ml est destinée à des patients en cours de traitement morphinique et particulièrement adaptée aux techniques d'administration continue chez l'adulte.
Dans le contexte du traitement de la douleur, l'augmentation des doses, même si celles-ci sont élevées, ne relève pas le plus souvent d'un processus d'accoutumance.
Une demande pressante et réitérée nécessite de réévaluer fréquemment l'état du patient. Elle témoigne le plus souvent d'un authentique besoin en analgésique, à ne pas confondre avec un comportement addictif.
La morphine est un stupéfiant pouvant donner lieu, en dehors de son utilisation dans le traitement de la douleur, à une utilisation détournée (mésusage): dépendance physique et psychique peuvent alors s'observer, ainsi qu'une tolérance (accoutumance) se développant à la suite d'administrations répétées.
Des antécédents de toxicomanie ne contre-indiquent toutefois pas la prescription de morphine si celle-ci apparaît indispensable au traitement de la douleur.
En fonction de la durée du traitement, de la dose administrée et de l'évolution de la douleur, l'arrêt de la morphine pourra être réalisé de manière progressive pour éviter un syndrome de sevrage. Le syndrome de sevrage est caractérisé par les symptômes suivants: anxiété, irritabilité, frissons, mydriase, bouffées de chaleur, sudation, larmoiement, rhinorrhée, nausée, vomissements, crampes abdominales, diarrhées, arthralgies.
L'utilisation de morphine injectable doit s'accompagner d'une surveillance de l'intensité de la douleur, de la vigilance et de la fonction respiratoire, de manière d'autant plus rapprochée qu'il s'agit d'une douleur aiguë, que l'instauration du traitement est récente et que la voie est centrale. La somnolence constitue un signe d'appel de décompensation respiratoire.
En cas de dilution, une solution isotonique doit être utilisée.

Précautions d'emploi

La morphine doit être utilisée avec précaution dans les cas suivants:
Hypovolémie :
En cas d'hypovolémie, la morphine peut induire un collapsus. L'hypovolémie sera donc corrigée avant l'administration de morphine.
Insuffisance rénale :
L'élimination rénale de la morphine, sous la forme d'un métabolite actif, impose de débuter le traitement à posologie réduite, en adaptant par la suite, comme chez tout patient, les doses ou la fréquence d'administration à l'état clinique.
Lorsque l'étiologie de la douleur est traitée simultanément :
Il convient alors d'adapter les doses de morphine aux résultats du traitement appliqué.
Chez l'insuffisant respiratoire :
La fréquence respiratoire sera surveillée attentivement. La somnolence constitue un signe d'appel d'une décompensation.
Il importe de diminuer les doses de morphine lorsque d'autres traitements antalgiques d'action centrale sont prescrits simultanément, car cela favorise l'apparition brutale d'une insuffisance respiratoire.
Chez l'insuffisant hépatique :
L'administration de morphine doit être prudente et accompagnée d'une surveillance clinique.
Chez les personnes âgées et très âgées :
Leur sensibilité particulière aux effets antalgiques mais aussi aux effets indésirables centraux (confusion) ou digestifs, associée à une baisse physiologique de la fonction rénale, doit inciter à la prudence, en réduisant notamment la posologie initiale de moitié.
Une pathologie urétro-prostatique ou vésicale, fréquente dans cette population, expose au risque de rétention urinaire.
Les co-prescriptions de traitements psychotropes, dépresseurs du SNC ou avec un effet anti-cholinergique augmentent la survenue d'effets indésirables.
Constipation :
Il est impératif de s'assurer de l'absence de syndrome occlusif avant de mettre en route le traitement. La constipation est un effet indésirable connu de la morphine. Un traitement préventif doit être systématiquement prescrit.
Chez le nourrisson, surtout avant trois mois :

Les effets de la morphine sont plus intenses et prolongés par défaut de maturation de son métabolisme. Les doses initiales doivent être réduites. La surveillance se fera en unité de soins intensifs pour le traitement des douleurs aiguës. L'instauration d'un traitement chronique doit se faire sous surveillance hospitalière.

Hypertension intracrânienne :

En cas d'augmentation de la pression intracrânienne, l'utilisation de la morphine au cours des douleurs chroniques devra être prudente.

Troubles mictionnels:

Il existe un risque de dysurie ou de rétention d'urine principalement avec les voies intrathécale et péridurale.

Sportifs :

L'attention des sportifs doit être attirée sur le fait que cette spécialité contient de la morphine et que ce principe actif est inscrit sur la liste des substances dopantes.

Sodium

Ce médicament contient du sodium.

Ampoule de 1 ou 5 mL : Le taux de sodium est inférieur à 1 mmol (23 mg) par ampoule, c'est-à-dire « sans sodium ».

Ampoule de 10 mL : Ce médicament contient 1,28 mmol (ou 29,5 mg) de sodium par ampoule. A prendre en compte chez les patients contrôlant leur apport alimentaire en sodium.

4.5. Interactions avec d'autres médicaments et autres formes d'interactions

Il faut prendre en compte le fait que de nombreux médicaments ou substances peuvent additionner leurs effets dépresseurs du système nerveux central et contribuer à diminuer la vigilance. Il s'agit des dérivés morphiniques (analgésiques, antitussifs et traitements de substitution), des neuroleptiques, des barbituriques, des benzodiazépines, des anxiolytiques autres que les benzodiazépines (par exemple le méprobamate), des hypnotiques, des antidépresseurs sédatifs (amitriptyline, doxépine, miansérine, mirtazapine, trimipramine), des antihistaminiques H1 sédatifs, des antihypertenseurs centraux, du baclofène et du thalidomide.

Associations contre-indiquées

+ Morphiniques agonistes-antagonistes (buprénorphine, nalbuphine, pentazocine)

Diminution de l'effet antalgique ou antitussif, par blocage compétitif des récepteurs, avec risque d'apparition d'un syndrome de sevrage.

+ Naltrexone

Risque de diminution de l'effet antalgique.

Associations déconseillées

+ Consommation d'alcool

Majoration par l'alcool de l'effet sédatif de ces substances. L'altération de la vigilance peut rendre dangereuses la conduite de véhicules et l'utilisation de machines.

Eviter la prise de boissons alcoolisées et de médicaments contenant de l'alcool.

Associations faisant l'objet de précautions d'emploi

+ Rifampicine

Diminution des concentrations et de l'efficacité de la morphine et de son métabolite actif.

Surveillance clinique et adaptation éventuelle de la posologie de la morphine pendant le traitement par la rifampicine et après son arrêt.

Associations à prendre en compte

+ Autres analgésiques morphiniques agonistes

Alfentanil, codéine, dextromoramide, dextropropoxyphène, dihydrocodéine, fentanyl, oxycodone, pethidine, phénopéridine, remifentanil, sufentanil, tramadol

+ Antitussifs morphine-like

Dextrométorphane, noscapine, pholcodine
+ Antitussifs morphiniques vrais
Codéine, éthylmorphine
+ Barbituriques
+ Benzodiazépines et apparentés
Risque majoré de dépression respiratoire, pouvant être fatale en cas de surdosage.
+ Autres médicaments sédatifs
Majoration de la dépression centrale. L'altération de la vigilance peut rendre dangereuses la conduite de véhicules et l'utilisation de machines.

4.6. Fertilité, grossesse et allaitement

Grossesse

Les études effectuées chez l'animal n'ont pas mis en évidence un effet tératogène de la morphine.
En clinique, aucun effet malformatif particulier de la morphine n'est apparu à ce jour.
Toutefois, seules des études épidémiologiques permettraient de vérifier l'absence de risque.
Des posologies élevées, même en traitement bref juste avant ou pendant l'accouchement, sont susceptibles d'entraîner une dépression respiratoire chez le nouveau-né. Par ailleurs, en fin de grossesse, la prise chronique de morphine par la mère, et cela quelle que soit la dose, peut être à l'origine d'un syndrome de sevrage chez le nouveau-né. Dans ces conditions d'utilisation, une surveillance néonatale sera envisagée.
En conséquence, sous réserve de ces précautions, la morphine peut être prescrite si besoin au cours de la grossesse.

Allaitement

- une dose unique apparaît sans risque pour le nouveau-né,
- en cas d'administration répétée sur quelques jours, suspendre momentanément l'allaitement,
- en cas d'instauration ou de poursuite après la naissance d'un traitement au long cours, l'allaitement est contre-indiqué.

4.7. Effets sur l'aptitude à conduire des véhicules et à utiliser des machines

En raison de la baisse possible de vigilance induite par ce médicament, l'attention est attirée sur les risques liés à la conduite d'un véhicule et à l'utilisation d'une machine, principalement à l'instauration du traitement et en cas d'association avec d'autres dépresseurs du système nerveux central.

4.8. Effets indésirables

Parmi les effets indésirables les plus fréquents lors de l'initiation du traitement, la somnolence, une confusion, des nausées et vomissements sont rapportés. Ils peuvent être transitoires mais leur persistance doit faire rechercher une cause associée ou un surdosage. La constipation en revanche ne cède pas à la poursuite du traitement. Tous ces effets sont prévisibles et nécessitent d'être traités.
On peut également noter :

- sédation, excitation, cauchemars, plus spécialement chez le sujet âgé, avec éventuellement hallucinations;
- dépression respiratoire avec au maximum apnée ;
- augmentation de la pression intracrânienne, qu'il convient de traiter dans un premier temps ;
- dysurie et rétention urinaire en cas d'adénome prostatique ou de sténose urétrale ;

- sècheresse de la bouche ;

- syndrome douloureux abdominal aigu de type biliaire, évocateur d'un spasme du sphincter d'Oddi, survenant particulièrement chez les patients cholécystectomisés ;

- syndrome de sevrage à l'arrêt brutal de ce médicament: bâillements, anxiété, irritabilité, insomnie, frissons, mydriase, bouffées de chaleur, sudation, larmoiement, rhinorrhée, nausées, vomissements, anorexie, crampes abdominales, diarrhées, myalgies, arthralgies ;

- chez les sujets âgés ou insuffisants rénaux, risque exceptionnel d'apparition de myoclonies en cas de surdosage ou d'augmentation trop rapide des doses ;

- réactions anaphylactoïdes (attribuées à une libération d'histamine) ou plus rarement anaphylactiques, pouvant se manifester par des bouffées vasomotrices, un prurit, une éruption cutanée pouvant être urticarienne et/ou une hypotension artérielle. Des cas de collapsus cardio-vasculaire avec état de choc ont également été rapportés ;

- des cas de dermatites de contact ont été rapportés.

Déclaration des effets indésirables suspectés

La déclaration des effets indésirables suspectés après autorisation du médicament est importante. Elle permet une surveillance continue du rapport bénéfice/risque du médicament. Les professionnels de santé déclarent tout effet indésirable suspecté via le système national de déclaration : Agence nationale de sécurité du médicament et des produits de santé (ANSM) et réseau des Centres Régionaux de Pharmacovigilance - Site internet : www.ansm.sante.fr.

4.9. Surdosage

Symptômes
La somnolence constitue un signe d'appel précoce de l'apparition d'une décompensation respiratoire. Myosis extrême, hypotension, hypothermie, coma sont également observés.
Conduite d'urgence

- Arrêt de la morphine en cours.

- Stimulation-ventilation assistée, avant réanimation cardio-respiratoire en service spécialisé.

- Traitement spécifique par la naloxone: mise en place d'une voie d'abord avec surveillance pendant le temps nécessaire à la disparition des symptômes.

5. PROPRIETES PHARMACOLOGIQUES

5.1. Propriétés pharmacodynamiques

Classe pharmacothérapeutique : ANALGESIQUE OPIOIDE, code ATC : N02AA01 (N: système nerveux central).

Action sur le système nerveux central
La morphine est dotée d'une action analgésique dose-dépendante. Elle peut agir sur le comportement psychomoteur et provoquer, selon les doses et le terrain, sédation ou excitation.
Sur les centres respiratoires et celui de la toux, la morphine exerce, dès les doses thérapeutiques, une action dépressive. Les effets dépresseurs respiratoires de la morphine s'atténuent en cas d'administration chronique. L'action de la morphine sur le centre du vomissement, (*via* la zone chémo-réceptrice, stimulable notamment par la douleur, et le centre cochléo-vestibulaire), et sur la vidange gastrique (*cf. infra*) lui confère des propriétés émétisantes variables.
La morphine provoque enfin un myosis d'origine centrale.

Action sur le muscle lisse
La morphine diminue le tonus et le péristaltisme des fibres longitudinales et augmente le tonus des fibres circulaires, ce qui provoque un spasme des sphincters (pylore, valvule iléo-cæcale, sphincter anal, sphincter d'Oddi, sphincter vésical).

5.2. Propriétés pharmacocinétiques

Résorption

La résorption sanguine par voie péridurale (plexus veineux important) est plus rapide que par la voie intrathécale (petits capillaires médullaires), d'où une action analgésique plus longue par voie intrathécale. Par voies péridurale et intrathécale, la diffusion supraspinale est retardée.
La biodisponibilité des formes orales par rapport à celles administrées par voie sous-cutanée est de 50 %.
La biodisponibilité des formes orales par rapport à celles administrées par voie intraveineuse est de 30 %.

Distribution

Après résorption, la morphine est liée aux protéines plasmatiques dans la proportion de 30 %. La morphine traverse la barrière hématoencéphalique et le placenta.

Métabolisme

La morphine est métabolisée de façon importante en dérivés glucuronoconjugués qui subissent un cycle entéro-hépatique. Le 6-glucuronide et la normorphine sont deux métabolites actifs de la substance-mère.

Élimination

La demi-vie plasmatique de la morphine est variable (2 à 6 heures).
L'élimination des dérivés glucuronoconjugués se fait essentiellement par voie urinaire, à la fois par filtration glomérulaire et sécrétion tubulaire.
L'élimination fécale est faible (< 10 %).

5.3. Données de sécurité préclinique

Sans objet.

6. DONNEES PHARMACEUTIQUES

6.1. Liste des excipients

Chlorure de sodium, eau pour préparations injectables.

6.2. Incompatibilités

Une incompatibilité physicochimique a été documentée entre les solutions de morphine et le 5 fluorouracile (apparition de précipités).
Les solutions de morphine ne doivent pas être mélangées avec des solutions alcalines ou des solutions iodées, l'aminophylline, l'héparine, le sel de chlorothiazide, les sels de méticilline, les sels de nitrofurantoïne
Il est déconseillé de mélanger les solutions de morphine avec d'autres solutions ou médicaments à l'exception de ceux mentionnés dans la rubrique 6.6.

6.3. Durée de conservation

3 ans.
Après ouverture: le produit doit être utilisé immédiatement.

6.4. Précautions particulières de conservation

Conserver le conditionnement primaire dans l'emballage extérieur, à l'abri de la lumière.

6.5. Nature et contenu de l'emballage extérieur

1 mL en ampoule (verre). Boîte de 7, 10, 50 ou 100.
5 mL en ampoule (verre). Boîte de 1, 7, 10, 14, 28, 50 ou 100.
10 mL en ampoule (verre). Boîte de 1, 7, 10, 14, 28, 50 ou 100.

6.6. Précautions particulières d'élimination et de manipulation

Mode d'emploi pour l'ouverture des ampoules:
L'ampoule est prélimée en un point de l'étranglement. La tache colorée permet l'orientation de celle-ci. Saisir l'ampoule, le point coloré dirigé vers soi. L'ampoule s'ouvre facilement en plaçant le pouce sur le point coloré et en exerçant une flexion du haut vers le bas.
Utiliser immédiatement après rupture de l'enveloppe de verre.
Ne pas conserver une ampoule déjà entamée.
Une légère coloration jaune de la solution ne modifie ni la qualité ni l'efficacité du médicament.

7. TITULAIRE DE L'AUTORISATION DE MISE SUR LE MARCHE

LABORATOIRE AGUETTANT
1, RUE ALEXANDER FLEMING
69007 LYON

8. NUMERO(S) D'AUTORISATION DE MISE SUR LE MARCHE

- 34009 369 105 1 5 : 1 ml en ampoule (verre). Boîte de 7.
- 34009 369 106 8 3 : 1 ml en ampoule (verre). Boîte de 10.
- 34009 566 949 8 3 : 1 ml en ampoule (verre). Boîte de 50.
- 34009 566 945 2 5 : 1 ml en ampoule (verre). Boîte de 100.
- 34009 369 108 0 5 : 5 ml en ampoule (verre). Boîte de 1.
- 34009 369 109 7 3 : 5 ml en ampoule (verre). Boîte de 7.

ANNEXE 9 : DETAIL DU BUDGET ADAMOPA

(Etude Médicament de phase IV)
ETUDE RANDOMISEE – double aveugle

BUDGET : 249 957 € (avec frais de gestion de 10%)

« Analgésie des douleurs aiguës aux urgences : Essai randomisé de non infériorité de la Morphine seule versus l'association Morphine et Paracétamol»

Durée étude : 30 mois

Durée de recrutement : 30 mois

Nb de patients : 572

Suivi par patient : 1 jour

Nb de centres : 8 (Nantes, La Roche/Yon, Brest, Tours, Grenoble, Chateaubriand, Bordeaux, Angers)

Question posée : Intérêt ou non d'utiliser l'association morphine et paracétamol (pratique actuelle) versus la morphine seule dans la prise en charge de la douleur modérée à sévère aux urgences

Fourniture aux centres du paracétamol et du placebo avec étiquetage 'essais cliniques'.

❖ **TITRE I – Dépenses de personnel affectés à la réalisation du projet**

➢ TEC : 34 €/heure : **132 348 €**

Tâches	***Temps***	***Coût pour 8 centres***
Temps prise connaissance étude	*5 h/centre*	*1 360 €*
Temps mise en place étude	*forfait de 300 €/centre*	*2 400 €*
Suivi et Saisie Patient dans e-CRF	*visite d'inclusion : 2h/patient*<br>*Suivi mesure signes vitaux jusque T60min après l'injection du traitement : 1h/patient*<br>*Suivi jusque T60min après l'injection du traitement pour e-CRF: 2h/patient*<br>*Suivi jusque la sortie des urgences : 1h/patient*	*116 688 €*
Gestion des stocks des Unités de traitement sur site	*1h par mois par centre pendant 30 mois*	*8 160 €*
Gestion des EIG	*1h/EIG. 30 EIGs estimés pour l'étude*	*1 020 €*
Temps pour les 2 visites de	*3h par visite par centre*	*1 632 €*

Monitoring (Préparation / Réponse aux queries)		
Temps pour la visite de monito/clôture	*4h par visite par centre*	*1 088€*

- CEC : ***20 188 €***

Tâches	***Temps***	***Coût***
coordination de l'équipe médicale, élaboration maquette CRF, rédaction de newsletters	*15% d'1 ETP pendant 2 ans et demi.*	*20 188 €*

- ARC monitoring : 262.5 €/jour : **18 900 €**

Tâches	*Temps*	*Coût*
Temps prise connaissance étude	*1 jour/ARC soit 2 jours au total*	*525 €*
Participation Création/Relecture/Test CRF, guide de monitoring, guide de saisie	*2 jours*	*525 €*
Temps pour la MEP	*1.5 j pour Nantes, La Roche/Yon, Chateaubriand et Angers*<br>*2 j pour Brest, Grenoble, Tours et Bordeaux*	*3675 €*
Temps pour les visites de Monitoring (2) et la dernière visite de monitoring/clôture	*3 visites/centre*<br><br>*(1.5 j pour Nantes, La Roche/Yon, Chateaubriand et Angers*<br>*2.5 j pour Brest, Grenoble, Tours et Bordeaux)*	*14 175 €*

- Data-manager : **8 171 €** (devis)
- Pharmacie : **22 328 €** (devis pour centralisation préparation/envoi des traitements aux centres et forfaits pour les centres associés)
- Bio-statisticien : **7 400 €** (devis)

❖ **TITRE II – Dépenses à caractère médical pour la réalisation du projet**

- Surcoûts de pharmacie : achat poches de paracétamol, poches de NaCl, poches de masquage et coffrets (cartonnage) : **1 802 €**

❖ **TITRE III – Dépenses à caractère hôtelier et général pour la réalisation du projet**

- Surcoûts liés aux frais d'affranchissement pour les besoins du projet : **2 710 €**
    - envoi poches de traitement à température ambiante (50 envois environ) : 2650 €
    - envoi dossier au cpp : 60 €
- Surcoûts liés aux frais de missions : **8 880 €**
    - Frais de missions de l'ARC de monitoring : 7 880 €

**frais de déplacement**	**MEP**	**3 MONITO (dont1 monito/clôture)**	**TOTAUX frais**
TOURS	177	531	
BREST	289	867	
GRENOBLE	775	2325	
LA ROCHE/YON	51	153	
CHATEAUBRIAND	60	180	
ANGERS	60	180	
BORDEAUX	433	1299	
Abonnement			500
**TOTAUX**			7 380€

- Frais de déplacement aux congrès : 1000 €

- Surcoûts liés aux frais d'impression, de publication, pour les besoins du projet : **4 700 €**
    - impression consentements et formulaires d'EIG en duplicates (Heliographic) : 300 €

- impression lettres info patient et tryptiques (service reprographie du CHU) : 200 €
- frais de publication/traduction : 4 200 €

➢ Surcoûts liés aux frais d'archivage : **500 €**

➢ Autres dépenses à caractère hôtelier et général : assurance classe II : **1 096 €**

ANNEXE 10 : LETTRES D'ENGAGEMENT DES CENTRES ASSCOCIES

Document à annexer au protocole

Lettre d'engagement d'un investigateur associé à un projet déposé au PHRC Interrégional 2018 du Grand Ouest

Je soussigné(e).Said LARIBI...................., déclare avoir pris connaissance du projet intitulé Analgésie des Douleurs Aigues aux urgences : Essai randomisé de non infériorité de la Morphine
versus Morphine + Paracétamol et porté par l'investigateur coordonnateur, en la personne de LONGO Celine............. .

J'accepte de participer à cette étude en tant qu'investigateur associé responsable du centre de *(préciser le service, le nom de l'établissement et la ville)* Urgences, CHU de Tours.................

Je m'engage à réaliser tous les efforts possibles pour assurer le recrutement de (*préciser le nombre*) 72........patients, conformément à la version actuelle du protocole.

Date : 24/08/2018.......

Visa (*indiquer le nom de l'investigateur validant cette lettre) : Said LARIBI* .....

Professeur LARIBI
Chef de Service
SAMU 37 - URGENCES
CHRU Tours

PHRC Interrégional 2018 – GIRCI GO

Document à annexer au protocole

Lettre d'engagement d'un investigateur associé à un projet déposé au PHRC Interrégional 2018 du Grand Ouest

Je soussigné(e)...Fradin P....., déclare avoir pris connaissance du projet intitulé ADAMOPA.... et porté par l'investigateur coordonnateur, en la personne de .[illegible]

J'accepte de participer à cette étude en tant qu'investigateur associé responsable du centre de *(préciser le service, le nom de l'établissement et la ville)* ..SAMU 85... CHD Vendée

Je m'engage à réaliser tous les efforts possibles pour assurer le recrutement de (*préciser le nombre*)....5..patients, conformément à la version actuelle du protocole.

Date :...21 / 08 / 2018

Visa (*indiquer le nom de l'investigateur validant cette lettre) :* .....

Docteur Philippe FRADIN
Urgences SAMU SMUR
Centre Hospitalier Départemental
85925 LA ROCHE SUR YON Cedex 9

PHRC Interrégional 2018 – GIRCI GO

Document à annexer au protocole

Lettre d'engagement d'un investigateur associé à un projet déposé au PHRC Interrégional 2018 du Grand Ouest

Je soussigné, Dr Nicolas Hourdin, déclare avoir pris connaissance du projet intitulé **ADAMOPA** et porté par l'investigateur coordonnateur, en la personne de ………….. .

J'accepte de participer à cette étude en tant qu'investigateur associé responsable du service des Urgences du centre hospitalier de Châteaubriant.

Je m'engage à réaliser tous les efforts possibles pour assurer le recrutement de …….patients, conformément à la version actuelle du protocole.

le 29/08/18

Visa (*indiquer le nom de l'investigateur validant cette lettre) :* …..

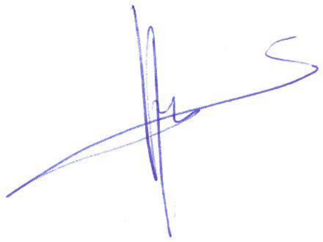

PHRC Interrégional 2018 – GIRCI GO

Document à annexer au protocole

Lettre d'engagement d'un investigateur associé à un projet déposé au PHRC Interrégional 2018 du Grand Ouest

Je soussigné(e). Dr Maxime MAIGNAN, déclare avoir pris connaissance du projet intitulé ADAMOPA et porté par l'investigateur coordonnateur, en la personne de Celine LONGO.

J'accepte de participer à cette étude en tant qu'investigateur associé responsable du centre de *(préciser le service, le nom de l'établissement et la ville)* Urgences du CHU GRENOBLE ALPES.

Je m'engage à réaliser tous les efforts possibles pour assurer le recrutement de (*préciser le nombre*) 71.4 patients, conformément à la version actuelle du protocole.

Date : 03/09/2018

Visa (*indiquer le nom de l'investigateur validant cette lettre) :* Maxime MAIGNAN

Document à annexer au protocole

Lettre d'engagement d'un investigateur associé à un projet déposé au PHRC Interrégional 2018 du Grand Ouest

Je soussigné, Pr Pierre-Marie ROY, déclare avoir pris connaissance du projet intitulé **ADAMOPA** et porté par l'investigateur coordonnateur, en la personne de **Dr Céline LONGO.**

J'accepte de participer à cette étude en tant qu'investigateur associé responsable du centre de *ANGERS, Département de Médecine d'Urgence, CHU.*

Je m'engage à réaliser tous les efforts possibles pour assurer le recrutement de 72 patients, conformément à la version actuelle du protocole.

Date :…….06|09|2018

Visa (*indiquer le nom de l'investigateur validant cette lettre) :* …..

Professeur P.M. ROY
Thérapeutique et Médecine d'Urgence
N° RPPS [illegible]
Responsable du Département de Médecine d'Urgence
CHU ANGERS

PHRC Interrégional 2018 – GIRCI GO

Document à annexer au protocole

Lettre d'engagement d'un investigateur associé à un projet déposé au PHRC Interrégional 2018 du Grand Ouest

Je soussigné, Professeur Michel Galinski, déclare avoir pris connaissance du projet intitulé **ADAMOPA** et porté par l'investigateur coordonnateur, en la personne du Dr Celine LONGO.

J'accepte de participer à cette étude en tant qu'investigateur associé responsable du centre de *la* structure d'urgence de l'hôpital Pellegrin à Bordeaux (33000).

Je m'engage à réaliser tous les efforts possibles pour assurer le recrutement de 71 patients, conformément à la version actuelle du protocole.

Date : 7 septembre 2018

Michel Galinski

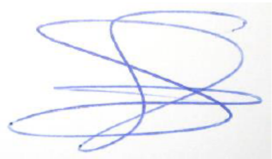

Lettre d'engagement d'un investigateur associé à un projet déposé au PHRC Interrégional 2018 du Grand Ouest

Je soussigné(e). Dr QUERELLOU Emgan déclare avoir pris connaissance du projet intitulé **ADAMOPA** et porté par l'investigateur coordonnateur, en la personne de **Céline LONGO**

J'accepte de participer à cette étude en tant qu'investigateur associé responsable du centre de *(préciser le service, le nom de l'établissement et la ville)* **Brest**

Je m'engage à réaliser tous les efforts possibles pour assurer le recrutement de (*préciser le nombre*)**72** patients, conformément à la version actuelle du protocole.

Date : 10/09/2018

Visa (*indiquer le nom de l'investigateur validant cette lettre) : Dr Emgan QUERRELOU*

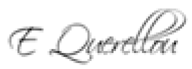

Supplement: Supplementary file 3 — Additional file 3. [file 13063_2022_6943_MOESM3_ESM.pdf]
